# Inference in skew generalized t-link models for clustered binary outcome via a parameter-expanded EM algorithm

**DOI:** 10.1371/journal.pone.0249604

**Published:** 2021-04-06

**Authors:** Chénangnon Frédéric Tovissodé, Aliou Diop, Romain Glèlè Kakaï

**Affiliations:** 1 Laboratoire de Biomathématiques et d’Estimations Forestières, Faculté des Sciences Agronomiques, Université d’Abomey-Calavi, Abomey-Calavi, Bénin; 2 Laboratoire d’Etudes et Recherches en Statistiques et Développement, Université Gaston Berger de Saint-Louis, Saint-Louis, Sénégal; University of Essex, UNITED KINGDOM

## Abstract

Binary Generalized Linear Mixed Model (GLMM) is the most common method used by researchers to analyze clustered binary data in biological and social sciences. The traditional approach to GLMMs causes substantial bias in estimates due to steady shape of logistic and normal distribution assumptions thereby resulting into wrong and misleading decisions. This study brings forward an approach governed by skew generalized t distributions that belong to a class of potentially skewed and heavy tailed distributions. Interestingly, both the traditional logistic and probit mixed models, as well as other available methods can be utilized within the skew generalized t-link model (SGTLM) frame. We have taken advantage of the Expectation-Maximization algorithm accelerated via parameter-expansion for model fitting. We evaluated the performance of this approach to GLMMs through a simulation experiment by varying sample size and data distribution. Our findings indicated that the proposed methodology outperforms competing approaches in estimating population parameters and predicting random effects, when the traditional link and normality assumptions are violated. In addition, empirical standard errors and information criteria proved useful for detecting spurious skewness and avoiding complex models for probit data. An application with respiratory infection data points out to the superiority of the SGTLM which turns to be the most adequate model. In future, studies should focus on integrating the demonstrated flexibility in other generalized linear mixed models to enhance robust modeling.

## Introduction

Binary outcomes are prominent in many applied sciences, including but not limited to biological and social sciences. Moreover, in cross sectional as well as panel studies, dichotomous responses are often naturally grouped by sampling techniques or some properties of the sampling units [1]. The preferred modern method to analyze clustered binary data is through the Generalized Linear Mixed Model (GLMM) framework [2]. Indeed, when a binary outcome has been recorded repeatedly or in the presence of latent factors, GLMMs allow accounting explicitly for over-dispersion and correlation within clusters using random effects.

Let *Y*_*ij*_ denote the binary outcome (0 or 1) of the *j*^*th*^ measurement (*j* = 1, 2, ⋯, *n*_*i*_) and **Y**_*i*_ the collection of responses from the *i*^*th*^ cluster, *i.e*. $Y_{i}={\left(Y_{i1},\ldots ,Y_{in_{i}}\right)}^{⊤}$, *i* = 1, 2, ⋯, *n*. In terms of an underlying latent continuous random vector $Z_{i}={\left(Z_{i1},\ldots ,Z_{in_{i}}\right)}^{⊤}$ and random effects **b**_*i*_ = (*b*_*i*1_, ⋯, *b*_*iq*_)^⊤^, the mixed probit model (PM) assumes that *Y*_*ij*_ are conditionally independent and given as [3]:

$$\begin{array}{ccc} Y_{ij}=I_{\left(0,\infty \right)}\left(Z_{ij}\right),Z_{i}|b_{i} & \overset{ind}{∼} & N_{n_{i}}\left(\eta_{i},I_{n_{i}}\right)\\ b_{i} & \overset{ind}{∼} & N_{q}\left(0,D\right) \end{array}$$ (1)

where *I*_*A*_(*x*) is the indicator function which equals to 1 if *x* ∈ *A* and 0 otherwise; ***η***_*i*_ is the *n*_*i*_−*vector* of linear predictors, $\eta_{i}={\left(\eta_{i1},\ldots ,\eta_{in_{i}}\right)}^{⊤}=X_{i}\beta +W_{i}b_{i}$; ***β*** is the *p*−*vector* of fixed effects; $X_{i}={\left(X_{i1},\ldots ,X_{in_{i}}\right)}^{⊤}$ and $W_{i}={\left(W_{i1},\ldots ,W_{in_{i}}\right)}^{⊤}$ are respectively known *n*_*i*_ × *p* and *n*_*i*_ × *q* matrices of covariates with **X**_*ij*_ = (*X*_*ij*1_, ⋯, *X*_*ijp*_)^⊤^ and ***W***_*ij*_ = (*W*_*ij*1_, ⋯, *W*_*ijq*_)^⊤^; $I_{n_{i}}$ is the *n*_*i*_ × *n*_*i*_ identity matrix and $N_{q}\left(0,D\right)$ denotes the *q*-variate normal distribution, with null mean vector and variance-covariance an unknown *q* × *q* matrix **D**, meant to capture the dependence structure of **Y**_*i*_. The latent variable *Z*_*ij*_ serves for a convenient stochastic representation of the conditional outcome *Y*_*ij*_. Equivalently, one may write *P*(*Y*_*ij*_ = 1|**b**_*i*_) = Φ(*η*_*ij*_) with Φ(⋅) the cumulative distribution function (cdf) of the standard normal distribution, standing as the inverse link function mapping the linear predictor *η*_*ij*_ and the predicted probabilities of the outcome *Y*_*ij*_. Combined with the normality assumption on random effects, the systematic use of this link and the well known alternative, the logit link, is somewhat controversial [4, 5].

The link function indeed has a critical role in GLMMs since it heavily impacts estimates, predictions and consequently interpretations [4, 6]. As a result, in the binary generalized linear model literature, aside the logistic and probit models based on the steady shape logistic and normal distributions respectively, there has been increasing efforts to render the link function flexible. Many works have considered heavy tailed link functions, for instance the Semi-Nonparametric [7], Student-t [8] and generalized t [9] distributions, and elliptical scale mixtures [10, 11]. Indeed, the maximum likelihood estimators of logistic and probit regression models are not robust to outliers [7]. Heavy tailed links are not sensitive to outliers and thus allow outlier-robust inference. In particular, the links functions based on the Student t distribution incorporate observation-specific stochastic weights which can be used for outlier detection [7, 12]. Similarly, skew-probit [13], skew generalized t [9], asymmetric logistic [14], loglog and complementary loglog, power symmetric and reciprocal power symmetric [15] links were used among others to handle situations where the probability of a given binary response approaches zero at a different rate than it approaches one. Skew logistic distributions have also been developed (see *e.g*. [16]) and may be used with the same aim in mind. For example [9], discussed a prostate cancer study where the outcome variable *Y* represents the presence or the penetration of cancer in or near the prostate capsule of patients. The rate at which the probability of “*Y* = 1” approaches one is expected to be very different (slower) from the rate at which it approaches zero [9]. For this study, the skew generalized t-link fits best the data [9], indicating that the simultaneous use of skewed and heavy tailed link functions can lead to more effective modelling of binary data.

Furthermore, although random effects are traditionally assumed to be normally distributed in GLMMs, this may not be realistic [17, 18]. Therefore, huge efforts have been devoted to making the random effects distribution in GLMMs flexible, replacing the normal distribution with, for instance Semi-Nonparametric [19], probability integral transformation of normal [20], skew normal [21], log-normal [22, 23], Student-t [24] and scale mixtures of normal [25] distributions.

The above background demonstrates the extent to which the number of possible approaches for fitting a flexible GLMM to correlated binary outcomes goes, although none of these approaches attempts to explicitly account for skewness and tail behavior of the link function as well as the random effects distribution simultaneously. However, the misspecification of the link function or the random effects distribution can introduce substantial bias and reduce the accuracy of the mean response as well as heterogeneity estimates [6, 18]. Standing in a fully parametric frame, we propose a unifying approach based on skew generalized t (SGT) distributions [26], that is the class of models including among others the normal, the skew normal and the Student t models. The use of a skew generalized t family instead of the Student t family allows to rescale fixed effects so that they have the same interpretation as in the mixed probit model in Eq (1).

Our contributions include *i*) an extension of the flexible generalized t-link model built for independent binary samples proposed by [9] to deal with dependent binary samples (mixed model); *ii*) a parameter-expanded EM algorithm [27] for computing the maximum likelihood of skew generalized t-link models for correlated binary data, extending the EM algorithm of [24] for t-link models; and *iii*) empirical Bayes estimators of skew t distributed random effects in mixed models for binary data.

The organization of the paper is as follows. Section 2 presents preliminary results on the SGT distributions and the truncated SGT distributions and their first two moments. Section 3 specifies the SGT-link model (SGTLM) and describes maximum likelihood estimation and cluster-specific inference based on random effects and weights. A simulation study assessing the relative performance of the SGTLM relative to existing methods and the application of the modelling approach to a real respiratory infection data are presented in Section 4. Concluding remarks are given in section 5.

## Preliminary results

In this section, we present some useful properties of the skew generalized t distributions.

### Multivariate skew generalized t distributions

Multivariate skew generalized t (SGT) distributions are special cases of multivariate skew scale mixture of normal (SSMN) distributions [28] (pages 102-103) which we first introduce. A random variable **Z** is said to follow a *p*−*variate* SSMN distribution with location ***μ***, scale **Ω**, and shape **λ**, if it can be represented as [29] (page 20, Eq 3.12):

$$\begin{array}{c} Z=\mu +U^{-1/2}\left(\delta Z_{0}+X\right),\;\;Z_{0}∼\text{HN}\left(0,1\right),\;\;X∼N_{p}\left(0,\bar{\Omega }\right) \end{array}$$ (2)

where *U*, called scale mixing variable, is a positive random variable with cdf ***F***_*U*_(⋅|***ν***) indexed by a parameter vector ***ν***, HN(0,1) is the standard half normal distribution; *Z*_0_, **X** and *U* are independent; $\bar{\Omega }=\Omega -\delta \delta^{⊤}$ and ***δ*** = (1 + **λ**^⊤^ **λ**)^−1/2^ **Ω**^1/2^ **λ**. Different choices of the scale mixing distribution *F*_*U*_(⋅|***ν***) result in various sub-classes of skew elliptical distributions, for instance, the skew normal when *P*(*U* = 1) = 1 [28] (page 103); the skew contaminated normal when ***ν*** = (*ν*_1_, *ν*_2_)^⊤^, 0 < *ν*_1_ < 1, 0 < *ν*_1_ ≤ 1 and *U* is discrete and takes the values *U* = 1 with probability 1 − *ν*_1_ and *U* = *ν*_2_ with probability *ν*_1_ [30] (page 308); the skew slash when U∼Beta(ν,1), *ν* > 0 [30] (page 307); and the skew generalized t when ***ν*** = (*ν*, *ν*_0_)^⊤^, *ν* > 0, *ν*_0_ > 0, $U∼Gamma(\nu /2,\nu_{0}/2)$ [28] (page 105). The following result states conditions for the identifiability of SSMN distributions, a requirement for reliable inference using this class of distributions.

**Lemma 1 (see** S1 Appendix **for a proof)** *The free parameters* (***μ***, ***δ***, **Ω** *and* ***ν***) *of a SSMN distribution with the representation in* Eq (2) *are identifiable if and only if i) the scale mixing distribution F*_*U*_(⋅|*ν*) *is identifiable and ii)* *F*_*U*_(⋅|*ν*) *does not satisfy* $F_{U}\left(u|\nu \right)=H\left(\frac{u}{\nu_{k}}|\nu_{-k}\right)$ *for any element ν*_*k*_ *of **ν** and any distribution function H*(⋅|*ν*_−*k*_) *where **ν***_−*k*_ *is the vector **ν** without the element ν*_*k*_. *If U has a probability density function (pdf) f*_*U*_(*u*|*ν*) *for all u* > 0, *then the condition ii*) *is equivalent to f*_*U*_(⋅|*ν*) *does not satisfy* $f_{U}\left(u|\nu \right)=\frac{1}{\nu_{k}}h_{U}\left(\frac{u}{\nu_{k}}|\nu_{-k}\right)$ *for any pdf h*_*U*_(⋅|*ν*_−*k*_).

On setting $c=\sqrt{2/\pi }$ and defining the expectations ${\tilde{U}}_{t}={\text{E}}_{U}\left\{U^{-t/2}\right\}$, and assuming that ${\tilde{U}}_{t}<\infty$ for the required expectations, the first two central moments of a SSMN vector **Z** are given by [28] (pages 109-110):

$$\begin{array}{ccc} \text{E}\{Z\} & = & \mu +c{\tilde{U}}_{1}\delta \;\;\text{and} \end{array}$$ (3)

$$\begin{array}{ccc} \text{Var}\{Z\} & = & {\tilde{U}}_{2}\Omega -c^{2}{\tilde{U}}_{1}^{2}\delta \delta^{⊤}. \end{array}$$ (4)

The ability of the SSMN distributions to capture more data structure than the normal, the skew normal or the scale mixture of normals is reflected in the expressions for skewness ($\gamma_{1_{k}}$) and kurtosis ($\gamma_{2_{k}}$) indices given for the *k*^*th*^ marginal of **Z** as [31]:

$$\begin{array}{ccc} \gamma_{1_{k}} & = & \frac{c\delta_{k}}{\sigma_{{}_{k}}^{3}}[3\left({\tilde{U}}_{3}-{\tilde{U}}_{1}{\tilde{U}}_{2}\right)-\delta_{k}^{2}({\tilde{U}}_{3}-4\frac{{\tilde{U}}_{1}^{3}}{\pi })],\text{and} \end{array}$$ (5)

$$\begin{array}{ccc} \gamma_{2_{k}} & = & \frac{1}{\sigma_{{}_{k}}^{4}}[3\left({\tilde{U}}_{4}-{\tilde{U}}_{2}^{2}\right)-4c^{2}\delta_{k}^{2}{\tilde{U}}_{1}[3({\tilde{U}}_{3}-{\tilde{U}}_{1}{\tilde{U}}_{2})-\delta_{k}^{2}({\tilde{U}}_{3}-3\frac{{\tilde{U}}_{1}^{3}}{\pi })]] \end{array}$$ (6)

where *δ*_*k*_ is the *k*^*th*^ element of ***δ*** and $\sigma_{k}^{2}$ is the *k*^*th*^ diagonal element of the covariance matrix given in Eq (4).

We notice from the expressions for skewness Eq (5) and kutosis Eq (6) indices that the parameter **λ** controls the shape of the distribution only through the working shape parameter ***δ*** = (1 + **λ**^⊤^ **λ**)^−1/2^ **Ω**^1/2^ **λ**. This quantity is invariant under marginalization, *i.e*. by the stochastic representation in Eq (2), for any arbitrary subset of **Z**, the working shape parameter is the corresponding subset of ***δ***. It is worth noticing however that the quantity ***δ*** cannot be specified unrestrictedly, independently of **Ω**. Indeed, we observe that ***δ*** = (1 + **λ**^⊤^ **λ**)^−1/2^ **Ω**^1/2^ **λ** implies that **λ** = (1 + **λ**^⊤^ **λ**)^1/2^ **Ω**^−1/2^ ***δ***. This in turn gives **λ**^⊤^ **λ** = (1 + **λ**^⊤^ **λ**)*δ*^⊤^ **Ω**^−1^ ***δ*** which yields **λ**^⊤^ **λ**(1−*δ*^⊤^ **Ω**^−1^ ***δ***) = *δ*^⊤^ **Ω**^−1^ ***δ***. We then get $\lambda^{⊤}\lambda =\frac{\delta^{⊤}\Omega^{-1}\delta }{1-\delta^{⊤}\Omega^{-1}\delta }$ so that $1+\lambda^{⊤}\lambda =\frac{1}{\left(1-\delta^{⊤}\Omega^{-1}\delta \right)}$, *i.e*. 1 + **λ**^⊤^ **λ** = (1 − *δ*^⊤^ **Ω**^−1^ ***δ***)^−1^ provided ***δ***^⊤^ **Ω**^−1^ ***δ*** ≠ 1. Therefore, **λ** is recorvered from ***δ*** and **Ω** under the constraint ***δ***^⊤^ **Ω**^−1^ ***δ*** < 1 as:

$$\begin{array}{c} \lambda ={\left(1-\delta^{⊤}\Omega^{-1}\delta \right)}^{-1/2}\Omega^{-1/2}\delta . \end{array}$$ (7)

It is nevertheless possible to unrestrictedly specify ***δ*** and $\bar{\Omega }$ (positive definite). In this case, **Ω** is recovered as $\Omega =\bar{\Omega }+\delta \delta^{⊤}$. Using the Sherman-Morrison identity [32] (page 121, Eq 3.1), we have $\Omega^{-1}={\left(\bar{\Omega }+\delta \delta^{⊤}\right)}^{-1}={\bar{\Omega }}^{-1}-\frac{{\bar{\Omega }}^{-1}\delta \delta^{⊤}{\bar{\Omega }}^{-1}}{1+\delta^{⊤}{\bar{\Omega }}^{-1}\delta }$ from which we get $\delta^{⊤}\Omega^{-1}\delta =\delta^{⊤}{\bar{\Omega }}^{-1}\delta -\frac{\delta^{⊤}{\bar{\Omega }}^{-1}\delta \delta^{⊤}{\bar{\Omega }}^{-1}\delta }{1+\delta^{⊤}{\bar{\Omega }}^{-1}\delta }$ that simplifies as $\delta^{⊤}\Omega^{-1}\delta =\frac{\delta^{⊤}{\bar{\Omega }}^{-1}\delta }{1+\delta^{⊤}{\bar{\Omega }}^{-1}\delta }$. We thus have $1-\delta^{⊤}\Omega^{-1}\delta =\frac{1}{1+\delta^{⊤}{\bar{\Omega }}^{-1}\delta }$ hence

$$\begin{array}{c} \lambda ={\left(1+\delta^{⊤}{\bar{\Omega }}^{-1}\delta \right)}^{1/2}\Omega^{-1/2}\delta \;\;\text{with}\;\;\Omega =\bar{\Omega }+\delta \delta^{⊤}. \end{array}$$ (8)

In the binary data modeling framework, we shall consider ***δ*** and $\bar{\Omega }$ as model parameters as they turn to be easier to estimate by the mean of the EM algorithm. For the multivariate Skew Generalized t (SGT) distribution, the mixing variable *U* is gamma distributed, *i.e*. $U∼Gamma(\nu /2,\nu_{0}/2)$ with pdf [33] (page 1, Eq 1):

$$\begin{array}{c} f_{G}\left(u|\nu /2,\nu_{0}/2\right)=\frac{{\left(\frac{\nu_{0}}{2}\right)}^{\frac{\nu }{2}}}{\Gamma (\frac{\nu }{2})}u^{\frac{\nu }{2}-1}\;\text{exp}(-\frac{\nu_{0}}{2}u)\;\text{for}\;u>0,\nu >0,\nu_{0}>0. \end{array}$$ (9)

The *p*−*variate* SGT distribution, denoted ${\text{SGT}}_{p}\left(\mu ,\Omega ,\lambda ,\nu \right)$ with ***ν*** = (*ν*, *ν*_0_)^⊤^ has pdf for $z\in R^{p}$ [28] (page 106):

$$\begin{array}{ccc} SGt_{p}\left(z|\mu ,\Omega ,\lambda ,\nu \right) & = & 2\;Gt_{p}\left(z|\mu ,\Omega ,\nu \right)\;T(\alpha {\left(\frac{p+\nu }{\nu_{0}+z_{0}^{⊤}z_{0}}\right)}^{1/2}|p+\nu ),\;\; \end{array}$$ (10)

where

$$\begin{array}{ccc} Gt_{p}\left(z|\mu ,\Omega ,\nu \right) & = & \frac{\Gamma \left(\frac{p+\nu }{2}\right){\left|\Omega \right|}^{-1/2}\nu_{0}^{\nu /2}}{\Gamma \left(\nu /2\right){\left(\pi \right)}^{p/2}}{\left(\nu_{0}+z_{0}^{⊤}z_{0}\right)}^{-(p+\nu )/2} \end{array}$$ (11)

is the pdf of the *p*−*variate* Generalized t (GT) distribution, **z**_0_ = **Ω**^−1/2^ (**z** − ***μ***), *α* = **λ**^⊤^ **z**_0_, and *T*(⋅|*ν*) is the cdf of the standard univariate t distribution with *ν* degrees of freedom. For SGT distributions, the expectations ${\tilde{U}}_{t}$ required for computing moments given in Eqs (3)–(6) have for *t* < *ν* the form

$$\begin{array}{ccc} {\tilde{U}}_{t} & = & {\left(\frac{\nu_{0}}{2}\right)}^{t/2}\frac{\Gamma (\frac{\nu -t}{2})}{\Gamma (\nu /2)}. \end{array}$$ (12)

It is worthwhile noticing that the gamma mixing pdf *f*_*G*_(⋅|*ν*/2, *ν*_0_/2) satisfies the condition *i*) of *Lemma 1* but not the condition *ii*). The SGT ditribution with *ν* as a parameter is thus not identifiable. However, restricting *ν*_0_ to a fixed value (so that only *ν* is considered as a parameter) is sufficient to ensure identifiability of the SGT family of distribution. When *ν*_0_ = *ν*, the *p*−*variate* SGT distribution ${\text{SGT}}_{p}\left(\mu ,\Omega ,\lambda ,\nu \right)$ reduces to the *p*−*variate* Skew t (ST) distribution [28] (page 106), denoted ${\text{ST}}_{p}\left(\mu ,\Omega ,\lambda ,\nu \right)$ which is thus identifiable with pdf *St*_*p*_(⋅|***μ***, **Ω**, **λ**, *ν*) and cdf *St*_*p*_(⋅|***μ***, **Ω**, **λ**, *ν*). If **λ** = 0, the SGT distribution reduces to the GT distribution which equals the Student t distribution for *ν*_0_ = *ν*. The following lemma formalizes the relationship between skew generalized t and skew t distributions.

**Lemma 2 (see** S2 Appendix **for a proof)** *Let us consider the SGT distribution* ${\text{SGT}}_{p}(\mu ,\Omega ,\lambda ,\nu )$ *with **ν*** = (*ν*, *ν*_0_)^⊤^ *and pdf in* Eq (10). *Set* $\Omega^{*}=\frac{\nu_{0}}{\nu }\Omega$. *The following statements hold*:

1. *SGt*_*p*_(**z**|***μ***, **Ω**, **λ**, *ν*) = *St*_*p*_(**z**|***μ***, Ω*, **λ**, *ν*);
2. If $Z∼{\text{SGT}}_{p}(\mu ,\Omega ,\lambda ,\nu )$ then $Z∼{\text{ST}}_{p}(\mu ,\Omega^{*},\lambda ,\nu )$.

*Lemma 2* indicates that any SGT vector is a rescaled version of a ST vector. However, in the frame of binary data models, the use of a SGT distribution instead of a simple ST distribution as link function allows to control the scale of the link function through the parameter *ν*_0_ [9]. Specifically, a skew generalized t-link allows to define a skewed and heavy-tailled binary mixed model where fixed effects have the same scale and interpretation as in the mixed probit model in Eq (1). Interestingly, the popular logit and probit links for binary data can be recast as special cases of the cdf of the SGT class of distributions. Indeed, the normal distribution is a limiting case of SGT distributions when *ν*_0_ = *ν* → ∞ and **λ** = 0. Moreover, the logistic distribution is well appoximated by a rescaled Student t distribution with appropriate degrees of freedom [8] (page 228). These constatations make the SGT distributions appropriate for extending the traditional probit and logistic GLMMs, accounting for skewness and heavy tail behaviors. To this end, we present in the next section some results on truncated multivariate SGT distributions since binary data can reflect truncation of latent continuous variables.

### Truncated multivariate skew generalized t distributions

As seen for the mixed probit model in Eq (1), models for binary data can be defined by truncating latent variables following continuous distributions. We define in this section a class of truncated multivariate skew generalized t distributions which are useful for a latent variable representation of skew generalized t-link binary data models. We also give expressions to evaluate some joint moments of a truncated multivariate skew generalized t distribution and a gamma distribution, as they prove useful in the implementation of the EM algorithm for the skew generalized t-link model.

Let ${\text{TSGT}}_{p}\left(\mu ,\Omega ,\lambda ,\nu ,A\right)$ represent a *p*−*variate* skew generalized t (SGT) vector restricted to a *p*-dimensional hyperplane A; with ***μ*** a *p*−*vector* (location), **Ω** a *p* × *p* positive definite matrix (scale), **λ** a *p*−*vector* (shape) and ***ν*** = (*ν*, *ν*_0_)^⊤^ a vector of positive scalars (degrees of freedom). The pdf of $Z∼{\text{TSGT}}_{p}\left(\mu ,\Omega ,\lambda ,\nu ,A\right)$ is:

$$\begin{array}{c} TSGt_{p}\left(z|\mu ,\Omega ,\lambda ,\nu ,A\right)=\alpha_{st}^{-1}\;SGt_{p}\left(z|\mu ,\Omega ,\lambda ,\nu \right)\;I_{A}\left(z\right)\;\text{for}\;z\in R^{p} \end{array}$$ (13)

where *SGt*_*p*_(⋅|***μ***, **Ω**, **λ**, ***ν***) is the pdf in Eq (10) and $\alpha_{st}=\int_{A}SGt_{p}(z|\mu ,\Omega ,\lambda ,\nu )dz$ serves for normalization. The cdf of **Z** is denoted $TSGT_{p}\left(\cdot |\mu ,\Omega ,\lambda ,\nu ,A\right)$. When **λ** = 0, we obtain a truncated generalized t distribution denoted ${\text{TGT}}_{p}\left(\mu ,\Omega ,\nu ,A\right)$ with pdf $TGt_{p}\left(\cdot |\mu ,\Omega ,\nu ,A\right)$ and cdf $TGT_{p}\left(\cdot |\mu ,\Omega ,\nu ,A\right)$. When *ν*_0_ = *ν*, we get a truncated ST distribution denoted ${\text{TST}}_{p}\left(\mu ,\Omega ,\lambda ,\nu ,A\right)$ with pdf $TSt_{p}\left(\cdot |\mu ,\Omega ,\lambda ,\nu ,A\right)$ and cdf $TST_{p}\left(\cdot |\mu ,\Omega ,\lambda ,\nu ,A\right)$. If both **λ** = 0 and *ν*_0_ = *ν*, the truncated multivariate SGT distribution is reduced to a truncated multivariate t distribution [34] denoted ${\text{TT}}_{p}\left(\mu ,\Omega ,\nu ,A\right)$ with pdf $Tt_{p}\left(\cdot |\mu ,\Omega ,\nu ,A\right)$ and cdf $TT_{p}\left(\cdot |\mu ,\Omega ,\nu ,A\right)$.

In the frame of correlated binary data models, the truncation region A typically has the form $A=A_{1}\times A_{2}\times \ldots \times A_{p}$ where $A_{k}$ are real intervals of the form $A_{k}=\left(-\infty ,a_{k}\right]$ or $A_{k}=\left(a_{k},\infty \right)$, for $a_{k}\in R$ (*k* = 1, 2, ⋯, *p*). Let us consider for instance a vector **Y** of three binary outcomes obtained by truncating the elements of a 3−*variate* SGT vector $Z∼{\text{SGT}}_{3}\left(\mu ,\Omega ,\lambda ,\nu \right)$: *Y*_*k*_ = 0 if *Z*_*k*_ ≤ 0 and *Y*_*k*_ = 1 if *Z*_*k*_ > 0. In practice, however, only the binary outcomes (**Y**) are observable whereas the latent outcome **Z** is unobservable. Suppose one observes the binary outcomes **y** = (1, 0, 1)^⊤^. This implies that the corresponding value **z** of the latent vector **Z** satisfies *z*_1_ > 0, *z*_2_ ≤ 0, and *z*_3_ > 0. The conditional distribution of **Z** given **Y** = **y** (required for maximum likelihood estimation using the EM algorithm) is thus ${\text{SGT}}_{3}\left(\mu ,\Omega ,\lambda ,\nu \right)$ truncated to the region $A_{e.g.}=\left(0,\infty \right)\times \left(-\infty ,0\right]\times \left(0,\infty \right)$, *i.e*. $Z|Y=y∼{\text{TSGT}}_{p}\left(\mu ,\Omega ,\lambda ,\nu ,A_{e.g.}\right)$ as defined in Eq (13).

We shall use the simplified notation ${\text{TSGT}}_{p}\left(\mu ,\Omega ,\lambda ,\nu ,a\right)$ with $a\in R^{p}$ to denote a truncated SGT distribution ${\text{TSGT}}_{p}\left(\mu ,\Omega ,\lambda ,\nu ,A\right)$ when A is the right truncated hyperplane $A=\{z\in R^{p}|\;z_{1}\leq a_{1},\ldots ,z_{p}\leq a_{p}\}$. In this case, *α*_*st*_ = *SGT*_*p*_(**a**|***μ***, **Ω**, **λ**, ***ν***) with *SGT*_*p*_(⋅|***μ***, **Ω**, **λ**, ***ν***) the cdf of the *p*−*variate* ST distribution. This corresponds for instance to the situation where all binary outcomes are zeros. When **λ** = 0, the right truncated SGT distribution ${\text{TSGT}}_{p}\left(\mu ,\Omega ,0,\nu ,a\right)$ is a right truncated GT distribution denoted ${\text{TGT}}_{p}\left(\mu ,\Omega ,\nu ,a\right)$ whose pdf and cdf are respectively denoted *TGt*_*p*_(⋅|***μ***, **Ω**, *ν*, **a**) and *TGT*_*p*_(⋅|***μ***, **Ω**, *ν*, **a**). When *ν*_0_ = *ν*, the right truncated SGT distribution is a right truncated ST distribution denoted ${\text{TST}}_{p}\left(\mu ,\Omega ,\lambda ,\nu ,a\right)$ with pdf *TSt*_*p*_(⋅|***μ***, **Ω**, **λ**, *ν*, **a**) and cdf *TST*_*p*_(⋅|***μ***, **Ω**, **λ**, *ν*, **a**). If both **λ** = 0 and *ν*_0_ = *ν*, the distribution is reduced to a right truncated t distribution denoted ${\text{TT}}_{p}\left(\mu ,\Omega ,\nu ,a\right)$ with pdf *Tt*_*p*_(⋅|***μ***, **Ω**, *ν*, **a**) and cdf *TT*_*p*_(⋅|***μ***, **Ω**, *ν*, *a*). In the above example, if **y** = (0, 0, 0)^⊤^, then the truncation region becomes $A_{e.g.}=\left(-\infty ,0\right]\times \left(-\infty ,0\right]\times \left(-\infty ,0\right]$. Since all truncation points are zeros, we shall write in this case $Z|Y=y∼{\text{TSGT}}_{p}\left(\mu ,\Omega ,\lambda ,\nu ,a\right)$ with **a** = (0, 0, 0)^⊤^ using the above simplified notation.

The implementation of an EM algorithm for a SGT distribution based binary data model requires joint moments of the form $\bar{u_{r}}=\text{E}\left\{U^{r/2}\right\}$, $\bar{u_{r}z_{s}}=\text{E}\left\{U^{r/2}Z^{\left(s\right)}\right\}$, $\bar{\tau_{r}}=\text{E}\left\{U^{r/2}\zeta_{1}\left(U^{1/2}\alpha \right)\right\}$ and $\bar{\tau_{r}z_{s}}=\text{E}\left\{U^{r/2}\zeta_{1}\left(U^{1/2}\alpha \right)Z^{\left(s\right)}\right\}$ for *s* ∈ {1, 2}, **Z**^(1)^ = **Z** and **Z**^(2)^ = **Z** **Z**^⊤^, $U∼Gamma(\nu /2,\nu_{0}/2)$, $Z∼{\text{TSGT}}_{p}\left(\mu ,\Omega ,\lambda ,\nu ,A\right)$, *α* = **λ**^⊤^ **Ω**^−1/2^(**Z** − ***μ***), *ζ*_1_(*x*) = *ϕ*(*x*)/Φ(*x*) with *ϕ*(⋅) the pdf of the standard normal distribution, and A is an hyperplane of the form $A=A_{1}\times \ldots \times A_{p}$ with $A_{k}\in \{(-\infty ,a_{k}],(a_{k},\infty )\}$, (*a*_*k*_, ∞)} for **a** = (*a*_1_, ⋯, *a*_*p*_)^⊤^. *Proposition 1* hereafter will be useful for the derivation of $\bar{u_{r}}$, $\bar{u_{r}z_{s}}$, $\bar{\tau_{r}}$ and $\bar{\tau_{r}z_{s}}$.

**Proposition 1 (see** S3 Appendix **for a proof)** *Let* $Z∼{\text{TSGT}}_{p}\left(\mu ,\Omega ,\lambda ,\nu ,A\right)$ *with **ν*** = (*ν*, *ν*_0_)^⊤^, $U∼Gamma(\nu /2,\nu_{0}/2)$ *and set α* = **λ**^⊤^ **Ω**^−1/2^(**Z** − ***μ***). *Then, for any real r* > − ***ν** and an integrable function g*(⋅) *of* **Z**:

$$\begin{array}{ccc} \text{E}\{U^{r/2}g\left(Z\right)\} & = & C_{r}\left(\nu \right)\alpha_{st}^{-1}\alpha_{u,r}\;\text{E}\left\{g\left(Z_{u,r}\right)\right\} \end{array}$$ (14)

$$\begin{array}{ccc} \text{E}\{U^{r/2}\zeta_{1}\left(U^{1/2}\alpha \right)g\left(Z\right)\} & = & cMC_{r}\left(\nu \right)\alpha_{st}^{-1}\alpha_{\tau ,r}\;\text{E}\left\{g\left(Z_{\tau ,r}\right)\right\} \end{array}$$ (15)

*where* $C_{r}\left(\nu \right)={\left(\frac{2}{\nu_{0}}\right)}^{r/2}\frac{\Gamma (\frac{\nu +r}{2})}{\Gamma (\nu /2)}$, $\alpha_{st}=\int_{A}St_{p}(z|\mu ,\frac{\nu_{0}}{\nu }\Omega ,\lambda ,\nu )dz$, $\alpha_{\tau ,r}=\int_{A}t_{p}(z|\mu ,{\bar{\Omega }}^{*},\nu +r)dz$, *and* $\alpha_{u,r}=\int_{A}St_{p}(z|\mu ,\Omega^{*},\lambda ,\nu +r)dz$; $c=\sqrt{2/\pi }$, $Z_{u,r}∼{\text{TST}}_{p}(\mu ,\Omega^{*},\lambda ,\nu +r,A)$, $Z_{\tau ,r}∼{\text{TT}}_{p}(\mu ,{\bar{\Omega }}^{*},\nu +r,A)$, $M={\left(1+\delta {\bar{\Omega }}^{-1}\delta \right)}^{-1/2}$ *with* $\delta ={\left(\frac{\nu_{0}}{\nu }\right)}^{1/2}{\left(1+\lambda^{⊤}\lambda \right)}^{-1/2}\Omega^{1/2}\lambda$, $\bar{\Omega }=\frac{\nu_{0}}{\nu }\Omega -\delta \delta^{⊤}$, ${\bar{\Omega }}^{*}=\frac{\nu }{\nu +r}\bar{\Omega }$ *and* $\Omega^{*}=\frac{\nu_{0}}{\nu +r}\Omega$.

By the mean of a simple linear transformation, we obtain from *Proposition 1* the joint expectations $\bar{u_{r}}$, $\bar{u_{r}z_{s}}$, $\bar{\tau_{r}}$ and $\bar{\tau_{r}z_{s}}$ in terms of moments of a truncated multivariate skew t distribution.

**Corollary 1 (see** S4 Appendix **for a proof)** *Let* $Z∼{\text{TSGT}}_{p}\left(\mu ,\Omega ,\lambda ,\nu ,A\right)$ *with **ν*** = (*ν*, *ν*_0_)^⊤^, $U∼Gamma(\nu /2,\nu_{0}/2)$, $a\in R^{p}$ *and* $A=A_{1}\times \ldots \times A_{p}$ *with* $A_{k}=\left(-\infty ,a_{k}\right]$ *or* $A_{k}=\left(a_{k},\infty \right)$. *Then, on setting* $\delta ={\left(\frac{\nu_{0}}{\nu }\right)}^{1/2}{\left(1+\lambda^{⊤}\lambda \right)}^{-1/2}\Omega^{1/2}\lambda$, $\bar{\Omega }=\frac{\nu_{0}}{\nu }\Omega -\delta \delta^{⊤}$, **A** = *diag*(*A*_1_, ⋯, *A*_*p*_) *with A*_*k*_ = 1 *if* $A_{k}=\left(-\infty ,a_{k}\right]$ *and A*_*k*_ = −1 *if* $A_{k}=\left(a_{k},\infty \right)$, a* = **Aa**, μ* = **A** ***μ***, $\Omega^{*}=\frac{\nu_{0}}{\nu }A\Omega A$, ${\bar{\Omega }}^{*}=A\bar{\Omega }A$, λ* = **Aλ** *and α*_*st*_ = *ST*_*p*_(a*|μ*, Ω*, λ*, ***ν***),

$$\begin{array}{ccc} \bar{u_{r}} & = & C_{r}\left(\nu \right)\;\alpha_{st}^{-1}\;\alpha_{u,r} \end{array}$$ (16)

$$\begin{array}{ccc} \bar{u_{r}z_{1}} & = & C_{r}\left(\nu \right)\;\alpha_{st}^{-1}\;\alpha_{u,r}\;A\text{E}\left\{X_{u,r}\right\} \end{array}$$ (17)

$$\begin{array}{ccc} \bar{u_{r}z_{2}} & = & C_{r}\left(\nu \right)\;\alpha_{st}^{-1}\;\alpha_{u,r}\;A\text{E}\left\{X_{u,r}X_{u,r}^{⊤}\right\}A \end{array}$$ (18)

$$\begin{array}{ccc} \bar{\tau_{r}} & = & cMC_{r}\left(\nu \right)\;\alpha_{st}^{-1}\;\alpha_{\tau ,r} \end{array}$$ (19)

$$\begin{array}{ccc} \bar{\tau_{r}z_{1}} & = & cMC_{r}\left(\nu \right)\;\alpha_{st}^{-1}\;\alpha_{\tau ,r}\;A\text{E}\left\{X_{\tau ,r}\right\} \end{array}$$ (20)

$$\begin{array}{ccc} \bar{\tau_{r}z_{2}} & = & cMC_{r}\left(\nu \right)\;\alpha_{st}^{-1}\;\alpha_{\tau ,r}\;A\text{E}\left\{X_{\tau ,r}X_{\tau ,r}^{⊤}\right\}A \end{array}$$ (21)

*where* $X_{u,r}∼{\text{TST}}_{p}(\mu^{*},\frac{\nu }{\nu +r}\Omega^{*},\lambda^{*},\nu +r,a^{*})$, $X_{\tau ,r}∼{\text{TT}}_{p}(\mu^{*},\frac{\nu }{\nu +r}{\bar{\Omega }}^{*},\nu +r,a^{*})$, *and we have set* $\alpha_{u,r}=ST_{p}(a^{*}|\mu^{*},\frac{\nu }{\nu +r}\Omega^{*},\lambda^{*},\nu +r)$ *and* $\alpha_{\tau ,r}=T_{p}(a^{*}|\mu^{*},\frac{\nu }{\nu +r}{\bar{\Omega }}^{*},\nu +r)$.

For a practical use of *Corollary 1*, the cumulative multivariate skew t distribution is required. To this end, the function *pmst* of the package *sn* [35] in R freeware [36] is an appropriate routine.

### Moments of truncated multivariate skew generalized t distributions

The evaluation of expectations $\text{E}\{X_{u,r}^{\left(s\right)}\}$ involved in *Corollary 1* calls for general expressions for the first and second order moments of truncated multivariate SGT distributions. These moments are required in the implementation of an EM algorithm for a SGT distribution based binary data model. The moments have been derived for truncated multivariate t distributions by [34] and were used by [24] in their implementation of the EM algorithm for a t-link GLMM. We present in this section the expressions for the first two moments of the multivariate SGT distributions, relying on the Theorem 1 of [37] and the moments of truncated multivariate t distributions available from [34] (see also [38]).

Let $Z∼{\text{TSGT}}_{p}\left(\mu ,\Omega ,\lambda ,\nu ,a\right)$ with *ν* = (*ν*, *ν*_0_)^⊤^ and $a\in R^{p}$, *i.e*. a *p*−*variate* SGT vector restricted to the right truncated hyperplane $A=\{x\in R^{p}|\;x_{1}\leq a_{1},\ldots ,x_{p}\leq a_{p}\}$. The pdf of **Z** is:

$$\begin{array}{c} TSGt_{p}\left(z|\mu ,\Omega ,\lambda ,\nu ,a\right)=\alpha_{st}^{-1}\;SGt_{p}\left(z|\mu ,\Omega ,\lambda ,\nu \right)\;{\text{I}}_{A}\left(z\right) \end{array}$$ (22)

where *α*_*st*_ = *SGT*_*p*_(**a**|***μ***, **Ω**, **λ**, ***ν***), *SGT*_*p*_(⋅|***μ***, **Ω**, **λ**, ***ν***) is the cdf of the *p*−*variate* SGT distribution. If ***μ*** = 0, **Ω** is a correlation matrix (**Ω** = **R**) and *ν*_0_ = *ν*, then $Z∼{\text{TST}}_{p}\left(0,R,\lambda ,\nu ,a\right)$. In this case, the first two moments of **Z** can be evaluated using the following proposition which simply combines Theorem 3 in [34] with Theorem 1 in [37].

**Proposition 2 (see** S5 Appendix **for a proof)** *Let* $Z∼{\text{TST}}_{p}\left(0,R,\lambda ,\nu ,a\right)$ *with* **R** *a correlation matrix. Then*,

$$\begin{array}{ccc} \text{E}\{Z\} & = & \frac{\nu \alpha_{st}^{-1}}{\nu -2}[C_{0}^{*}\delta -Rq^{*}\left(a\right)]\;\text{for}\;\;\nu >2,\;\text{and}\;\text{for}\;\nu >4\; \end{array}$$ (23)

$$\begin{array}{ccc} \text{E}\{ZZ^{⊤}\} & = & \frac{\nu \alpha_{st}^{-1}}{\nu -2}[\alpha_{st}^{*}R+R\left(H^{*}+D^{*}\right)R-RH_{0}^{*}\delta^{⊤}-\delta H_{0}^{{*}^{⊤}}R+D_{0}^{*}\delta \delta^{⊤}] \end{array}$$ (24)

*where* $C_{0}^{*}=\frac{\Gamma (\frac{\nu -1}{2})}{\Gamma \left(\frac{\nu -2}{2}\right){\left(\nu \pi \right)}^{1/2}}T_{p}(a|0,\frac{\nu }{\nu -1}\left(R-\delta \delta^{⊤}\right),\nu -1)$ *with T*_*p*_(⋅|***μ***, **Ω**, *ν*) *the cdf of the p*−*variate t distribution with location **μ***, *scale* **Ω** *and degrees of freedom **ν**;* $q^{*}\left(a\right)\in R^{p}$ with *i*^*th*^ element $q_{i}^{*}\left(a_{i}\right)=\sqrt{\frac{\nu -2}{\nu }}\;t(\sqrt{\frac{\nu -2}{\nu }}a_{i},\nu -2)T_{p}({\bar{a}}_{2}^{\left(i\right)}|a_{i}{\bar{R}}_{12}^{\left(i\right)},\frac{\nu +a_{i}^{2}}{\nu -1}{\bar{R}}_{22.1}^{\left(i\right)},\nu -1)$, *t*(⋅) *being the pdf of the standard Student t distribution;* $\alpha_{st}^{*}=T_{p+1}(\bar{a}|0,\frac{\nu }{\nu -2}R^{*},\nu -2)$ *with* $R^{*}=(\begin{array}{cc} \sigma_{0}^{2} & -\sigma_{0}\delta^{⊤}\\ -\sigma_{0}\delta & R \end{array})$, $\sigma_{0}=\sqrt{1+\lambda^{⊤}\lambda }$ and $\delta =\sigma_{0}^{-1}R^{1/2}\lambda$; $H_{0}^{*}\in R^{p}$ with *i*^*th*^ *element* $H_{0i}^{*}=\frac{\nu }{\nu -4}\;t_{2}(a_{1}^{\left(0i\right)}|0,\frac{\nu }{\nu -4}R_{11}^{\left(0i\right)},\nu -4)T_{p-1}({\bar{a}}_{2}^{\left(0i\right)}|{\bar{\mu }}_{2.1}^{\left(0i\right)},\frac{\nu +\alpha_{0i}}{\nu -2}{\bar{R}}_{22.1}^{\left(0i\right)},\nu -2)$, $\alpha_{0i}=\frac{a_{i}^{2}}{\left(1-\delta_{i}^{2}\right)}$; H* *is the p* × *p matrix with diagonal elements* $H_{ii}^{*}=0$ *and off diagonal elements defined as*

$$\begin{array}{c} H_{ij}^{*}=\frac{\nu }{\nu -4}\;t_{2}(a_{1}^{\left(ij\right)}|0,\frac{\nu }{\nu -4}R_{11}^{\left(ij\right)},\nu -4)T_{p-1}({\bar{a}}_{2}^{\left(ij\right)}|{\bar{\mu }}_{2.1}^{\left(ij\right)},\frac{\nu +\alpha_{ij}}{\nu -2}{\bar{R}}_{22.1}^{\left(ij\right)},\nu -2) \end{array}$$

*with* $\alpha_{ij}=\frac{a_{i}^{2}-2\rho_{ij}a_{i}a_{j}+a_{j}^{2}}{\left(1-\rho_{ij}^{2}\right)}$; $D_{0}^{*}=\delta^{⊤}{\text{H}}_{0}^{*}$; D* *is the p* × *p diagonal matrix with diagonal elements* $D_{ii}^{*}=\delta_{i}H_{0i}^{*}-a_{i}q_{i}^{*}\left(a_{i}\right)-R_{i}H^{*i}$, H^*i^ *denoting the i*^*th*^ *column of* H*; $\bar{a}={\left(0,a\right)}^{⊤}$, $R_{11}^{\left(0i\right)}=(\begin{array}{cc} 1 & -\delta_{i}\\ -\delta_{i} & 1 \end{array})$, $R_{11}^{\left(ij\right)}=(\begin{array}{cc} 1 & \rho_{ij}\\ \rho_{ij} & 1 \end{array})$, $\bar{R}=(\begin{array}{cc} 1 & -\delta^{⊤}\\ -\delta & R \end{array})$, *with δ*_*i*_ *the i*^*th*^ *element of **δ***, *ρ*_*ij*_ *the* (*ij*)^*th*^ *element of* **R**; **H**^*i*^ *the i*^*th*^ *column of* **H**; ${\bar{a}}_{2}^{\left(i\right)}$ *the vector* **a** *with its* (*i* + 1)^*th*^ *element* (*i.e*.*a*_*i*_) *deleted;* ${\bar{R}}_{12}^{\left(i\right)}$ *the* (*i* + 1)^*th*^ *column of* $\bar{R}$ *with its* (*i* + 1)^*th*^ *element* (*i.e*. 1) *deleted;* ${\bar{R}}_{22.1}^{\left(i\right)}={\bar{R}}_{22}^{\left(i\right)}-{\bar{R}}_{12}^{\left(i\right)}{\bar{R}}_{12}^{(i)T}$, ${\bar{R}}_{22}^{\left(i\right)}$ *being* $\bar{R}$ *with its* (*i* + 1)^*th*^ *row and column deleted;* $a_{1}^{\left(ij\right)}={\left(a_{i},a_{j}\right)}^{⊤}$; ${\bar{a}}_{2}^{\left(ij\right)}$ *the vector* $\bar{a}$ *with its* (*i* + 1)^*th*^ *and* (*j* + 1)^*th*^ *elements* (*i.e*.*a*_*i*_ and *a*_*j*_) *deleted;* ${\bar{R}}_{22.1}^{\left(ij\right)}={\bar{R}}_{22}^{\left(ij\right)}-{\bar{R}}_{12}^{\left(ij\right)}{\left[R_{11}^{\left(ij\right)}\right]}^{-1}{\bar{R}}_{12}^{(ij)T}$, ${\bar{R}}_{22}^{\left(ij\right)}$ *being* $\bar{R}$ *with its* (*i* + 1)^*th*^ *and* (*j* + 1)^*th*^ *rows and columns deleted;* ${\bar{R}}_{12}^{\left(ij\right)}$ *being the matrix* $\bar{R}$ *with its* (*i* + 1)^*th*^ *and* (*j* + 1)^*th*^ *columns deleted, and only its* (*i* + 1)^*th*^ *and* (*j* + 1)^*th*^ *rows kept;* ${\bar{\mu }}_{2.1}^{\left(ij\right)}={\bar{R}}_{12}^{\left(ij\right)}{\left[R_{11}^{\left(ij\right)}\right]}^{-1}a_{1}^{\left(ij\right)}$; $a_{1}^{\left(0i\right)}={\left(0,a_{i}\right)}^{⊤}$; ${\bar{a}}_{2}^{\left(0i\right)}$ *the vector **a** with its i*^*th*^ *element* (*i.e*.*a*_*i*_) deleted; ${\bar{R}}_{22.1}^{\left(0i\right)}={\bar{R}}_{22}^{\left(0i\right)}-{\bar{R}}_{12}^{\left(0i\right)}{\left[R_{11}^{\left(0i\right)}\right]}^{-1}{\bar{R}}_{12}^{(0i)T}$, ${\bar{R}}_{22}^{\left(0i\right)}$ *being **R** with its i*^*th*^ *row and column deleted;* ${\bar{R}}_{12}^{\left(0i\right)}$ *being the matrix* $\bar{R}$ *with its first and* (*i* + 1)^*th*^ *columns deleted, and only its first and* (*i* + 1)^*th*^ *rows kept; and* ${\bar{\mu }}_{2.1}^{\left(0i\right)}={\bar{R}}_{12}^{\left(0i\right)}{\left[R_{11}^{\left(0i\right)}\right]}^{-1}a_{1}^{\left(0i\right)}$.

The following corollary gives the first two moments of a general right truncated SGT vector $Z∼{\text{TSGT}}_{p}\left(\mu ,\Omega ,\lambda ,\nu ,a\right)$ with *ν* = (*ν*, *ν*_0_)^⊤^.

**Corollary 2** *Let* $Z∼{\text{TSGT}}_{p}\left(\mu ,\Omega ,\lambda ,\nu ,a\right)$ *with **ν*** = (*ν*, *ν*_0_)^⊤^. *Then*,

$$\begin{array}{ccc} \text{E}\{Z\} & = & \mu +\Lambda \text{E}\{X\} \end{array}$$ (25)

$$\begin{array}{ccc} \text{E}\{ZZ^{⊤}\} & = & \mu \mu^{⊤}+\Lambda \text{E}\left\{X\right\}\mu^{⊤}+\mu \text{E}\left\{X^{⊤}\right\}\Lambda +\Lambda \text{E}\left\{XX^{⊤}\right\}\Lambda \end{array}$$ (26)

*where* $\Lambda =\sqrt{\nu_{0}/\nu }\;\text{diag}\left(\omega_{1},\ldots ,\omega_{p}\right)$, $\omega_{i}^{2}$ *is the i*^*th*^ *diagonal element of* **Ω**, $X∼{\text{TST}}_{p}\left(0,R,\lambda^{*},\nu ,a^{*}\right)$, **R** *is the correlation matrix from* **Ω**, $\lambda^{*}=\sqrt{\nu_{0}/\nu }\;R^{-1/2}\Lambda^{-1}\Omega^{1/2}\lambda$, a* = Λ^−1^(**a** − ***μ***) *and* E{**X**} *and* E{**XX**^⊤^} *are available from Proposition 2*.

When *ν* → ∞, the truncated multivariate SGT family has the truncated multivariate skew normal family as a limiting case (see S5 Appendix for a definition and formulas for computing the first two moments).

## Skew generalized t-link mixed binomial model

This section defines the skew generalized t-link model (SGTLM) and describes an Expectation-Maximization (EM) algorithm [39] accelerated using parameter expansion [27] for likelihood inference. Empirical Bayes estimators of random effects and weights are also obtained for cluster specific inference.

### Model specification and marginal log-likelihood

The skew generalized t-link GLMM (SGTLM) considered in this work is defined as:

$$\begin{array}{ccc} Y_{ij}=I_{\left(0,\infty \right)}\left(Z_{ij}\right);\;Z_{i} & \overset{ind}{∼} & {\text{SGT}}_{n_{i}}(\eta_{i}-c{\tilde{U}}_{1}\upsilon_{0}\delta_{\varepsilon }J_{n_{i}},\Omega_{\varepsilon_{i}},\delta_{\varepsilon }J_{n_{i}},\nu )\\ b_{i} & \overset{ind}{∼} & {\text{ST}}_{q}\left(-c{\tilde{U}}_{1}\delta ,D,\lambda ,\nu \right) \end{array}$$ (27)

where *Y*_*ij*_ is the binary outcome of the *j*^*th*^ measurement (*j* = 1, 2, ⋯, *n*_*i*_) on the *i*^*th*^ cluster (*i* = 1, 2, ⋯, *n*), **Z**_*i*_ is a latent continuous outcome which determines the observable $Y_{i}={\left(Y_{i1},\ldots ,Y_{in_{i}}\right)}^{⊤}$, and **b**_*i*_ is a vector of *q* random effects associated to the cluster *i*. In Eq (27), ***η***_*i*_ = **X**_*i*_ *β* + **W**_*i*_ **b**_*i*_, ***β***, **X**_*i*_ and **W**_*i*_ are as in Eq (1); $c=\sqrt{2/\pi }$, ${\tilde{U}}_{1}={\left(\frac{\nu }{2}\right)}^{1/2}\frac{\Gamma (\frac{\nu -1}{2})}{\Gamma (\nu /2)}$, $\delta_{\varepsilon }\in R$, $J_{n_{i}}$ is the *n*_*i*_−*vector* of all ones, $\Omega_{\varepsilon_{i}}=I_{n_{i}}+\delta_{\varepsilon }^{2}J_{n_{i}}J_{n_{i}}^{⊤}$, $\nu ={\left(\nu ,\nu \upsilon_{0}^{2}\right)}^{⊤}$ with *υ*_0_ > 0 and *ν* > 2; $D=\bar{D}+\delta \delta^{⊤}$ and $\lambda ={\left(1+\delta^{⊤}{\bar{D}}^{-1}\delta \right)}^{1/2}D^{-1/2}\delta$ with $\delta \in R^{p}$ and $\bar{D}$ a *q* × *q* positive define matrix.

In the SGTLM, the distribution of a single latent outcome *Z*_*ij*_ is $\text{SGT}(\eta_{ij}-c{\tilde{U}}_{1}\upsilon_{0}\delta_{\varepsilon },\omega_{\varepsilon }^{2},\delta_{\varepsilon },\nu )$ where $\omega_{\varepsilon }^{2}=1+\delta_{\varepsilon }^{2}$ and $\text{SGT}(\mu ,\omega^{2},\lambda ,\nu )$ denotes a univariate SGT distribution with location *μ*, scale *ω*^2^, shape **λ** and degrees of freedom *ν*. Therefore, on denoting *SGT*(⋅|***μ***, *ω*^2^, *δ*, ***ν***) the cdf of a scalar SGT distribution $\text{SGT}(\mu ,\omega^{2},\delta ,\nu )$, the success probability of an outcome *Y*_*ij*_ given the random effects **b**_*i*_ is $P\left(Y_{ij}=1|b_{i}\right)=SGT\left(0|\eta_{ij}-c{\tilde{U}}_{1}\upsilon_{0}\delta_{\varepsilon },\omega^{2},\delta_{\varepsilon },\nu \right)$. Unlike in the common probit model (PM) (see Eq (1)), for a given cluster *i*, the *n*_*i*_ latent outcomes *Z*_*ij*_ are not independent given the random effects **b**_*i*_. Indeed, on using Eq (4) and setting ${\tilde{U}}_{2}=\frac{\nu }{\nu -2}$, the variance-covariance matrix of **Z**_*i*_ given **b**_*i*_ is

$$\begin{array}{c} \Sigma_{\varepsilon_{i}}=\upsilon_{0}^{2}[{\tilde{U}}_{2}I_{n_{i}}+\left({\tilde{U}}_{2}-c{\tilde{U}}_{1}^{2}\right)\delta_{\varepsilon }^{2}J_{n_{i}}J_{n_{i}}^{⊤}] \end{array}$$ (28)

so that the correlation coefficient between two elements *Z*_*ij*_ and *Z*_*ik*_ of **Z**_*i*_ with *k* ≠ *j* is $\rho_{0}=\frac{\left({\tilde{U}}_{2}-c{\tilde{U}}_{1}^{2}\right)\delta_{\varepsilon }^{2}}{{\tilde{U}}_{2}+\left({\tilde{U}}_{2}-c{\tilde{U}}_{1}^{2}\right)\delta_{\varepsilon }^{2}}$. Thus, conditional on random effects, the *n*_*i*_ latent outcomes in **Z**_*i*_ are uncorrelated only when *δ*_*ε*_ = 0, *i.e*. a skewed link function implies correlated latent outcomes within a cluster *i*.

The positive constant *υ*_0_ in the SGTLM controls the scale of the latent variable *Z*_*ij*_ and thus the scale of the model link function. Indeed, from Eq (28), the conditional variance of *Z*_*ij*_ is $\text{V}ar\left\{Z_{ij}|b_{i}\right\}=\upsilon_{0}^{2}[{\tilde{U}}_{2}+({\tilde{U}}_{2}-c^{2}{\tilde{U}}_{1}^{2})\delta_{\varepsilon }^{2}]$. Setting *υ*_0_ = 1 would yield a skew t-link model (*i.e*. ***ν*** = (*ν*, *ν*)^⊤^). However, to make fixed effects in the SGTLM comparable with fixed effects in the common probit model (PM) characterized by a link function with a unit scale (*i.e*. *Var*{*Z*_*ij*_|**b**_*i*_} = 1), we have set

$$\begin{array}{c} \upsilon_{0}={\left[{\tilde{U}}_{2}+\left({\tilde{U}}_{2}-c^{2}{{\tilde{U}}_{1}}^{2}\right)\delta_{\varepsilon }^{2}\right]}^{-1/2}. \end{array}$$ (29)

The application of Eq (3) to **b**_*i*_ shows that, as in the PM, random effects in the SGTLM have null mean vector E{**b**_*i*_} = 0. Using Eq (4), the variance-covariance matrix of random effects is given by

$$\begin{array}{c} \Sigma_{b}={\tilde{U}}_{2}\bar{D}+\left({\tilde{U}}_{2}-c^{2}{\tilde{U}}_{1}^{2}\right)\delta \delta^{⊤}. \end{array}$$ (30)

When *δ*_*ε*_ = 0 and ***δ*** = **0**, the SGTLM is reduced to the t-link model in [24] except *υ*_0_ = 1 therein. As *ν* → ∞ (so that *U*_*i*_ = 1), the STGLM has as limiting case the mixed skew-probit model (SPM) which reduces to the PM for *δ*_*ε*_ = 0 and ***δ*** = **0**.

By Eq (2), the SGTLM has the stochastic representation

$$\begin{array}{ccc} Y_{ij} & = & I_{\left(0,\infty \right)}\left(Z_{ij}\right)\;\;\;\;\;\\ Z_{i}|b_{i},U_{i}=u_{i},V_{i}=v_{i} & \overset{ind}{∼} & N_{n_{i}}(\eta_{i}+[v_{i}u_{i}^{-1/2}-c{\tilde{U}}_{1}]\upsilon_{0}\delta_{\varepsilon }J_{n_{i}},\upsilon_{0}^{2}u_{i}^{-1}I_{n_{i}})\\ b_{i}|U_{i}=u_{i},V_{i}=v_{i} & \overset{ind}{∼} & N_{q}([v_{i}u_{i}^{-1/2}-c{\tilde{U}}_{1}]\delta ,u_{i}^{-1}\bar{D})\\ \;\;V_{i} & \overset{ind}{∼} & \text{HN}(0,1)\;\;\text{and}\\ \;U_{i} & \overset{ind}{∼} & Gamma(\nu /2,\nu /2) \end{array}$$ (31)

where *U*_*i*_ and *V*_*i*_ are independent. In this representation of the SGTLM in terms of more common distributions, the *n*_*i*_ binary outcomes *Y*_*ij*_ of a cluster *i* are independent given the random effects **b**_*i*_, the scale mixing variable *U*_*i*_ and the half normal variable *V*_*i*_. Note that given *U*_*i*_ and *V*_*i*_, **Z**_*i*_|**b**_*i*_ and **b**_*i*_ are normally distributed and share the same *U*_*i*_ and *V*_*i*_. As a result, the joint distribution of **Z**_*i*_ and **b**_*i*_ belongs to the class of SGT distributions. From the stochastic representation in Eq (31), we obtain the unconditional distributions of **Z**_*i*_ and $Y_{i}={\left(Y_{i1},\ldots ,Y_{in_{i}}\right)}^{⊤}$ as follows.

**Proposition 3 (see** S6 Appendix **for a proof)**. *Let us consider the latent vector* **Z**_*i*_ *and the binary variable Y*_*ij*_ *in* Eq (27) *and define* $\mu_{i}=X_{i}\beta -c{\tilde{U}}_{1}\Delta_{i}$ *with* $\Omega_{i}={\bar{\Omega }}_{i}+\Delta_{i}\Delta_{i}^{⊤}$, ${\bar{\Omega }}_{i}=\upsilon_{0}^{2}I_{n_{i}}+W_{i}\bar{D}W_{i}^{⊤}$, $\Delta_{i}=\upsilon_{0}\delta_{\varepsilon }J_{n_{i}}+W_{i}\delta$, *and the related shape parameter* $\lambda_{i}=\Omega_{i}^{-1/2}\Delta_{i}{\left(1+\Delta_{i}^{⊤}{\bar{\Omega }}_{i}^{-1}\Delta_{i}\right)}^{1/2}$. *Then* $Z_{i}∼{\text{ST}}_{n_{i}}\left(\mu_{i},\Omega_{i},\lambda_{i},\nu \right)$. *Furthermore, the vector of binary outcomes* **Y**_*i*_ *has a multivariate Bernoulli distribution with joint probability mass function*,

$$\begin{array}{ccc} f_{Y}\left(y_{i}|\beta ,\delta_{\varepsilon },\delta ,\bar{D},\nu \right) & = & ST_{n_{i}}(0|A_{i}\mu_{i},A_{i}\Omega_{i}A_{i},A_{i}\lambda_{i},\nu ) \end{array}$$ (32)

*and Y*_*ij*_ *has a Bernoulli distribution with success probability* $P\left(Y_{ij}=1\right)=ST(\mu_{ij}|0,\omega_{ij}^{2},-\lambda_{ij},\nu )$ *and probability mass function*,

$$\begin{array}{ccc} f_{Y}\left(y_{ij}|\beta ,\delta_{\varepsilon },\delta ,\bar{D},\nu \right) & = & ST(0|A_{ij}\mu_{ij},\omega_{ij}^{2},A_{ij}\lambda_{ij},\nu ) \end{array}$$ (33)

*where* $A_{i}=diag\left(A_{i1},\ldots ,A_{in_{i}}\right)$, *A*_*ij*_ = 1 − 2*y*_*ij*_, $\omega_{ij}^{2}$ *is the j*^*th*^ *diagonal element of* **Ω**_*i*_, $\lambda_{ij}=\Delta_{ij}/{\bar{\omega }}_{ij}$, *and* ${\bar{\omega }}_{ij}^{2}=\omega_{ij}^{2}-\Delta_{ij}^{2}$ *with* Δ_*ij*_ *and μ*_*ij*_ *the j*^*th*^ *elements of* **Δ**_*i*_ *and **μ***_*i*_ *respectively*.

Eq (32) conveniently expresses for a value **y**_*i*_ of **Y**_*i*_, the likelihood as a cumulative probability of a ST distribution whose location, scale and shape parameters depend on **y**_*i*_, using the identities *P*(*Z*_*ij*_ > *z*_*ij*_) = *P*(−*Z*_*ij*_ < −*z*_*ij*_) and *sign*(*Z*_*ij*_) = 2*Y*_*ij*_ − 1 where *sign*(⋅) returns the sign of its argument. On using Eq (4) on the distribution of **Z**_*i*_ given in *Proposition 3*, the variance-covariance matrix of the outcomes at the latent scale is

$$\begin{array}{c} \Sigma_{i}=\Sigma_{\varepsilon_{i}}+W_{i}\Sigma_{b}W_{i}^{⊤}+\upsilon_{0}\delta_{\varepsilon }\left({\tilde{U}}_{2}-c{\tilde{U}}_{1}^{2}\right)[J_{n_{i}}\delta^{⊤}W_{i}^{⊤}+W_{i}\delta J_{n_{i}}^{⊤}]. \end{array}$$ (34)

Thus, in a model with a cluster-specific random intercept (*q* = 1) with ***δ*** = 0 and $\bar{D}={\bar{\sigma }}_{b}^{2}$, the latent intra-class correlation coefficient (the proportion of variance explained by clustering at latent scale) is given by

$$\begin{array}{c} \rho =\frac{{\tilde{U}}_{2}{\bar{\sigma }}_{b}^{2}+\left({\tilde{U}}_{2}-c{\tilde{U}}_{1}^{2}\right)\upsilon_{0}^{2}\delta_{\varepsilon }^{2}}{{\tilde{U}}_{2}\left(\upsilon_{0}^{2}+{\bar{\sigma }}_{b}^{2}\right)+\left({\tilde{U}}_{2}-c{\tilde{U}}_{1}^{2}\right)\upsilon_{0}^{2}\delta_{\varepsilon }^{2}}. \end{array}$$ (35)

The joint distribution of **Z**_*i*_ and **b**_*i*_ (*i.e*. ${\left(Z_{i}^{⊤},b_{i}^{⊤}\right)}^{⊤}$) is ${\text{ST}}_{n_{i}+q}({\tilde{\mu }}_{i},{\tilde{\Omega }}_{i},{\tilde{\lambda }}_{i},\nu )$ where ${\tilde{\mu }}_{i}=(\begin{array}{c} X_{i}\beta -c{\tilde{U}}_{1}\Delta_{i}\\ -c{\tilde{U}}_{1}\delta \end{array})$, ${\tilde{\Omega }}_{i}={\bar{\tilde{\Omega }}}_{i}+{\tilde{\delta }}_{i}{\tilde{\delta }}_{i}^{⊤}$, ${\bar{\tilde{\Omega }}}_{i}=(\begin{array}{cc} {\bar{\Omega }}_{i} & W_{i}\bar{D}\\ \bar{D}W_{i}^{⊤} & \bar{D} \end{array})$, ${\tilde{\delta }}_{i}=(\begin{array}{c} \Delta_{i}\\ \delta \end{array})$ and ${\tilde{\lambda }}_{i}={\tilde{\Omega }}_{i}^{-1/2}{\tilde{\delta }}_{i}{\left(1+{\tilde{\delta }}_{i}^{⊤}{\bar{\tilde{\Omega }}}_{i}^{-1}{\tilde{\delta }}_{i}\right)}^{1/2}$. Thus, for *j* = 1, 2, ⋯, *n*_*i*_ and *k* = 1, 2, ⋯, *q*, the correlation between *Z*_*ij*_ and *b*_*k*_ is $\rho_{ijk}=\frac{\sigma_{ijk}}{\sigma_{ij}\sigma_{k}}$ with $\sigma_{k}^{2}={\tilde{U}}_{2}{\bar{D}}_{k}^{2}+\left({\tilde{U}}_{2}-c^{2}{\tilde{U}}_{1}^{2}\right)\delta_{k}^{2}$ the variance of *b*_*k*_, $\sigma_{ij}^{2}={\tilde{U}}_{2}\left(\upsilon_{0}+W_{ij}\bar{D}W_{ij}^{⊤}\right)+\left({\tilde{U}}_{2}-c^{2}{\tilde{U}}_{1}^{2}\right){\left(\upsilon_{0}\delta_{\varepsilon }+W_{ij}\delta \right)}^{2}$ the variance of *Z*_*ij*_ and $\sigma_{ijk}={\tilde{U}}_{2}W_{ij}{\bar{D}}_{k}+\left({\tilde{U}}_{2}-c^{2}{\tilde{U}}_{1}^{2}\right)\left(\upsilon_{0}\delta_{\varepsilon }+W_{ij}\delta \right)\delta_{k}$ is the covariance between *Z*_*ij*_ and *b*_*k*_, **W**_*ij*_ is the *j*^*th*^ row of **W**_*i*_ and ${\bar{D}}_{k}$ is the *k*^*th*^ column of $\bar{D}$, ${\bar{D}}_{k}^{2}$ is the *k*^*th*^ diagonal element of $\bar{D}$ and *δ*_*k*_ is the *k*^*th*^ element of ***δ***.

The parameters of the SGTLM include ***β***, *δ*_*ε*_, ***δ***, $vech(\bar{D})$ and *ν* where the *vech*(⋅) operator returns the lower triangle elements of its matrix argument. In order to avoid non-regular likelihood problems occurring in models based on the Student distribution and its extensions (in particular when *ν* is close to zero) [40], we follow some recent related works [24, 41] and first consider *ν* as known, focusing on $\theta ={\left(\beta^{⊤},\delta_{\varepsilon },\delta^{⊤},{vech(\bar{D})}^{⊤}\right)}^{⊤}$. Classical inference on ***θ*** is based on the marginal likelihood of the observed data **y**. Using *Proposition 3*, the joint marginal log-likelihood of *n* independent clusters **y**_*i*_ (*i* = 1, 2, ⋯, *n*) is:

$$\begin{array}{c} \ell \left(\theta |y\right)=\sum_{i=1}^{n}\text{log}\;f_{i}\left(y_{i}|\theta \right) \end{array}$$ (36)

From Eq (36), an optimization routine like the R function *optim* can be used for inference on ***θ***. We however develop an EM algorithm to circumvent the *n*_*i*_-dimensional integral in Eq (32) when estimating ***θ***.

### Model identifiability

Estimations in the skew generalized t-link model (SGTLM) may produce inconsistent results which would induce unreliable and misleading conclusions, if the model is not identifiable. It is thus of great importance to check whether different points in the parameter space can be distinguished from observations **y**_*i*_. We analyse in this section the identifiability of the SGTLM and indicate when it is necessary to restrict the parameter space to ensure reliable inference from observed data. We restrict attention to the case *υ*_0_ = 1 (ignoring Eq (29)) since *υ*_0_ is an artificial device only included to ensure a unit variance in the conditional link function as in the traditional probit mixed model.

Although not sufficient, the identifiability of the SGTLM requires both the marginal random effects distribution and the conditional model given random effects to be identifiable. The identifiability of the random effects distribution follows from the identifiability of multivariate skew t distributions. We survey the identifiability of the conditional model before turning to the marginal model.

• *Conditional identifiability*

Conditional on the random effects **b**_*i*_ and for fixed degrees of freedom parameter *ν*, the identifiability of the SGTLM reduces to the identifiability of the fixed effects skew-probit model. This follows because the skew t inverse link function is an average of the skew-probit inverse link function with respect to the gamma mixing distribution. The identifiability of the skew-probit model with one covariate has been recently shown to depend on the nature (binary/continuous) of the covariate in the model [42]. Indeed, the fixed effect skew-probit model is not identifiable in the absence of any covariate (*i.e*. each **X**_*i*_ is a column of *n*_*i*_ ones) [42] (page 1624, Proposition 2.1) or in the presence of a binary covariate [42] (page 1626, Proposition 2.2). On the other hand, the fixed effect skew-probit model is identifiable when the covariate is continuous [42] (page 1627, Proposition 2.3). Extension to the case of multiple covariates is straightforwardly obtained by requiring the covariate matrix $X={\left(X_{1}^{⊤},X_{2}^{⊤},\ldots ,X_{n}^{⊤}\right)}^{⊤}$ to be of full column rank as in the classical linear regression model context. Whenever binary covariates are considered or no covariate is considered, we advocate to set *δ*_*ϵ*_ = 0 so that the conditional model reduces to a classical probit model.

• *Marginal identifiability*

From *Proposition 3*, it appears that when *υ*_0_ = 1 the paramaters *δ*_*ε*_ and ***δ*** enter the marginal distribution of **Y**_*i*_ only through the marginal working shape $\Delta_{i}=\delta_{\varepsilon }J_{n_{i}}+W_{i}\delta$ whose *j*^*th*^ element can be written $\Delta_{ij}=\delta_{\epsilon }+W_{ij}^{⊤}\delta$. As a result, caution is required when learning the model parameters from some realizations **y**_*i*_ of **Y**_*i*_. Indeed, if the model includes a random intercept term, *i.e*.**W**_*ij*_ has the form $W_{ij}={\left(1,W_{1ij}^{⊤}\right)}^{⊤}$ where $W_{1ij}\;\in \;R^{q-1}$, we can partition the random effects working shape as $\delta ={\left(\delta_{0},\delta_{1}^{⊤}\right)}^{⊤}$ with $\delta_{1}\;\in \;R^{q-1}$ so that the *j*^*th*^ element of the marginal working shape reads $\Delta_{ij}=\delta_{\epsilon }+\delta_{0}+W_{1ij}^{⊤}\delta_{1}$. Therefore, only the sum *δ*_*ϵ*_ + *δ*_0_ could be estimated and it would not be possible to distinguish in the observed skewness the part due to the random intercept from the part due to the conditional link function. This confounding issues may actually be avoided by considering more complex models based for instance on the fundamental skew distributions [43]. Recall that skewness is introduced in the SGTLM through a hidden standard half normal variable, namely *V*_*i*_. As opposed to the unique standard half normal variable *V*_*i*_ used for both **Z**_*i*_ and **b**_*i*_ in the SGTLM, the use of two different standard half normal hidden variables for **Z**_*i*_ and **b**_*i*_ [44] (page 667 eq 5-6) or two different standard half multivariate normal hidden vectors [45] (page 420 eq 2.2) remove the confounding problem.

Fortunately, the non identifiability of the skewness of conditional link function and random intercept does not affect the success probability of the response since this only depends on **Δ**_*i*_, but not on the individual values of *δ*_*ϵ*_ and *δ*_0_. However, since the conditional link scale depends on *δ*_*ϵ*_ through *υ*_0_, the confounding problem affects the scale and thus the interpretation of fixed effects. Moreover, inference on the random intercept is affected since the random intercept variance and skewness depend on *δ*_0_. For example, *δ*_*ϵ*_ + *δ*_0_ = 0 only indicates null marginal skewness, and in no way absence of link function and random intercept skewness which could be equally strong but of opposite signs. Thus, only a lower bound can be given to the random intercept variance: ${\tilde{U}}_{2}{\bar{D}}_{11}+\left({\tilde{U}}_{2}-c^{2}{\tilde{U}}_{1}^{2}\right)\delta_{0}^{2}\geq {\tilde{U}}_{2}{\bar{D}}_{11}$ where ${\bar{D}}_{11}$ is the first diagonal element of $\bar{D}$. To rule out this peculiar situation where the model is not marginally fully identifiable, some previous works on skew normal/skew t distributions have considered the restriction *δ*_*ϵ*_ = 0 (regardless of the presence of a random intercep term) for instance in the context of linear mixed effects models [30, 46, 47] (page 1492 eq 2, page 4100 eq 4, and page 309 eq 3.2 respectively), multivariate measurement error models [48] (page 35, Eq 4.11) and non linear mixed effect models [49] (page 7 eq 10), but no argument was given to support this choice.

In the very common situation where the mixed model includes a random intercept term, prior information on the shape of the link function and/or the random intercept is required to place a meaningful restriction on the parameter space by setting for instance *δ*_*ϵ*_ = 0 or *δ*_0_ = 0 or *δ*_*ϵ*_ = *δ*_0_. In the absence of such information, we advocate to consider the restriction *δ*_0_ = 0 because the success probability of a response may exibit skewness, irrespective of the presence of random effects. This restriction thus allows to recover a fixed effects skew generalized t-link model when no random effect is considered. Overall, the restriction *δ*_0_ = 0 simply expresses the unability of the SGTLM to capture any additional skewness structure from the data through the inclusion of only random intercept. For completeness, we develop in the next section an estimation procedure for the full model in Eq (27), since the introduction of any equality restriction on *δ*_*ϵ*_ and *δ*_0_ can be straightforwardly reduced to *δ*_0_ = 0.

The two restrictions discussed in this section are related to the structure of the quantity **Δ**_*i*_ and are required only for some specific data structures (models including a binary covariate or models with a random intercept only). However, even when a restriction is required on *δ*_*ε*_ or the first element of ***δ***, the quantity **Δ**_*i*_ itself can remain unrestricted. When a restriction is required, it forces the skewness from a data to be summarized either by *δ*_*ε*_ or elements of ***δ***. Overall, the SGT-link function is always allowed to be skewed (unconditional link). But some designs do not allow to distinguish skewness in the conditional link function from skewness in the distribution of random effects.

### Maximum likelihood inference

#### Estimation via the EM algorithm

The choice of the value of *υ*_0_ in Eq (29) is in line with one of our purposes: rescale fixed effects so that they have the same interpretation as in the mixed probit model. There is no need to define *υ*_0_ depending on situations, because in our proposal, *υ*_0_ is fixed. However, since *υ*_0_ is simply a scaling factor, it may be given any positive value during estimation, as long as the estimates are rescaled after the convergence of the estimation procedure so that *υ*_0_ is finally given by Eq (29). Indeed, because routines are basically written for the skew t distributions, we used *υ*_0_ = 1 in the EM algorithm and rescaled the estimates at the end of the procedure. Let us consider the complete data $y_{com_{i}}=\left\{y_{i},z_{i},b_{i},u_{i},v_{i}\right\}$. Because **y**_*i*_ only retains the signs of elements of **z**_*i*_, the joint density of **y**_*i*_ and **z**_*i*_ is $f_{YZ}\left(y_{i},z_{i}\right)=f_{Z}\left(z_{i}\right)\times I_{A_{i}}\left(z_{i}\right)$ where $A_{i}=A_{i1}\times \ldots \times A_{in_{i}}$ with $A_{ij}=\left(-\infty ,0\right]$ if *y*_*ij*_ = 0 and $A_{ij}=\left(0,\infty \right)$ if *y*_*ij*_ = 1. The density of $y_{com_{i}}$ is thus $f_{y_{com}}\left(y_{com_{i}}\right)=f_{Z,b,U,V}\left(z_{i},b_{i},u_{i},v_{i}\right)\times I_{A_{i}}\left(z_{i}\right)$. Hence by Bayes’s rule and in light of Eq (27) with *υ*_0_ = 1, the density of $y_{com_{i}}$ is:

$$\begin{array}{ccc} f_{y_{com}}\left(y_{com_{i}}\right) & = & f_{z|b,U,V}\left(z_{i}|b_{i},u_{i},v_{i}\right)\times f_{b|U,V}\left(b_{i}|u_{i},v_{i}\right)\times f_{U}\left(u_{i}\right)\times f_{V}\left(v_{i}\right)\times I_{A_{i}}\left(z_{i}\right)\\ & = & \phi_{n_{i}}(z_{i}|\eta_{i}+(v_{i}u_{i}^{-1/2}-c{\tilde{U}}_{1})\delta_{\varepsilon }J_{n_{i}},u_{i}^{-1}I_{n_{i}})\\ & & \times \;\phi_{q}(b_{i}|(v_{i}u_{i}^{-1/2}-c{\tilde{U}}_{1})\delta ,u_{i}^{-1}\bar{D})\times f_{G}\left(u_{i}|\nu /2,\nu /2\right)\\ & & \times \;f_{V}\left(v_{i}\right)\times I_{A_{i}}\left(z_{i}\right)\;\;\;\; \end{array}$$ (37)

where *f*_*V*_(*v*_*i*_) = 2*ϕ*(*v*_*i*_) *I*_(0,∞)_(*v*_*i*_) and *f*_*G*_(⋅|*ν*/2, *ν*/2) is given in Eq (9). By Eq (37) and on setting $N=\sum_{i=1}^{n}n_{i}$ and $C_{\ell }=-\frac{N+n(q+1)}{2}\text{log}\;\left(2\pi \right)+n\;\text{log}\;2$, the complete data log-likelihood is:

$$\begin{array}{ccc} \ell (\theta |y_{com}) & = & C_{\ell }-\frac{1}{2}\sum_{i=1}^{n}[\beta^{⊤}X_{i}^{⊤}X_{i}\beta u_{i}-2\beta^{⊤}X_{i}^{⊤}\gamma_{i}u_{i}+\gamma_{i}^{⊤}\gamma_{i}u_{i}]-\frac{n}{2}\;\text{log}\;\left|\bar{D}\right|\\ & & +\frac{1}{2}\sum_{i=1}^{n}n_{i}\;\text{log}\;u_{i}+\frac{q}{2}\sum_{i=1}^{n}\;\text{log}\;u_{i}-\frac{1}{2}\sum_{i=1}^{n}tr({\bar{D}}^{-1}\delta \delta^{⊤}){\left(v_{i}-c{\tilde{U}}_{1}u_{i}^{1/2}\right)}^{2}\\ & & -\frac{1}{2}\sum_{i=1}^{n}tr({\bar{D}}^{-1}[u_{i}b_{i}b_{i}^{⊤}-(v_{i}-c{\tilde{U}}_{1}u_{i}^{1/2})(u_{i}^{1/2}b_{i}\delta^{⊤}+\delta u_{i}^{1/2}b_{i}^{⊤})])\\ & & +\sum_{i=1}^{n}\;\text{log}\;f_{G}\left(u_{i}|\nu /2,\nu /2\right)-\frac{1}{2}\sum_{i=1}^{n}v_{i}^{2}+\sum_{i=1}^{n}\;\text{log}\;I_{A_{i}}\left(z_{i}\right) \end{array}$$ (38)

where $\gamma_{i}=z_{i}-W_{i}b_{i}-\delta_{\varepsilon }(v_{i}u_{i}^{-1/2}-c{\tilde{U}}_{1})J_{n_{i}}$, and *tr*(⋅) is the trace operator. Let ${\hat{\theta }}^{\left(k\right)}$ the estimate of ***θ*** at the *k*^*th*^ EM iteration. The E-step of the (*k* + 1)^*th*^ iteration finds the expectation $Q(\cdot |{\hat{\theta }}^{\left(k\right)})$ of *ℓ*(⋅|**y**_*com*_) given the observed data **y** and the current parameter estimate ${\hat{\theta }}^{\left(k\right)}={\left({\hat{\beta }}^{(k)⊤},{\hat{\delta }}_{\varepsilon }^{\left(k\right)},{\hat{\delta }}^{(k)⊤},{vech({\hat{\bar{D}}}^{\left(k\right)})}^{⊤}\right)}^{⊤}$:

$$\begin{array}{ccc} Q(\theta |{\hat{\theta }}^{\left(k\right)}) & = & C_{\ell }-\frac{1}{2}\sum_{i=1}^{n}[\beta^{⊤}X_{i}^{⊤}X_{i}\beta {\hat{u_{2}}}_{i}^{\left(k\right)}-2\beta^{⊤}X_{i}^{⊤}{\hat{u_{2}\gamma }}_{i}+tr({\hat{u_{2}\gamma_{2}}}_{i}^{\left(k\right)})]\\ & & -\frac{n}{2}\;\text{log}\;\left|\bar{D}\right|-\frac{1}{2}\sum_{i=1}^{n}tr({\bar{D}}^{-1}[{\hat{u_{2}b_{2}}}_{i}^{\left(k\right)}+(c{\tilde{U}}_{1}{\hat{u_{2}b}}_{i}^{\left(k\right)}-{\hat{vub}}_{i}^{\left(k\right)})\delta^{⊤}\\ & & \;+\;\delta {\left(c{\tilde{U}}_{1}{\hat{u_{2}b}}_{i}^{\left(k\right)}-{\hat{vub}}_{i}^{\left(k\right)}\right)}^{⊤}])+\;\frac{1}{2}\sum_{i=1}^{n}(n_{i}+q+\nu -2){\hat{\;\text{log}\;u_{2}}}_{i}^{\left(k\right)}\\ & & -\frac{1}{2}\sum_{i=1}^{n}tr({\bar{D}}^{-1}\delta \delta^{⊤})({\hat{v_{2}}}_{i}^{\left(k\right)}-2c{\tilde{U}}_{1}{\hat{vu}}_{i}^{\left(k\right)}+c^{2}{\tilde{U}}_{1}^{2}{\hat{u_{2}}}_{i}^{\left(k\right)})\\ & & -\frac{\nu }{2}\sum_{i=1}^{n}{\hat{u_{2}}}_{i}^{\left(k\right)}-\frac{1}{2}\sum_{i=1}^{n}{\hat{v_{2}}}_{i}^{\left(k\right)}+\frac{n\nu }{2}\;\text{log}\;(\frac{\nu }{2})-n\;\text{log}\;\Gamma (\frac{\nu }{2}) \end{array}$$ (39)

where ${\hat{u_{2}\gamma }}_{i}^{\left(k\right)}={\hat{u_{2}z}}_{i}^{\left(k\right)}-W_{i}{\hat{u_{2}b}}_{i}^{\left(k\right)}-\delta_{\varepsilon }({\hat{vu}}_{i}^{\left(k\right)}-c{\tilde{U}}_{1}{\hat{u_{2}}}_{i}^{\left(k\right)})J_{n_{i}}$ and

$$\begin{array}{ccc} {\hat{u_{2}\gamma_{2}}}_{i}^{\left(k\right)} & = & {\hat{u_{2}z_{2}}}_{i}^{\left(k\right)}+W_{i}{\hat{u_{2}b_{2}}}_{i}^{\left(k\right)}W_{i}^{⊤}+\delta_{\varepsilon }^{2}({\hat{v_{2}}}_{i}^{\left(k\right)}-2c{\tilde{U}}_{1}{\hat{vu}}_{i}^{\left(k\right)}+c^{2}{\tilde{U}}_{1}^{2}{\hat{u_{2}}}_{i}^{\left(k\right)})J_{n_{i}}J_{n_{i}}^{⊤}\\ & & -\delta_{\varepsilon }[({\hat{vuz}}_{i}^{\left(k\right)}-W_{i}{\hat{vub}}_{i}^{\left(k\right)})-c{\tilde{U}}_{1}({\hat{u_{2}z}}_{i}^{\left(k\right)}-W_{i}{\hat{u_{2}b}}_{i}^{\left(k\right)})]J_{n_{i}}^{⊤}\\ & & -\delta_{\varepsilon }J_{n_{i}}{\left[({\hat{vuz}}_{i}^{\left(k\right)}-W_{i}{\hat{vub}}_{i}^{\left(k\right)})-c{\tilde{U}}_{1}({\hat{u_{2}z}}_{i}^{\left(k\right)}-W_{i}{\hat{u_{2}b}}_{i}^{\left(k\right)})\right]}^{⊤}\\ & & -[W_{i}\;{\hat{u_{2}bz}}_{i}^{\left(k\right)}+{\left({\hat{u_{2}bz}}_{i}^{\left(k\right)}\right)}^{⊤}W_{i}^{⊤}] \end{array}$$

so that we have

$$\begin{array}{ccc} tr({\hat{u_{2}\gamma_{2}}}_{i}^{\left(k\right)}) & = & tr({\hat{u_{2}z_{2}}}_{i}^{\left(k\right)})+tr(W_{i}{\hat{u_{2}b_{2}}}_{i}^{\left(k\right)}W_{i}^{⊤})-2tr(W_{i}{\hat{u_{2}bz}}_{i}^{\left(k\right)})\\ & & +\;n_{i}\delta_{\varepsilon }^{2}({\hat{v_{2}}}_{i}^{\left(k\right)}-2c{\tilde{U}}_{1}{\hat{vu}}_{i}^{\left(k\right)}+c^{2}{\tilde{U}}_{1}^{2}{\hat{u_{2}}}_{i}^{\left(k\right)})\\ & & -\;2\delta_{\varepsilon }J_{n_{i}}^{⊤}[({\hat{vuz}}_{i}^{\left(k\right)}-W_{i}{\hat{vub}}_{i}^{\left(k\right)})-c{\tilde{U}}_{1}({\hat{u_{2}z}}_{i}^{\left(k\right)}-W_{i}{\hat{u_{2}b}}_{i}^{\left(k\right)})]. \end{array}$$

Note that the conditional expectation of $\text{log}\;I_{A_{i}}\left(z_{i}\right)$ is 0 since given **y**_*i*_, $I_{A_{i}}\left(z_{i}|y_{i}\right)=1$. The E-step thus reduces to the computation of the conditional expectations ${\hat{u_{2}b}}_{i}^{\left(k\right)}=\text{E}\left\{U_{i}b_{i},{\hat{\theta }}^{\left(k\right)}\right\}$, ${\hat{vub}}_{i}^{\left(k\right)}=\text{E}\left\{V_{i}U_{i}^{1/2}b_{i}|y_{i},{\hat{\theta }}^{\left(k\right)}\right\}$, ${\hat{u_{2}bz}}_{i}^{\left(k\right)}=\text{E}\left\{U_{i}b_{i}Z_{i}^{⊤}|y_{i},{\hat{\theta }}^{\left(k\right)}\right\}$, ${\hat{u_{2}b_{2}}}_{i}^{\left(k\right)}=\text{E}\left\{U_{i}b_{i}b_{i}^{⊤}|y_{i},{\hat{\theta }}^{\left(k\right)}\right\}$, ${\hat{vu}}_{i}^{\left(k\right)}=\text{E}\left\{V_{i}U_{i}^{1/2}|y_{i},{\hat{\theta }}^{\left(k\right)}\right\}$, ${\hat{v_{2}}}_{i}^{\left(k\right)}=\text{E}\left\{V_{i}^{2}|y_{i},{\hat{\theta }}^{\left(k\right)}\right\}$, ${\hat{u_{2}}}_{i}^{\left(k\right)}=\text{E}\left\{U_{i}|y_{i},{\hat{\theta }}^{\left(k\right)}\right\}$, ${\hat{u_{2}z}}_{i}^{\left(k\right)}=\text{E}\left\{U_{i}Z_{i}|y_{i},{\hat{\theta }}^{\left(k\right)}\right\}$, ${\hat{vuz}}_{i}^{\left(k\right)}=\text{E}\left\{V_{i}U_{i}^{1/2}Z_{i}|y_{i},{\hat{\theta }}^{\left(k\right)}\right\}$, ${\hat{u_{2}z_{2}}}_{i}^{\left(k\right)}=\text{E}\left\{U_{i}Z_{i}Z_{i}^{⊤}|y_{i},{\hat{\theta }}^{\left(k\right)}\right\}$ and ${\hat{\;\text{log}\;u_{2}}}_{i}^{\left(k\right)}=\text{E}\left\{\;\text{log}\;U_{i}|y_{i},{\hat{\theta }}^{\left(k\right)}\right\}$. The expressions for these expectations (except ${\hat{\text{log}\;u_{2}}}_{i}^{\left(k\right)}$) are given in the following result where we have dropped the supraindex (*k*) for simplicity.

**Proposition 4 (see** S7 Appendix **for a proof)**. *Consider the random variables Y*_*ij*_, **Z**_*i*_, **b**_*i*_, *U*_*i*_, *and V*_*i*_ *as defined in* Eq (27) *with υ*_0_ = 1, *and an update* $\hat{\theta }={\left({\hat{\beta }}^{⊤},{\hat{\delta }}_{\varepsilon },{\hat{\delta }}^{⊤},{vech(\hat{\bar{D}})}^{⊤}\right)}^{⊤}$ *of the model parameter **θ***. *Let* ${\hat{\bar{\Omega }}}_{i}=I_{n_{i}}+W_{i}\hat{\bar{D}}W_{i}^{⊤}$, ${\hat{r}}_{i}=\hat{\bar{D}}W_{i}^{⊤}{\hat{\bar{\Omega }}}_{i}^{-1}$, ${\hat{\Lambda }}_{i}=(I_{q}-{\hat{r}}_{i}W_{i})\hat{\bar{D}}$, ${\hat{\Delta }}_{i}={\hat{\delta }}_{\varepsilon }J_{n_{i}}+W_{i}\hat{\delta }$, ${\hat{s}}_{i}=\hat{\delta }-{\hat{r}}_{i}{\hat{\Delta }}_{i}$, ${\hat{\mu }}_{i}=X_{i}\hat{\beta }-c{\tilde{U}}_{1}{\hat{\Delta }}_{i}$, ${\hat{M}}_{i}={\left(1+{\hat{\Delta }}_{i}^{⊤}{\hat{\bar{\Omega }}}_{i}^{-1}{\hat{\Delta }}_{i}\right)}^{-1/2}$, ${\hat{\Omega }}_{i}={\hat{\bar{\Omega }}}_{i}+{\hat{\Delta }}_{i}{\hat{\Delta }}_{i}^{⊤}$, ${\hat{\lambda }}_{i}={\hat{M}}_{i}^{-1}{\hat{\Omega }}_{i}^{-1/2}{\hat{\Delta }}_{i}$, ${Z_{0}}_{i}={\hat{\Omega }}_{i}^{-1/2}(Z_{i}-{\hat{\mu }}_{i})$. *Then*:

$$\begin{array}{ccc} {\hat{u_{2}b}}_{i} & = & {\hat{r}}_{i}({\hat{u_{2}z}}_{i}-{\hat{u_{2}}}_{i}X_{i}\hat{\beta })+({\hat{vu}}_{i}-c{\tilde{U}}_{1}{\hat{u_{2}}}_{i}){\hat{s}}_{i} \end{array}$$ (40)

$$\begin{array}{ccc} {\hat{vub}}_{i} & = & {\hat{r}}_{i}({\hat{vuz}}_{i}-{\hat{vu}}_{i}X_{i}\hat{\beta })+({\hat{v_{2}}}_{i}-c{\tilde{U}}_{1}{\hat{vu}}_{i}){\hat{s}}_{i} \end{array}$$ (41)

$$\begin{array}{ccc} {\hat{u_{2}bz}}_{i} & = & {\hat{r}}_{i}({\hat{u_{2}z_{2}}}_{i}-X_{i}\hat{\beta }\;{\hat{u_{2}z}}_{i}^{⊤})+\;{\hat{s}}_{i}{\left({\hat{vuz}}_{i}-c{\tilde{U}}_{1}{\hat{u_{2}z}}_{i}\right)}^{⊤} \end{array}$$ (42)

$$\begin{array}{ccc} {\hat{u_{2}b_{2}}}_{i} & = & {\hat{\Lambda }}_{i}+({\hat{v_{2}}}_{i}-2c{\tilde{U}}_{1}{\hat{vu}}_{i}+c^{2}{\tilde{U}}_{1}^{2}{\hat{u_{2}}}_{i}){\hat{s}}_{i}{\hat{s}}_{i}^{⊤}\\ & & +\;{\hat{r}}_{i}[{\hat{u_{2}z_{2}}}_{i}+{\hat{u_{2}}}_{i}X_{i}\hat{\beta }{\hat{\beta }}^{⊤}X_{i}^{⊤}-{\hat{u_{2}z}}_{i}{\hat{\beta }}^{⊤}X_{i}^{⊤}-X_{i}\hat{\beta }\;{\hat{u_{2}z}}_{i}^{⊤}]{\hat{r}}_{i}^{⊤}\\ & & +\;{\hat{r}}_{i}[({\hat{vuz}}_{i}-{\hat{vu}}_{i}X_{i}\hat{\beta })-c{\tilde{U}}_{1}({\hat{u_{2}z}}_{i}-{\hat{u_{2}}}_{i}X_{i}\hat{\beta })]{\hat{s}}_{i}^{⊤}\\ & & +\;{\hat{s}}_{i}{\left[({\hat{vuz}}_{i}-{\hat{vu}}_{i}X_{i}\hat{\beta })-c{\tilde{U}}_{1}({\hat{u_{2}z}}_{i}-{\hat{u_{2}}}_{i}X_{i}\hat{\beta })\right]}^{⊤}{\hat{r}}_{i}^{⊤} \end{array}$$ (43)

$$\begin{array}{ccc} {\hat{vu}}_{i} & = & {\hat{M}}_{i}({\hat{u_{2}\alpha }}_{i}+{\hat{\tau }}_{i}) \end{array}$$ (44)

$$\begin{array}{ccc} {\hat{vuz}}_{i} & = & {\hat{M}}_{i}({\hat{u_{2}\alpha z}}_{i}+{\hat{\tau z}}_{i}) \end{array}$$ (45)

$$\begin{array}{ccc} {\hat{v_{2}}}_{i} & = & {\hat{M}}_{i}^{2}(1+{\hat{\tau \alpha }}_{i}+{\hat{u_{2}\alpha_{2}}}_{i}) \end{array}$$ (46)

*where* ${\hat{u_{2}\alpha }}_{i}=\;{\hat{M}}_{i}{\hat{\Delta }}_{i}^{⊤}{\hat{\bar{\Omega }}}_{i}^{-1}({\hat{u_{2}z}}_{i}-{\hat{u_{2}}}_{i}{\hat{\mu }}_{i})$, ${\hat{\tau \alpha }}_{i}\;=\;{\hat{M}}_{i}{\hat{\Delta }}_{i}^{⊤}{\hat{\bar{\Omega }}}_{i}^{-1}({\hat{\tau z}}_{i}\;-{\hat{\tau }}_{i}{\hat{\mu }}_{i})$, ${\hat{u_{2}\alpha z}}_{i}\;=\;{\hat{M}}_{i}\;({\hat{u_{2}z_{2}}}_{i}\;-\;{\hat{u_{2}z}}_{i}{\hat{\mu }}_{i}^{⊤})\;{\hat{\bar{\Omega }}}_{i}^{-1}\;{\hat{\Delta }}_{i}$, ${\hat{u_{2}\alpha_{2}}}_{i}={\hat{M}}_{i}^{2}[{\hat{u_{2}}}_{i}{\left({\hat{\Delta }}_{i}^{⊤}{\hat{\bar{\Omega }}}_{i}^{-1}{\hat{\mu }}_{i}\right)}^{2}+{\hat{\Delta }}_{i}^{⊤}{\hat{\bar{\Omega }}}_{i}^{-1}({\hat{u_{2}z_{2}}}_{i}\;-\;2{\hat{u_{2}z}}_{i}{\hat{\mu }}_{i}^{⊤}){\hat{\bar{\Omega }}}_{i}^{-1}{\hat{\Delta }}_{i}]$, *and the expectations* ${\hat{u_{2}z}}_{i}=\text{E}\left\{U_{i}Z_{i}|y_{i},\hat{\theta }\right\}$, ${\hat{u_{2}z_{2}}}_{i}=\text{E}\left\{U_{i}Z_{i}Z_{i}^{⊤}|y_{i},\hat{\theta }\right\}$, ${\hat{\tau }}_{i}=\text{E}\left\{U_{i}^{1/2}\zeta_{1}\left(U_{i}^{1/2}\lambda^{⊤}Z_{0i}\right)|y_{i},\hat{\theta }\right\}$, ${\hat{\tau z}}_{i}=\text{E}\left\{U_{i}^{1/2}\zeta_{1}\left(U_{i}^{1/2}\lambda^{⊤}Z_{0i}\right)Z_{i}|y_{i},\hat{\theta }\right\}$ *and* ${\hat{u_{2}}}_{i}=\text{E}\left\{U_{i}|y_{i},\hat{\theta }\right\}$ *are to be evaluated directly using Corollary 1 applied to the conditional latent vector* $Z_{i}|Y_{i}=y_{i},\theta =\hat{\theta }∼{\text{TST}}_{n_{i}}({\hat{\mu }}_{i},{\hat{\Omega }}_{i},{\hat{\lambda }}_{i},\hat{\nu },A_{i})$, $A_{i}=A_{i1}\times \ldots \times A_{in_{i}}$ *with* $A_{ij}=\left(-\infty ,0\right]$ *if y*_*ij*_ = 0 *and* $A_{ij}=\left(0,\infty \right)$ *if y*_*ij*_ = 1.

The M-step jointly maximizes $Q(\cdot |{\hat{\theta }}^{\left(k\right)})$ over ***θ***. This yields the following updating expressions for ***θ***.

**Proposition 5 (see** S8 Appendix **for a proof)** *Consider an identifiable SGTLM as defined in* Eq (27) *with υ*_0_ = 1; *and an estimate* ${\hat{\theta }}^{\left(k\right)}$ *of **θ***. *Set* ${\hat{S_{1}}}_{i}^{\left(k\right)}={\hat{u_{2}z}}_{i}^{\left(k\right)}-W_{i}{\hat{u_{2}b}}_{i}^{\left(k\right)}$, ${\hat{S_{2}}}_{i}^{\left(k\right)}={\hat{vu}}_{i}^{\left(k\right)}-c{\tilde{U}}_{1}{\hat{u_{2}}}_{i}^{\left(k\right)}$, ${\hat{S_{3}}}_{i}^{\left(k\right)}={\hat{v_{2}}}_{i}^{\left(k\right)}-2c{\tilde{U}}_{1}{\hat{vu}}_{i}^{\left(k\right)}+c^{2}{\tilde{U}}_{1}^{2(k)}{\hat{u_{2}}}_{i}^{\left(k\right)}$, ${\hat{S_{4}}}_{i}^{\left(k\right)}={\hat{vuz}}_{i}^{\left(k\right)}-W_{i}{\hat{vub}}_{i}^{\left(k\right)}$, ${\hat{T}}_{1}^{\left(k\right)}={\left[\sum_{i=1}^{n}{\hat{u_{2}}}_{i}^{\left(k\right)}X_{i}^{⊤}X_{i}\right]}^{-1}$ *and* ${\hat{T}}_{2}^{\left(k\right)}=\sum_{i=1}^{n}{\hat{S_{2}}}_{i}^{\left(k\right)}J_{n_{i}}^{⊤}X_{i}$. *At EM iteration* (*k* + 1), *the updates of **β***, *δ*_*ε*_, ***δ*** *and* $\bar{D}$ *are given by*:

$$\begin{array}{ccc} {\hat{\beta }}^{\left(k+1\right)} & = & {\hat{T}}_{1}^{\left(k\right)}\sum_{i=1}^{n}X_{i}^{⊤}({\hat{S_{1}}}_{i}^{\left(k\right)}-{\hat{\delta_{\varepsilon }}}^{\left(k+1\right)}{\hat{S_{2}}}_{i}^{\left(k\right)}J_{n_{i}}) \end{array}$$ (47)

$$\begin{array}{ccc} {\hat{\delta_{\varepsilon }}}^{\left(k+1\right)} & = & \left[{\hat{T}}_{2}^{\left(k\right)}{\hat{T}}_{1}^{\left(k\right)}(\sum_{i=1}^{n}X_{i}^{⊤}{\hat{S_{1}}}_{i}^{\left(k\right)})-\sum_{i=1}^{n}J_{n_{i}}^{⊤}({\hat{S_{4}}}_{i}^{\left(k\right)}-c{\tilde{U}}_{1}{\hat{S_{1}}}_{i}^{\left(k\right)})\right]\\ & & \times {\left[{\hat{T}}_{2}^{\left(k\right)}{\hat{T}}_{1}^{\left(k\right)}{\hat{T}}_{2}^{\left(k\right)}-\sum_{i=1}^{n}n_{i}{\hat{S_{3}}}_{i}^{\left(k\right)}\right]}^{-1} \end{array}$$ (48)

$$\begin{array}{ccc} {\hat{\delta }}^{\left(k+1\right)} & = & {\left[\sum_{i=1}^{n}{\hat{S_{3}}}_{i}^{\left(k\right)}\right]}^{-1}\sum_{i=1}^{n}({\hat{vub}}_{i}^{\left(k\right)}-c{\tilde{U}}_{1}{\hat{u_{2}b}}_{i}^{\left(k\right)}) \end{array}$$ (49)

$$\begin{array}{ccc} {\hat{\bar{D}}}^{\left(k+1\right)} & = & n^{-1}[(\sum_{i=1}^{n}{\hat{u_{2}b_{2}}}_{i}^{\left(k\right)})-(\sum_{i=1}^{n}{\hat{S_{3}}}_{i}^{\left(k\right)}){\hat{\delta }}^{\left(k+1\right)}{\left({\hat{\delta }}^{\left(k+1\right)}\right)}^{⊤}]. \end{array}$$ (50)

At convergence of the EM algorithm, we obtain the estimate $\tilde{\theta }\left(\nu \right)$ of ***θ***. The corresponding estimate of the variance-covariance matrix of random effects is ${\hat{\Sigma }}_{b}={\tilde{U}}_{2}\hat{\bar{D}}+\left({\tilde{U}}_{2}-c^{2}{\tilde{U}}_{1}^{2}\right)\hat{\delta }{\hat{\delta }}^{⊤}$. More generally, when *ν* is not actually known, the M-step of the EM algorithm can be extended to include a profiled marginal log-likelihood maximization step. Indeed, at EM iteration *k*, we notice that the estimate ${\tilde{\theta }}^{\left(k\right)}\left(\nu \right)$ of ***θ*** depends on *ν* only through ${\tilde{U}}_{1}$ and the profiled marginal log-likelihood *L*_*ν*_(⋅) for *ν* can be obtained by simply substituting ${\tilde{\theta }}^{\left(k\right)}\left(\nu \right)$ for ***θ*** in Eq (36). We can thus find ${\hat{\nu }}^{\left(k+1\right)}$ using a one dimensional optimization routine (*e.g*. *optimize* in R) to maximize *L*_*ν*_(⋅). Then, the update of the parameter ***θ*** becomes ${\hat{\theta }}^{\left(k+1\right)}={\tilde{\theta }}^{\left(k\right)}\left({\hat{\nu }}^{\left(k+1\right)}\right)$. The use of the profiled marginal log-likelihood instead of a profiled version of $Q(\cdot |{\hat{\theta }}^{\left(k\right)})$ can provide substantial gain of efficiency [50] and mostly helps bypass the calculation of ${\hat{\text{log}\;u_{2}}}_{i}^{\left(k\right)}$ which does not have any known closed form.

It is worthwhile noticing however that the inclusion of a profiled marginal log-likelihood maximization step would prevent the convergence of the whole estimation procedure if the marginal log-likelihood in Eq (36) as a function of *ν* is unbounded for a particular dataset. This issue especially when *ν* is close to zero. Another challenge associated to the estimation of *ν* is time. The use of this strategy requires a very fast routine to compute cumulative probabilities of the skew t distributions. As an alternative route to estimate *ν*, we point out the model selection approach of [51] (page 893). It consists in setting a grid of feasible values of *ν* and obtaining a sequence of estimates $\tilde{\theta }\left(\nu \right)$ of ***θ***. Then, the couple *ν* and $\tilde{\theta }\left(\nu \right)$ maximizing the marginal log-like-lihood in Eq (36) is taken as the estimates of *ν* and ***θ***.

#### Accelerating EM via parameter-expansion

Besides its attractiveness and stability for handling incomplete data models, the EM algorithm sometimes experiences slow convergence, which has motivated many methods to accelerate its linear convergence speed. Among popular EM accelerators, the so-called parameter-expanded (PX) EM algorithm was proposed by [27] to speed up convergence. Let us consider a complete data model ***F***(**y**_*com*_|***θ***). The PX-EM algorithm expands ***F***(**y**_*com*_|***θ***) to a larger model ***F***_*X*_(**y**_*com*_|Θ) parameterized by $\Theta ={\left(\theta_{⋆}^{⊤},\alpha^{⊤}\right)}^{⊤}$ where ***θ***_⋆_ plays in ***F***_*X*_(**y**_*com*_|Θ) the role of ***θ*** in ***F***(**y**_*com*_|***θ***) and ***α*** is a working parameter. The use of the PX-EM algorithm requires that (1) ***α*** admits a value ***α***_0_ that preserves the original complete data model and (ii) the observed-data model is preserved by a many-to-one reduction function ***R***: Θ ↦ ***θ*** = ***R***(Θ) which allows an unambigious recovering of ***θ*** from Θ. We refer to [27] for more details. For the SGTLM, let us consider the following expanded complete data model obtained by including a working *q* × *q* scale matrix ***α*** into the linear predictor as ***η***_*i*_ = **X**_*i*_ *β*_⋆_ + **W**_*i*_ *α* **b**_*i*_:

$$\begin{array}{ccc} Y_{ij} & = & I_{\left(0,\infty \right)}\left(Z_{ij}\right)\\ Z_{i}|b_{i},U_{i}=u_{i},V_{i}=v_{i} & \overset{ind}{∼} & N_{n_{i}}(\eta_{i}+[v_{i}u_{i}^{-1/2}-c{\tilde{U}}_{1}]\delta_{\varepsilon ⋆}J_{n_{i}},u_{i}^{-1}I_{n_{i}})\\ b_{i}|U_{i}=u_{i},V_{i}=v_{i} & \overset{ind}{∼} & N_{q}([v_{i}u_{i}^{-1/2}-c{\tilde{U}}_{1}]\delta_{⋆},u_{i}^{-1}{\bar{D}}_{⋆})\\ U_{i} & \overset{ind}{∼} & Gamma(\nu_{⋆}/2,\nu_{⋆}/2)\\ V_{i} & \overset{ind}{∼} & \text{HN}(0,1) \end{array}$$

This expanded model equals the STGLM in Eq (27) when ***α*** takes the value ***α***_0_ = **I**_*q*_, and has expanded parameter $\Theta ={\left(\beta_{⋆}^{⊤},\delta_{\varepsilon ⋆},\delta_{⋆}^{⊤},{vech({\bar{D}}_{⋆})}^{⊤},{vec(\alpha )}^{⊤}\right)}^{⊤}$ where *vec* is the usual operator which stacks the columns of its matrix argument. Under this model, the marginal distribution of **Y**_*i*_ remains as given in Eq (32) with ${\bar{\Omega }}_{i}=I_{n_{i}}+W_{i}\alpha {\bar{D}}_{⋆}\alpha^{⊤}W_{i}^{⊤}$ and $\Delta_{i}=\delta_{\varepsilon }J_{n_{i}}+W_{i}\alpha \delta$ so that **Θ** reduces as $\theta ={\left(\beta_{⋆}^{⊤},\delta_{\varepsilon ⋆},\delta_{⋆}^{⊤}\alpha^{⊤},{vech(\alpha {\bar{D}}_{⋆}\alpha^{⊤})}^{⊤}\right)}^{⊤}$. As the observed-data model is preserved whatever the value of ***α***, we fix ***α*** = **I**_*q*_ at each E-step of the EM procedure. Therefore, the E-step of the PX-EM algorithm uses *Proposition 4* to obtain conditional expectations required in Eq (39) as for the classical EM algorithm. At the M-step, the estimates of ***δ***_⋆_ and ${\bar{D}}_{⋆}$ are still given by Eqs (49)–(50) respectively whereas the estimates of *δ*_*ε*⋆_, ***β***_⋆_ and *vec*(***α***) are:

$$\begin{array}{ccc} \left(\begin{array}{c} {\hat{\beta }}_{⋆}^{\left(k+1\right)}\\ vec({\hat{\alpha }}^{\left(k+1\right)}) \end{array}\right) & = & \Psi^{\left(k\right)}({ϒ}^{\left(k\right)}-{\hat{\delta }}_{\varepsilon ⋆}^{\left(k+1\right)}\xi^{\left(k\right)}) \end{array}$$ (51)

$$\begin{array}{ccc} {\hat{\delta }}_{\varepsilon ⋆}^{\left(k+1\right)} & = & \left[\xi^{(k)⊤}\Psi^{\left(k\right)}{ϒ}^{\left(k\right)}-\sum_{i=1}^{n}J_{n_{i}}^{⊤}({\hat{vuz}}_{i}^{\left(k\right)}-c{\tilde{U}}_{1}{\hat{u_{2}z}}_{i}^{\left(k\right)})\right]\\ & & \times {\left[\xi^{(k)⊤}\Psi^{\left(k\right)}\xi^{\left(k\right)}-\sum_{i=1}^{n}n_{i}{\hat{S_{3}}}_{i}^{\left(k\right)}\right]}^{-1}\;\;\; \end{array}$$ (52)

where $\xi^{\left(k\right)}=(\begin{array}{c} \sum_{i=1}^{n}{\hat{S_{2}}}_{i}^{\left(k\right)}X_{i}^{⊤}J_{n_{i}}\\ \sum_{i=1}^{n}(W_{i}^{⊤}J_{n_{i}})⊗({\hat{vub}}_{i}^{\left(k\right)}-c{\tilde{U}}_{1}{\hat{u_{2}b}}_{i}^{\left(k\right)}) \end{array})$, ${ϒ}^{\left(k\right)}=(\begin{array}{c} \sum_{i=1}^{n}X_{i}^{⊤}{\hat{u_{2}z}}_{i}^{\left(k\right)}\\ vec(\sum_{i=1}^{n}W_{i}^{⊤}{\hat{u_{2}bz}}_{i}^{⊤(k)}) \end{array})$, and $\Psi^{\left(k\right)}={\left(\begin{array}{cc} \sum_{i=1}^{n}X_{i}^{⊤}X_{i}{\hat{u_{2}}}_{i}^{\left(k\right)} & \sum_{i=1}^{n}X_{i}^{⊤}{\left({\hat{u_{2}b}}_{i}^{\left(k\right)}⊗W_{i}^{⊤}\right)}^{⊤}\\ \sum_{i=1}^{n}({\hat{u_{2}b}}_{i}^{\left(k\right)}⊗W_{i}^{⊤})X_{i} & \sum_{i=1}^{n}{\hat{u_{2}b_{2}}}_{i}^{\left(k\right)}⊗(W_{i}^{⊤}W_{i}) \end{array}\right)}^{-1}$ with ⊗ the direct product operator. Using the reduction function, the original model parameter estimates can be recovered as ${\hat{\beta }}^{\left(k+1\right)}={\hat{\beta }}_{⋆}^{\left(k+1\right)}$, ${\hat{\delta }}_{\varepsilon }^{\left(k+1\right)}={\hat{\delta }}_{\varepsilon ⋆}^{\left(k+1\right)}$, ${\hat{\delta }}^{\left(k+1\right)}={\hat{\alpha }}^{\left(k+1\right)}{\hat{\delta }}_{⋆}^{\left(k+1\right)}$ and ${\hat{\bar{D}}}^{\left(k+1\right)}={\hat{\alpha }}^{\left(k+1\right)}{\hat{\bar{D}}}_{⋆}^{\left(k+1\right)}{\hat{\alpha }}^{(k+1)⊤}$. In the neighbourhood of the ML estimate of ***θ***, the working scale estimate $\hat{\alpha }$ becomes close to ***α***_0_ = **I**_*q*_ [27] so that the advantage of the PX-EM algorithm over the classical EM algorithm disappears. We thus propose to stop the PX acceleration once |**λ**_*max*_| < *ϵ* with **λ**_*max*_ the dominant eigen value of ${\hat{\alpha }}^{\left(k+1\right)}-I_{q}$ and *ϵ* a pre-specified tolerance value (*e.g*. *ϵ* = 10^−2^).

#### Summary of the estimation procedure

The estimation procedure starts with a parameter ${\hat{\theta }}^{\left(0\right)}$, *k* = 0 and iterates the following six steps until convergence.

1. E-step: compute conditional expectations defined by Eqs (40)–(46) with ${\hat{\theta }}^{\left(k\right)}$.
2. PX M-step: obtain ${\hat{\theta }}^{\left(k+1\right)}$ and ${\hat{\alpha }}^{\left(k+1\right)}$ using Eqs (49)–(52) and the reduction function: ${\hat{\beta }}^{\left(k+1\right)}={\hat{\beta }}_{⋆}^{\left(k+1\right)}$, ${\hat{\delta }}_{\varepsilon }^{\left(k+1\right)}={\hat{\delta }}_{\varepsilon ⋆}^{\left(k+1\right)}$, ${\hat{\delta }}^{\left(k+1\right)}={\hat{\alpha }}^{\left(k+1\right)}{\hat{\delta }}_{⋆}^{\left(k+1\right)}$ and ${\hat{\bar{D}}}^{\left(k+1\right)}={\hat{\alpha }}^{\left(k+1\right)}{\hat{\bar{D}}}_{⋆}^{\left(k+1\right)}{\hat{\alpha }}^{(k+1)⊤}$.
3. Test: compute **λ**_*max*_ the dominant eigen value of ${\hat{\alpha }}^{\left(k+1\right)}-I_{q}$. If |**λ**_*max*_| < 10^−2^ then compute the marginal likelihood $\ell ({\hat{\theta }}^{\left(k+1\right)}|y)$ using Eq (36) and **go to 4)** with *k* = *k* + 1, otherwise **return to 1)** with *k* = *k* + 1.
4. E-step: compute conditional expectations defined by Eqs (40)–(46) with ${\hat{\theta }}^{\left(k\right)}$.
5. M-step: obtain ${\hat{\theta }}^{\left(k+1\right)}$ using Eqs (47)–(50).
6. Test: compute the marginal likelihood $\ell ({\hat{\theta }}^{\left(k+1\right)}|y)$ using Eqs (36). If $|\ell \left({\hat{\theta }}^{\left(k+1\right)}|y\right)-\ell \left({\hat{\theta }}^{\left(k\right)}|y\right)|/|\ell \left({\hat{\theta }}^{\left(k\right)}|y\right)|<10^{-6}$ then **go to 7)**, otherwise **return to 4)** with *k* = *k* + 1.
7. Rescaling: compute *υ*_0_ using (29) and rescale the estimates as $\hat{\beta }\leftarrow \upsilon_{0}{\hat{\beta }}^{\left(k\right)}$, $\hat{\delta }\leftarrow \upsilon_{0}{\hat{\delta }}^{\left(k\right)}$ and $\hat{\bar{D}}\leftarrow \upsilon_{0}^{2}{\hat{\bar{D}}}^{\left(k\right)}$. Return $\hat{\theta }={\left({\hat{\beta }}^{⊤},{\hat{\delta }}_{\epsilon },{\hat{\delta }}^{⊤},vech{\left(\hat{\bar{D}}\right)}^{⊤}\right)}^{⊤}$.

#### Approximate standard errors

With a view to allow asymptotic inference in SGTLM, we follow the empirical information-based method of [52] (pages 132-133) to compute the asymptotic variance-covariance matrix of the ML estimate $\hat{\theta }$ of ***θ*** under some general regularity conditions. The observed information matrix is defined to be $I_{o}\left(\hat{\theta }|y\right)=\sum_{i=1}^{n}{\hat{g}}_{i}{\hat{g}}_{i}^{⊤}$ where ${\hat{g}}_{i}=g_{i}\left(\hat{\theta }\right)$, $g_{i}\left(\theta \right)=\frac{\partial Q_{i}\left(\theta |\hat{\theta }\right)}{\partial \theta }$, $Q_{i}\left(\theta |\hat{\theta }\right)$ being the contribution of the single observation **y**_*i*_ to the expected complete data log-likelihood in Eq (39). On setting ${\hat{\bar{vub}}}_{i}={\hat{vub}}_{i}-c{\tilde{U}}_{1}{\hat{u_{2}b}}_{i}$, the elements $\frac{\partial Q_{i}\left(\theta |\hat{\theta }\right)}{\partial \beta },\frac{\partial Q_{i}\left(\theta |\hat{\theta }\right)}{\partial \delta_{\varepsilon }},\frac{\partial Q_{i}\left(\theta |\hat{\theta }\right)}{\partial \delta },vech(\frac{\partial Q_{i}\left(\theta |\hat{\theta }\right)}{\partial \bar{D}})$ of the score **g**_*i*_ can be explicitly evaluated using:

$$\begin{array}{ccc} \frac{\partial Q_{i}\left(\theta |\hat{\theta }\right)}{\partial \beta } & = & -[{\hat{u_{2}}}_{i}X_{i}^{⊤}X_{i}\beta -X_{i}^{⊤}({\hat{S_{1}}}_{i}-\delta_{\varepsilon }{\hat{S_{2}}}_{i}J_{n_{i}})], \end{array}$$ (53)

$$\begin{array}{ccc} \frac{\partial Q_{i}\left(\theta |\hat{\theta }\right)}{\partial \delta_{\varepsilon }} & = & -[{\hat{S_{2}}}_{i}J_{n_{i}}^{⊤}X_{i}\beta +\delta_{\varepsilon }n_{i}{\hat{S_{3}}}_{i}-J_{n_{i}}^{⊤}({\hat{S_{4}}}_{i}-c{\tilde{U}}_{1}{\hat{S_{1}}}_{i})], \end{array}$$ (54)

$$\begin{array}{ccc} \frac{\partial Q_{i}\left(\theta |\hat{\theta }\right)}{\partial \delta } & = & -{\bar{D}}^{-1}[{\hat{S_{3}}}_{i}\delta -{\hat{\bar{vub}}}_{i}], \end{array}$$ (55)

$$\begin{array}{ccc} \frac{\partial Q_{i}\left(\theta |\hat{\theta }\right)}{\partial \bar{D}} & = & -\frac{1}{2}{\bar{D}}^{-1}+\frac{1}{2}{\bar{D}}^{-1}[{\hat{u_{2}b_{2}}}_{i}-\delta {\hat{\bar{vub}}}_{i}^{⊤}-{\hat{\bar{vub}}}_{i}\delta^{⊤}+{\hat{S_{3}}}_{i}\delta \delta^{⊤}]{\bar{D}}^{-1}.\;\;\; \end{array}$$ (56)

Afterwards, the standard errors of estimated model parameters are approximated by square roots of diagonal elements of ${\left[I_{o}\left(\hat{\theta }|y\right)\right]}^{-1}$ and confidence intervals can be built assuming asymptotic normality.

#### Empirical Bayes estimators of random effects and weights

In this section, we provide the empirical Bayes estimators of cluster specific random effects and weights that are useful for evaluating individual intercepts and slopes as well as detecting outlying individuals. From Eq (27), the distribution of **b**_*i*_ conditional on **Z**_*i*_ = **z**_*i*_, *U*_*i*_ = *u*_*i*_ and *V*_*i*_ = *v*_*i*_ is multivariate normal with mean $r_{i}\left(z_{i}-X_{i}\beta \right)+(v_{i}u_{i}^{-1/2}-c{\tilde{U}}_{1})s_{i}$ and covariance matrix $u_{i}^{-1}\Lambda_{i}$ where $r_{i}=\bar{D}W_{i}^{⊤}{\bar{\Omega }}_{i}^{-1}$, **s**_*i*_ = *δ* − **r**_*i*_ Δ_*i*_, $\Delta_{i}=\upsilon_{0}\delta_{\varepsilon }J_{n_{i}}+W_{i}\delta$, ${\bar{\Omega }}_{i}=\upsilon_{0}^{2}I_{n_{i}}+W_{i}\bar{D}W_{i}^{⊤}$, and $\Lambda_{i}=(I_{q}-r_{i}W_{i})\bar{D}$. The conditional mean of **b**_*i*_ given **Y**_*i*_ = **y**_*i*_ is thus:

$$\begin{array}{ccc} {\bar{b}}_{i}\left(\theta \right) & = & E\left\{b_{i}|Y_{i}=y_{i},\theta \right\}=E\left\{E\left\{b_{i}|Z_{i}=z_{i},U_{i}=u_{i},V_{i}=v_{i},\theta \right\}|Y_{i}=y_{i},\theta \right\}\\ & = & r_{i}\left({\bar{z}}_{i}-X_{i}\beta \right)+({\bar{vu_{-1}}}_{i}-c{\tilde{U}}_{1})s_{i} \end{array}$$ (57)

where ${\bar{vu_{-1}}}_{i}=M_{i}({\bar{u\alpha }}_{i}+{\bar{\tau_{-1}}}_{i})$, ${\bar{u\alpha }}_{i}=M_{i}\Delta_{i}^{⊤}{\bar{\Omega }}_{i}^{-1}({\bar{uz}}_{i}-{\bar{u}}_{i}\mu_{i})$, $\mu_{i}=X_{i}\beta -c{\tilde{U}}_{1}\Delta_{i}$ and the quantities ${\bar{u}}_{i}=\text{E}\left\{U_{i}^{1/2}|y_{i},\theta \right\}$, ${\bar{uz}}_{i}=\text{E}\left\{U_{i}^{1/2}Z_{i}|y_{i},\theta \right\}$, ${\bar{\tau_{-1}}}_{i}=\text{E}\left\{U_{i}^{-1/2}\zeta_{1}\left(U_{i}^{1/2}\lambda^{⊤}Z_{0i}\right)|y_{i}\right\}$ and ${\bar{z}}_{i}=\text{E}\left\{Z_{i}|y_{i},\theta \right\}$ are to be evaluated using *Corollary 1* applied to $Z_{i}|Y_{i}=y_{i}∼{\text{TST}}_{n_{i}}(\mu_{i},\Omega_{i},\lambda_{i},\nu ,A_{i})$ with $\Omega_{i}={\bar{\Omega }}_{i}+\Delta_{i}\Delta_{i}^{⊤}$, $\lambda_{i}={\left(1+\Delta_{i}^{⊤}{\bar{\Omega }}_{i}^{-1}\Delta_{i}\right)}^{1/2}\Omega_{i}^{-1/2}\Delta_{i}$, $A_{i}=A_{i1}\times \ldots \times A_{in_{i}}$, $A_{ij}=\left(-\infty ,0\right]$ if *y*_*ij*_ = 0 and $A_{ij}=\left(0,\infty \right)$ if *y*_*ij*_ = 1. The empirical Bayes estimators of **b**_*i*_ can then be obtained as ${\hat{b}}_{i}={\bar{b}}_{i}\left(\hat{\theta }\right)$.

For outlying individuals detection, individual weights *U*_*i*_ are predicted by ${\bar{u_{2}}}_{i}=E\left\{U_{i}|y_{i},\theta \right\}$ [53] which is given by Eq (16) in *Corollary 1* applied to **Z**_*i*_|**Y**_*i*_ = **y**_*i*_. The empirical Bayes estimators of *U*_*i*_ are thus given by ${\hat{u_{2}}}_{i}=E\left\{U_{i}|y_{i},\hat{\theta }\right\}$. Relatively low weights (< 1) are indicative of outlying individuals.

## Applications

This section presents a simulation study for assessing performance of SGTLM, and an application of the modeling approach to a real dataset.

### Simulation study

We conducted a simulation to evaluate the proposed approach to the analysis of correlated binary data. The simulation experiment targeted four specific objectives. First, it assessed for different sample sizes, the abilities of the probit (PM), the skew-probit (SPM), the generalized t-link (GTLM) and the skew generalized t-link (SGTLM) models to recover population parameters when the common normality assumption for the link function is either violated or not. The widely used logistic model was not investigated as the logistic distribution can be considered as a special case of the Student t distribution [8] hence the logistic model is a special case of GTLM. Second, the experiment evaluated the extent to which asymptotic 95% confidence intervals (*CI*_95%_) can detect the presence of spurious skewness. Third, the experiment evaluated the ability of empirical Bayes estimators of random effects to predict true random effects. Finally, the simulation study assessed the ability of Akaike’s information criterion (*AIC*), Schwarz’s Bayesian information criterion (*BIC*) and Hannan-Quinn criterion (*HQ*) to select the correct model fit. All computations were performed in R.

#### Simulation design

Mimicking the structure of the simulation model studied in [24] (page 1116), we considered the following GLMM:

$$\begin{array}{ccc} Y_{ij} & = & I_{\left(0,\infty \right)}\left(Z_{ij}\right)\\ Z_{i}|b_{i},U_{i}=u_{i},V_{i}=v_{i} & \overset{ind}{∼} & N_{n_{i}}(\eta_{i}+[v_{i}u_{i}^{-1/2}-c{\tilde{U}}_{1}]\upsilon_{0}\delta_{\varepsilon }J_{n_{i}},\upsilon_{0}^{2}u_{i}^{-1}I_{n_{i}})\\ b_{i}|U_{i}=u_{i},V_{i}=v_{i} & \overset{ind}{∼} & N_{2}([v_{i}u_{i}^{-1/2}-c{\tilde{U}}_{1}]\delta ,u_{i}^{-1}\bar{D})\\ U_{i} & \overset{ind}{∼} & F_{U}\left(\nu \right)\;\text{and}\;V_{i}\overset{ind}{∼}\text{HN}\left(0,1\right) \end{array}$$

where ***η***_*i*_ = (*η*_*i*1_, ⋯, *η*_*i*6_)^⊤^, *η*_*ij*_ = *β*_0_ + *β*_1_ *X*_1*i*_ + *b*_0*i*_ + *b*_1*i*_ *W*_1*ij*_, **b**_*i*_ = (*b*_0*i*_, *b*_1*i*_)^⊤^; *X*_1_ is a dichotomous covariate (Bernoulli distribution with sucess probability 0.5) and *W*_1_ is a continuous occasion-varying random covariate (standard normal distribution); *β*_0_ is an intercept, *i.e*. the general mean of the linear predictor *η*_*ij*_ and *β*_1_ is the fixed-effects associated to the covariate *X*_1_ with values arbitrarily fixed to *β*_0_ = 1 and *β*_1_ = −1; *b*_0*i*_ is a random intercept associated to the cluster *i*, *b*_1*i*_ is the random slope associated to *W*_1*i*_; $\bar{D}$ is a 2 × 2 scale matrix with diagonal elements 0.5 and 1, and off diagonal element 0.25; $F_{U}\left(\nu \right)$ is a positive distribution with finite first two negative moments, *i.e*. ${\tilde{U}}_{t}=\text{E}\left\{U_{i}^{-t}\right\}<\infty$ for *t* = 1, 2; and $\upsilon_{0}={\left[{\tilde{U}}_{2}+\left({\tilde{U}}_{2}-c^{2}{\tilde{U}}_{1}^{2}\right)\delta_{\varepsilon }^{2}\right]}^{-1/2}$. We considered ***δ*** = (0, *δ*_1_)^⊤^, *i.e*. a null random intercept skewness to ensure the identifiability of the model.

Under this general class of SSMN latent models, we considered two data models. The first is the probit data model where *U* = 1, *δ*_1_ = 0 and *δ*_*ε*_ = 0 (probit link, *υ*_0_ = 1). The second is the skew generalized t-link data model with *δ*_*ε*_ = −2 and *δ*_1_ = 2 and U∼Gamma(ν/2,ν/2) with *ν* = 5 (*υ*_0_ = 0.4598). We considered for each data model, sample sizes (*n*) of 100, 500 and 1000 and thus generated three sets of covariates which were used for all simulations involving each of the two data models. Under each of the six resulting simulation settings, we generated 250 datasets to which we fitted the four fitting models under evaluation (PM, SPM, GTLM, SGTLM), considering the model degrees of freedom as known and equals to *ν* = 5 for the SGTLM. Fixed effects (*β*_0_ and *β*_1_) and skewness parameters (*δ*_*ϵ*_ and *δ*_1_) were initialized to zero whereas the scale matrix $\bar{D}$ was initialized to the 2×2 identity matrix.

#### Performance measures

In addition to estimates of fixed effects (${\hat{\beta }}_{k}$, *k* = 0, 1) and skewness (${\hat{\delta }}_{l}$ for SPM and SGTLM, *l* = *ϵ*, 1) and related empirical standard errors and *CI*_95%_, we recorded random effects variances ($\sigma_{1}^{2}$ of *b*_0*i*_ and $\sigma_{2}^{2}$ of *b*_1*i*_) and covariance (*σ*_12_) and their approximate standard errors derived using the delta method [54] as implemented in the R package *car* [55], empirical Bayes estimates of individual random effetcs (${\hat{b}}_{i}$), and the *AIC*, *BIC* and *HQ* criteria defined as: $AIC=-2\hat{\ell }+2N_{p}$, $BIC=-2\hat{\ell }+N_{p}\;\text{log}\;N$, and $HQ=-2\hat{\ell }+2N_{p}\;\text{log}\;\left(\;\text{log}\;N\right)$ where $\hat{\ell }$ is the maximized log-likelihood value, *N* is the total number of observations and *N*_*p*_ is the number of estimated model parameters. These data were used to compute various performance measures (Table 1) including the relative bias (%*Bias*) and the root mean square error (*RMSE*) in ${\hat{\beta }}_{k}$ and ${\hat{\sigma }}_{l}$; the standard deviations (SD) of ${\hat{\beta }}_{k}$; the quadratic mean ($\bar{SE}$) of standard errors of ${\hat{\beta }}_{k}$; the coverage probabilities ($\bar{CP}$) of ${\hat{\beta }}_{k}$ and ${\hat{\delta }}_{l}$, *i.e*. the proportion of times the *CI*_95%_ for *β*_*k*_ or *δ*_*l*_ included the true value; the arithmetic mean ($\bar{R^{2}}\left(b_{l},{\hat{b}}_{l}\right)$) of the square of Pearson’s correlation (coefficient of determination) between simulated and Bayes estimates of subject random effects; and the arithmetic means of information criteria AIC ($\bar{AIC}$), BIC ($\bar{BIC}$) and HQ ($\bar{HQ}$) across the 250 simulated datasets per simulation setting.

**Table 1:** Measures of the performance of binomial fitting models.

Measure	Formula	Role
$\%Bias({\hat{\sigma }}_{l})$	$100\times ({\bar{\hat{\sigma }}}_{b_{l}}-\sigma_{l})/\left|\sigma_{l}\right|$	Unbiasedness of ${\hat{\sigma }}_{l}$
$RMSE({\hat{\sigma }}_{l})$	$\sqrt{\sum_{r=1}^{250}{\left({\hat{\sigma }}_{l}^{\left(r\right)}-\sigma_{l}\right)}^{2}/250}$	Accuracy of ${\hat{\sigma }}_{l}$
$\%Bias({\hat{\beta }}_{k})$	$100\times ({\bar{\hat{\beta }}}_{k}-\beta_{k})/\left|\beta_{k}\right|$	Unbiasedness of ${\hat{\beta }}_{k}$
$RMSE({\hat{\beta }}_{k})$	$\sqrt{\sum_{r=1}^{250}{\left({\hat{\beta }}_{k}^{\left(r\right)}-\beta_{k}\right)}^{2}/250}$	Accuracy of ${\hat{\beta }}_{k}$
$SD({\hat{\beta }}_{k})$	$\sqrt{\sum_{r=1}^{250}{\left({\hat{\beta }}_{k}^{\left(r\right)}-{\bar{\hat{\beta }}}_{k})\right)}^{2}/249}$	Precision of ${\hat{\beta }}_{k}$
$\bar{SE}\left({\hat{\beta }}_{k}\right)$	$\sqrt{\sum_{r=1}^{250}se{\left({\hat{\beta }}_{k}^{\left(r\right)}\right)}^{2}/250}$	Reliability of estimated SE
$\bar{CP}\left({\hat{\beta }}_{k}\right)$	$\sum_{r=1}^{250}I_{CI^{\left(k,r\right)}}(\beta_{k})/250$	Coverage probability of ${\hat{\beta }}_{k}$
$\bar{CP}\left({\hat{\delta }}_{l}\right)$	$\sum_{r=1}^{250}I_{CI^{\left(k,r\right)}}(\delta_{l})/250$	Coverage probability of ${\hat{\delta }}_{l}$
$\bar{R^{2}}\left(b_{l},{\hat{b}}_{l}\right)$	$\sum_{r=1}^{250}R^{2}\left({\hat{b}}_{l}^{\left(r\right)},b_{l}^{\left(r\right)}\right)/250$	Predictive power
$\bar{AIC}$	$\sum_{r=1}^{250}AIC^{\left(r\right)}/250$	Model selection
$\bar{BIC}$	$\sum_{r=1}^{250}BIC^{\left(r\right)}/250$	Model selection
$\bar{HQ}$	$\sum_{r=1}^{250}HQ^{\left(r\right)}/250$	Model selection

Notes: SE = standard error, ${\bar{\hat{\beta }}}_{k}=\frac{\sum_{r=1}^{250}{\hat{\beta }}_{k}^{\left(r\right)}}{250}$; $b_{i}^{\left(r\right)}={\left(b_{i0}^{\left(r\right)},b_{i1}^{\left(r\right)}\right)}^{⊤}$ is the *r*^*th*^ simulated vector of random effects for the subject *i* and ${\hat{b}}_{i}^{\left(r\right)}$ is the empirical Bayes estimates for $b_{i}^{\left(r\right)}$; $R^{2}\left({\hat{b}}_{l}^{\left(r\right)},b_{l}^{\left(r\right)}\right)$ is the square of Pearson’s coefficient of correlation between ${\hat{b}}_{l}^{\left(r\right)}$ and $b_{l}^{\left(r\right)}$; $se({\hat{\beta }}_{k}^{\left(r\right)})$ is the empirical information-based standard error for ${\hat{\beta }}_{k}^{\left(r\right)}$ and *CI*^(*k*, *r*)^ is the 95% confidence interval for *β*_*k*_ from the *r*^*th*^ generated dataset. The same applies for *δ*_*l*_ and *σ*_*l*_.

#### Simulation results

Simulation results presented in Tables 2 and 3 show that under the probit data generation mechanism, the probit, the skew-probit, the generalized t (GT)-link and the skew generalized t (SGT)-link models recovered the population parameter values. Indeed, the percentage of bias was below 5% at all levels for fixed effects, whereas for variance components, the percentage of bias was below 20%. We particularly noticed a high relative bias in the variance component (%*Bias* = 17.33) under probit fit to probit data (assuming the true model) with small sample size (*n* = 100). This may be explained by the maximum likelihood estimation method which is known for providing biased variance components [56]. Nevertheless, this can be improved by opting for residual maximum likelihood estimation procedure [56]. However, it is worth noticing that the estimation improves as the sample size increases and the empirical standard error estimates agree with the standard deviations from the simulations. The results for *n* = 100 and *n* = 500 are consistent with findings in [24] where empirical information based standard errors approached Monte Carlo standard errors. Moreover, the 95% confidence interval for the skewness parameters allows to detect spurious skewness in the skew-probit and the SGT-link models with coverage probabilities of 100%. This result can be explained by the underlined high accuracy of information based standard errors in this type of model. The power in predicting random effects varied from *R*^2^ = 0.45 to *R*^2^ = 0.47 for random intercepts and from *R*^2^ = 0.23 to *R*^2^ = 0.28 for random slopes, but was comparable for the three fitting models. Finally, it appears that on average all model selection criteria correctly considered probit fitting model as the parsimonious model.

**Table 2:** Results based on 250 replications of probit samples: Probit and skew-probit fits.

Parameters	Measures	*n* = 100		*n* = 500		*n* = 1000	
		Probit	Skew probit	Probit	Skew probit	Probit	Skew probit
*β*_0_(−1)	Mean	-1.04 (0.25)	-1.03 (0.30)	-1.01 (0.12)	-1.04 (0.31)	-1.00 (0.08)	-1.00 (0.11)
	%Bias	-3.61	-3.01	-0.64	-4.03	-0.32	-0.23
	$\bar{SE}$	0.25 [0.23]	0.27 [0.26]	0.11 [0.12]	0.29 [0.24]	0.08 [0.08]	0.09 [0.11]
	CP	0.96	0.97	0.95	0.95	0.97	0.97
*β*_1_(1)	Mean	1.03 (0.24)	1.02 (0.41)	1.01 (0.10)	1.00 (0.41)	1.00 (0.07)	1.00 (0.42)
	%Bias	3.21	1.96	0.59	0.46	0.13	0.16
	$\bar{SE}$	0.23 [0.22]	0.43 [0.43]	0.1 [0.10]	0.48 [0.47]	0.07 [0.07]	0.08 [0.07]
	CP	0.95	0.96	0.97	0.97	0.97	0.97
$\sigma_{1}^{2}\left(0.5\right)$	Mean	0.54 (0.24)	0.51 (0.29)	0.51 (0.09)	0.53 (0.29)	0.50 (0.06)	0.51 (0.07)
	%Bias	7.98	2.24	1.39	2.35	0.12	1.06
*σ*_12_(0.25)	Mean	0.30 (0.34)	0.23 (0.48)	0.25 (0.13)	0.22 (0.48)	0.25 (0.09)	0.24 (0.49)
	%Bias	18.48	-6.52	1.84	-12.02	1.55	-2.02
$\sigma_{2}^{2}\left(1\right)$	Mean	1.17 (0.91)	1.10 (0.95)	1.02 (0.34)	1.11 (0.94)	1.02 (0.22)	1.00 (0.47)
	%Bias	17.33	9.94	2.42	10.98	1.96	0.99
*δ*_*ε*_(0)	Mean	-	0.02 (0.65)	-	0.02 (0.46)	-	0.00 (0.46)
	$\bar{SE}$	-	0.61 [0.62]	-	0.71 [0.51]	-	0.61 [0.46]
	CP	-	1.00	-	1.00	-	1.00
*δ*_1_(0)	Mean	-	-0.02 (0.37)	-	-0.02 (0.38)	-	-0.00 (0.25)
	$\bar{SE}$	-	2.00 [1.84]	-	2.00 [1.72]	-	2.00 [0.22]
	CP	-	1.00	-	1.00	-	1.00
$\bar{R^{2}}\left(b_{0},{\hat{b}}_{0}\right)$	Mean	0.45	0.46	0.46	0.46	0.46	0.46
$\bar{R^{2}}\left(b_{1},{\hat{b}}_{1}\right)$	Mean	0.23	0.24	0.26	0.26	0.27	0.28
*AIC*	Mean	548.29	552.14	3732.11	3733.80	7522.50	7524.04
*BIC*	Mean	570.27	582.92	3762.14	3775.85	7556.00	7570.94
*HQ*	Mean	556.85	564.12	3742.91	3748.92	7534.13	7540.33

Mean value and root mean square error (in parentheses), percentage of bias (%Bias), mean standard error (SE) and sample standard deviation (in square brackets), coverage probability (CP) of estimates, coefficient of determination (R2) and averages of AIC, BIC and HQ criteria for sample sizes (n) of 100, 500 and 1000.

Notes: In the first column (Parameters), model parameters are followed in parentheses by their respective true values;—means “not applicable”; the coverage probability (CP) is the probability that an approximate confidence interval (assuming asymptotic normality for the model parameter) contains the true parameter value.

**Table 3:** Results based on 250 replications of probit samples: Generalized t (GT)-link and skew generalized t (SGT)-link fits.

Parameters	Measures	*n* = 100		*n* = 500		*n* = 1000	
		GT-link	SGT-link	GT-link	SGT-link	GT-link	SGT-link
*β*_0_(−1)	Mean	-1.05 (0.26)	-1.05 (0.24)	-1.01 (0.11)	-1.03 (0.11)	-.1.00 (0.10)	-1.00 (0.08)
	%Bias	-4.95	-4.61	-1.06	-2.94	-0.17	-0.17
	$\bar{SE}$	0.25 [0.24]	0.27 [0.25]	0.15 [0.12]	0.11 [0.12]	0.08 [0.08]	0.06 [0.05]
	CP	0.98	0.97	0.97	0.98	0.97	0.97
*β*_1_(1)	Mean	1.03 (0.32)	1.03 (0.23)	1.01 (0.14)	1.03 (0.10)	1.00 (0.12)	1.00 (0.07)
	%Bias	3.16	3.28	1.62	2.90	0.08	0.13
	$\bar{SE}$	0.23 [0.20]	0.25 [0.22]	0.13 [0.13]	0.1 [0.10]	0.07 [0.07]	0.07 [0.07]
	CP	0.97	0.96	0.98	0.97	0.98	0.98
$\sigma_{1}^{2}\left(0.5\right)$	Mean	0.47 (0.12)	0.47 (0.10)	0.49 (0.11)	0.50 (0.09)	0.50 (0.05)	0.50 (0.03)
	%Bias	-5.36	-5.57	-1.76	0.01	0.17	0.12
*σ*_12_(0.25)	Mean	0.26 (0.53)	0.24 (0.40)	0.24 (0.22)	0.23 (0.02)	0.25 (0.19)	0.25 (0.02)
	%Bias	4.00	-4.82	-3.96	-6.26	-1.68	1.55
$\sigma_{2}^{2}\left(1\right)$	Mean	1.09 (.62)	0.99 (0.49)	1.00 (0.18)	0.99 (0.12)	0.99 (0.11)	0.99 (0.12)
	%Bias	8.98	-1.12	-0.78	-1.13	-0.87	-0.91
*δ*_*ε*_(0)	Mean	-	0.00 (0.56)	-	0.01 (0.54)	-	0.00 (0.41)
	$\bar{SE}$	-	0.44 [0.56]	-	0.46 [0.56]	-	0.48 [0.44]
	CP	-	1.00	-	1.00	-	1.00
*δ*_1_(0)	Mean	-	0.00 (0.18)	-	0.00 [0.14]	-	0.00 [0.14]
	$\bar{SE}$	-	1.22 [0.18]	-	0.99 [0.16]	-	0.62 [0.17]
	CP	-	1.00	-	1.00	-	1.00
$\bar{R^{2}}\left(b_{0},{\hat{b}}_{0}\right)$	Mean	0.45	0.46	0.47	0.46	0.47	0.47
$\bar{R^{2}}\left(b_{1},{\hat{b}}_{1}\right)$	Mean	0.26	0.27	0.27	0.27	0.27	0.28
*AIC*	Mean	552.09	554.37	3733.10	3736.21	7523.12	7525.21
*BIC*	Mean	581.43	589.55	3762.43	3784.26	7568.95	7573.26
*HQ*	Mean	561.97	568.06	3744.11	3764.90	7538.91	7542.49

Mean value and root mean square error (in parentheses), percentage of bias (%Bias), mean standard error (SE) and sample standard deviation (in square brackets), coverage probability (CP) of estimates, coefficient of determination (R2) and averages of AIC, BIC and HQ criteria for sample sizes (n) of 100, 500 and 1000.

Notes: In the first column (Parameters), model parameters are followed in parentheses by their respective true values;—means “not applicable”; the coverage probability (CP) is the probability that an approximate confidence interval (assuming asymptotic normality for the model parameter) contains the true parameter value.

Under a SGT-link data generation mechanism, the probit model performed poorly, showing large relative fixed effects bias values which decreased from 46% for samples of size *n* = 100 to 18% for samples of size *n* = 1000 (Table 4). The SGT-link model estimates were the less biased (%*Bias* < 12) as well as the most accurate with the lowest root mean square errors across all levels (Table 5). The same observations apply to variance components which were highly biased downward for probit, skew-probit and GT-link models (%*Bias* up to 90) relative to the SGT-link model (%*Bias* < 7). Regarding estimates of skewness parameters, the coverage probability was low (53% to 94%) for small sample size (*n* = 100) and approached nominal (95%) value for larger sample sizes (*n* = 500, 1000). The skew-probit model estimates (coverage probability down to 53%) were less reliable than estimates from the SGT-link model (coverage probability above 90%).

**Table 4:** Results based on 250 replications of skew generalized t-link samples (probit and skew-probit fits).

Parameters	Measures	*n* = 100		*n* = 500		*n* = 1000	
		Probit	Skew probit	Probit	Skew probit	Probit	Skew probit
*β*_0_(−1)	Mean	-1.46 (1.53)	-1.33 (1.30)	-1.28 (1.29)	-1.28 (1.31)	-1.18 (1.27)	-1.22 (0.92)
	%Bias	-46.18	-33.41	-28.30	-28.13	-18.27	-22.08
	$\bar{SE}$	0.95 [0.99]	0.89 [0.86]	0.96 [1.02]	0.89 [0.94]	0.97 [0.99]	0.92 [0.89]
	CP	0.93	0.95	0.95	0.96	0.96	0.96
*β*_1_(1)	Mean	0.95 (0.46)	0.97 (0.47)	0.95 (0.41)	0.98 (0.42)	0.95 (0.31)	0.98 (0.37)
	%Bias	-5.11	-3.31	-5.18	-2.11	-5.21	-2.11
	$\bar{SE}$	0.51 [0.48]	0.44 [0.48]	0.26 [0.39]	0.41 [0.43]	0.31 [0.29]	0.40 [0.41]
	CP	0.95	0.96	0.97	0.97	0.97	0.97
$\sigma_{1}^{2}\left(0.83\right)$	Mean	0.34 (0.94)	0.61 (0.39)	0.41 (0.91)	0.67 (0.38)	0.56 (0.76)	0.73 (0.34)
	%Bias	-59.04	-26.51	-50.60	-19.28	-32.53	-12.05
*σ*_12_(0.42)	Mean	0.80 (0.64)	0.53 (0.58)	0.81 (0.53)	0.42 (0.38)	0.75 (0.50)	0.40 (0.39)
	%Bias	90.48	26.19	92.86	0.09	78.57	-4.76
$\sigma_{2}^{2}\left(4.73\right)$	Mean	2.07 (1.19)	2.85 (1.08)	2.22 (1.44)	2.97 (1.99)	2.20 (1.32)	2.88 (1.70)
	%Bias	-56.24	-39.75	-53.07	-37.21	-53.49	-39.11
*δ*_*ε*_(−2)	Mean	-	-0.48 (1.75)	-	-0.91 (1.25)	-	-0.90 (1.04)
	%Bias	-	76.01	-	54.50	-	54.99
	$\bar{SE}$	-	0.60 [0.72]	-	1.23 [1.22]	-	1.03 [1.04]
	CP	-	0.87	-	0.91	-	0.95
*δ*_1_(2)	Mean	-	1.03 (0.97)	-	1.13 (0.97)	-	1.16 (0.92)
	%Bias	-	-48.51	-	-43.49	-	-42.00
	$\bar{SE}$	-	1.01 [1.00]	-	1.04 [1.01]	-	0.99 [0.96]
	CP	-	0.53	-	0.61	-	0.66
$\bar{R^{2}}\left(b_{0},{\hat{b}}_{0}\right)$	Mean	0.35	0.38	0.38	0.42	0.38	0.46
$\bar{R^{2}}\left(b_{1},{\hat{b}}_{1}\right)$	Mean	0.14	0.26	0.16	0.31	0.26	0.33
*AIC*	Mean	578.22	576.34	3824.31	3814.72	7662.07	7713.55
*BIC*	Mean	600.20	607.12	3854.34	3856.77	7695.56	7681.25
*HQ*	Mean	586.78	588.32	3835.11	3829.84	7673.70	7678.562

Mean value and root mean square error (in parentheses), percentage of bias (%Bias), mean standard error (SE) and sample standard deviation (in square brackets), coverage probability (CP) of estimates, coefficient of determination (R2) and averages of AIC, BIC and HQ criteria for sample sizes (n) of 100, 500 and 1000.

Notes: In the first column (Parameters), model parameters are followed by their respective true values in parentheses;—means “not applicable”; the coverage probability (CP) is the probability that an approximate confidence interval (assuming asymptotic normality for the model parameter) contains the true parameter value.

**Table 5:** Results based on 250 replications of skew generalized t-link samples (generalized t-link and skew generalized t-link fits).

Parameters	Measures	*n* = 100		*n* = 500		*n* = 1000	
		GT-link	SGT-link	GT-link	SGT-link	GT-link	SGT-link
*β*_0_(−1)	Mean	-1.40 (1.54)	-1.17 (1.31)	-1.25 (1.22)	-1.11 (1.09)	-1.11	-1.02 (0.09)
	%Bias	-39.17	-17.33	-24.56	-11.4	11.41	-2.09
	$\bar{SE}$	0.96 [0.89]	0.64 [0.55]	0.83 [0.92]	0.60 [0.51]	0.89 [0.86]	0.40[0.37]
	CP	0.93	0.96	0.95	0.98	0.96	0.98
*β*_1_(1)	Mean	0.98 (0.37)	1.01 (0.33)	0.99 (0.36)	1.01 (0.16)	0.98 (0.22)	1.00 (0.09)
	%Bias	-2.19	1.10	-1.14	1.23	-1.98	0.37
	$\bar{SE}$	0.32 [0.29]	0.28 [0.32]	0.23 [0.28]	0.11 [0.16]	0.28 [0.21]	0.09 [0.10]
	CP	0.95	0.96	0.97	0.97	0.97	0.98
$\sigma_{1}^{2}\left(0.83\right)$	Mean	0.37 (0.55)	0.86 (0.16)	0.40 (0.56)	0.84 (0.09)	0.53 (0.63)	0.84 (0.07)
	%Bias	-55.45	3.61	-51.80	1.20	-36.14	1.20
*σ*_12_(0.42)	Mean	0.68 (0.61)	0.36 (0.31)	0.71 (0.56)	0.38 (0.23)	0.66 (0.56)	0.38 (0.09)
	%Bias	61.91	-14.29	69.05	-9.52	57.14	-9.52
$\sigma_{2}^{2}\left(4.73\right)$	Mean	2.29 (1.23)	4.56 (1.51)	2.33 (1.17)	4.55 (1.29)	2.34 (1.11)	4.58 (1.31)
	%Bias	51.59	-3.59	-50.74	-3.81	-50.53	-3.17
*δ*_*ε*_(−2)	Mean	-	-2.18 (1.06)	-	-2.12 (1.04)	-	-2.06 (0.99)
	%Bias	-	-9.00	-	-6.02	-	-3.04
	$\bar{SE}$	-	1.44 [1.04]	-	1.04 [1.05]	-	0.88 [0.98]
	CP	-	0.94	-	0.96	-	0.97
*δ*_1_(2)	Mean	-	1.64 (1.18)	-	1.69 [1.16]	-	1.84 [1.04]
	%Bias	-	-18.13	-	-15.50	-	-8.10
	$\bar{SE}$	-	1.13 [1.18]	-	1.16 [1.17]	-	1.02 [1.03]
	CP	-	.90	-	0.94	-	0.96
$\bar{R^{2}}\left(b_{0},{\hat{b}}_{0}\right)$	Mean	0.34	0.53	0.38	0.56	0.40	0.58
$\bar{R^{2}}\left(b_{1},{\hat{b}}_{1}\right)$	Mean	0.22	0.49	0.26	0.52	0.26	0.52
*AIC*	Mean	578.04	524.06	3820.10	3804.12	7657.13	7652.56
*BIC*	Mean	601.18	597.24	3853.79	3852.17	7689.12	7671.17
*HQ*	Mean	588.01	575.75	3834.30	3821.40	7667.66	7542.49

Mean value and root mean square error (in parentheses), percentage of bias (%Bias), mean standard error (SE) and sample standard deviation (in square brackets), coverage probability (CP) of estimates, coefficient of determination (R2) and averages of AIC, BIC and HQ criteria for sample sizes (n) of 100, 500 and 1000.

Notes: In the first column (Parameters), model parameters are followed by their respective true values in parentheses;—means “not applicable”; the coverage probability (CP) is the probability that an approximate confidence interval (assuming asymptotic normality for the model parameter) contains the true parameter value.

Clearly, the SGT-link model adjusted better with non normal data and accordingly, random effects prediction is better with SGT-link model (*R*^2^ ≥ 0.49) than with the probit or the skew-probit model. Moreover, all the considered model selection criteria namely AIC, BIC and HQ on average correctly selected the SGT-link model as the preferred model.

### Application to the respiratory infection data

To demonstrate the usefulness of the proposed approach to correlated binary data modeling, we revisited the respiratory illness data (available in *geepack* package [57] in R) which was used by [24] to illustrate their t-link GLMM. The respiratory illness data was obtained from a clinical study of the effect of a treatment on 111 patients with respiratory illness, recruited from two different clinical centers. The patients were examined and their respiratory state (categorized as 1 = good, 0 = poor) determined (baseline). They were then randomized to receive either placebo or an active treatment. The goal of the study was to determine whether the treatment induced a better respiratory state in treated patients. The outcome is the respiratory state measured at four visits for each patient as good (*y* = 1) or poor (*y* = 0). In addition to the treatment (treat = 0 for placebo group (P) and treat = 1 for treated group (A)), the following fixed covariates were included: the clinical center (center = 0 for the first center and center = 1 for the second center), the baseline (respiratory state at the first visit), gender (sex = 0 for female (F) and sex = 1 for male (M)) and the interaction of treatment and gender. Following [24], we assumed that the age effect is patient-specific (random slope) and thus considered the patient age centered around its median (31 years) as a random covariate. Since the fixed covariates included binary variables (treat, gender, center and baseline), a conditional skew-probit model is not identifiable given random effects and we thus set *δ*_*ϵ*_ = 0 to ensure identifiability.

For the purpose of comparison, we fitted the probit, skew-probit, GT-link and SGT-link models. We initialized fixed effects ***β*** and the random slope skewness parameter *δ* to zero whereas the random slope scale was initialized to one. For the GT-link and the SGT-link models, we considered the model selection approach of [51] with degrees of freedom *ν* = 2.5, 2.6, …, 15.

**Fig 1:**
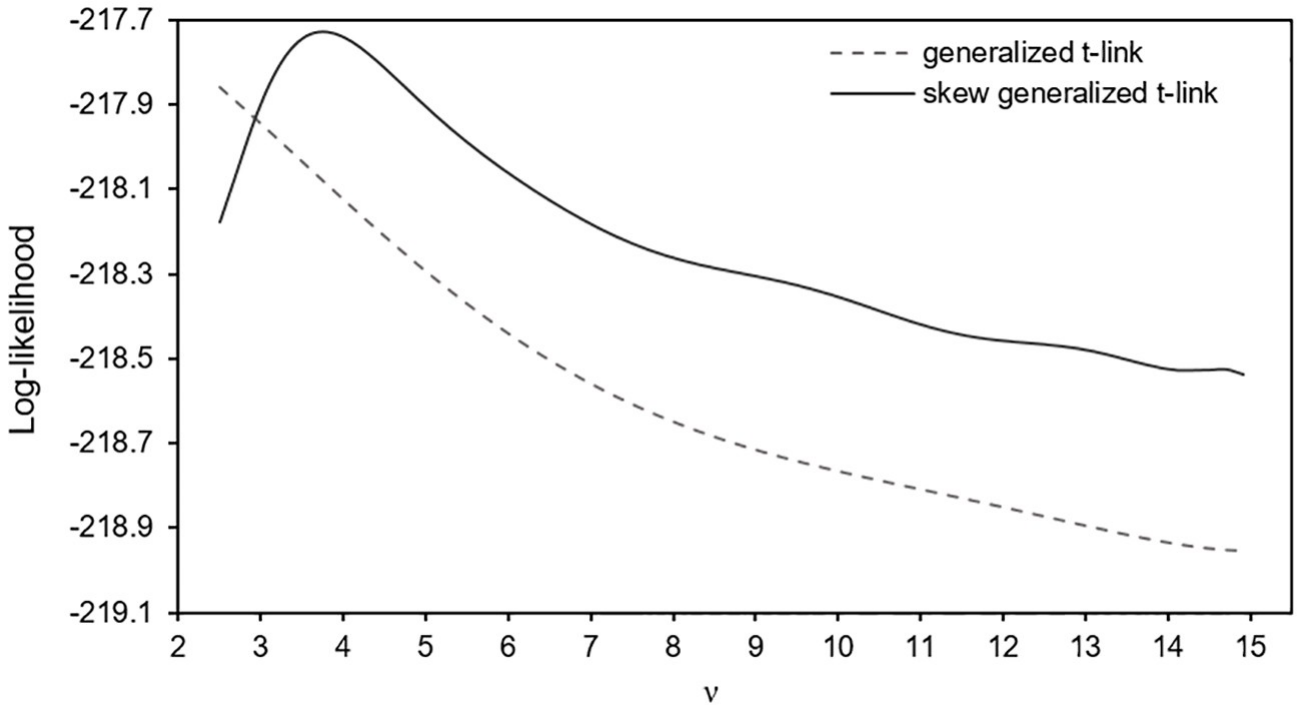
Fitting the generalized t-link and the skew generalized t models to the respiratory infection data: Plot of the marginal log-likelihood profiled for the degrees of freedom *ν*.

As depicted in Fig 1, the profiled marginal log-likelihood for the GT-link model is unbounded, with smaller *ν* corresponding to better fit in accordance with the t-link model fits in [24] (*ν* ≤ 4). We thus set *ν* = 2.5 for the t-link model. For the SGT-link fit, Fig 1 indicates that the log-likelihood is bounded with a maximum at *ν* = 3.7, suggesting heavy tail link function and random slope distributions. The difference in the behaviours of the GT-link and SGT-link models may be explained by the implication of *ν* in the location of the skew t-link model through ${\tilde{U}}_{1}$ (see Eq (32)).

The maximum likelihood (ML) estimates under the probit, skew-probit, GT-link and SGT-link models (Table 6) are somewhat close for the four fitted models which all show that respiratory illness is associated to clinical center, baseline state and treatment, with the treatment effect varying with gender. The SGT-link fit additionally indicates that, irrespective of the treatment, the respiratory state is poorer for male patients (Table 6, ${\hat{\beta }}_{3}<0$) than females. We notice for this dataset, that the intercept coefficient estimate increases with model complexity and estimates of fixed effects and their respective standard errors are shrunk toward zero for the skew-probit model relative to the probit one, and for the skew-probit model relative to the SGT-link one. The skew-probit model fit also gave a higher skewness ($\hat{\delta }=0.0411$) as compared with the SGT-link model fit ($\hat{\delta }=0.0262$). Although the estimated skewness is relatively low for both skew-probit and SGT-link models, the use of a skewed and heavy tail link clearly improved, not only the precision of estimates but also the adequacy between data and model. Indeed, the asymptotic 95% confidence interval for *δ* under the SGT-link includes zero (*CI*_95%_ = [−0.0010, 0.0534]), but we noticed from the simulation results that asymptotic *CI*_95%_ for skewness parameters becomes reliable only in large samples (*n* ≥ 500), whereas information criteria are reliable for all tested sample sizes. Thus, based on the AIC, BIC and HQ criteria in Table 6, the SGT-link fit is the best for the respiratory illness data. The estimate of the variance of the random slope of age is ${\hat{\sigma }}^{2}=0.0118$ for the SGT-link fit, with close values under probit and skew-probit models. From the SGT-link fit, it appears that the treatment induced an overall better respiratory state for treated patients (with a negative coefficient *β*_4_ = −2.0398 for the placebo group). Moreover, the treatment has on average a better effect on female patients than on male patients (with a positive coefficient, *β*_5_ = 1.3425 for male patients in the placebo group). However, as noted by [24], new studies are required to check this latter trend because of the highly unbalanced proportion of males (79%) and females (21%) in the data.

**Table 6:** Maximum likelihood fits of probit, skew-probit, Generalized T (GT)-link and Skew Generalized T (SGT) -link models to the respiratory infection data.

Parameter (variable)	Probit			Skew probit			GT-link (*ν* = 2.5)			SGT-link (*ν* = 3.7)		
	estimate	se	z value	estimate	se	z value	estimate	se	z value	estimate	se	z value
*β*_0_ (Intercept)	0.3429	0.4994	0.6866	0.4527	0.5012	0.9031	0.3570	0.5491	0.6502	0.5383	0.4659	1.1555
*β*_1_ (center = 2)	0.6292	0.2639	2.3846	0.6890	0.2611	2.6391	0.6767	0.2101	2.9410	0.5993	0.2395	2.5026
*β*_2_ (baseline)	1.6689	0.2900	5.7544	1.6312	0.2837	5.7499	1.8100	0.4118	4.3952	1.5152	0.2584	5.8627
*β*_3_ (sex = M)	-0.8228	0.5218	-1.5768	-1.0170	0.5293	-1.9216	-0.8833	0.5274	-1.6748	-1.0129	0.4944	-2.0489
*β*_4_ (treat = P)	-2.1198	0.6018	-3.5224	-2.0980	0.5958	-3.5215	-2.2634	0.7910	-2.8615	-2.0398	0.5366	-3.8010
*β*_5_ (M×P)	1.3840	0.6461	2.1419	1.4055	0.6403	2.1950	1.3251	0.6300	2.1033	1.3425	0.5867	2.2884
*δ* (age)	-	-	-	0.0411	0.0216	1.9011	-	-	-	0.0262	0.0139	1.8906
*σ* ^2^ (age)	0.0117	0.0050	-	0.0113	0.0049	-	0.0143	0.0061	-	0.0118	0.0059	-
*AIC*	455.6305	-	-	453.7766	-	-	453.2140	-	-	451.4658	-	-
*BIC*	484.3013	-	-	486.5432	-	-	485.9399	-	-	484.2324	-	-
*HQ*	466.9370	-	-	466.6983	-	-	465.7514	-	-	464.3875	-	-

Notes: M = male patient; P = placebo; M×P is a shortcut for sex = M×treat = P; *σ*^2^ and *δ* are the variance and the skewness parameter respectively for the patient-specific slope of median centered age (year); se = standard error; z value = estimate/se (a z value ≥1.96 roughly indicates 5% significance assuming asymptotic normality of z values);—means “not applicable”

## Conclusion

This work has considered the skew generalized t class of distributions for both link and random effects distributions in mixed models for binary data. The objective was to improve the exploitation of binary data bearing oddities such as skewness and tails thicker/thinner than the normal distribution. To allow inference in such models, we developped a maximum likelihood estimation procedure based on the EM algorithm. We combined results from [34] and [37] to obtained expressions for computing moments of truncated multivariate skew t distributions. The computation used existing R functions for the multivariate skew t cumulative distribution function. Our simulation experiment showed that, irrespective of sample size, the SGT-link model outperforms the probit GLMM when the underlying data generation mechanism is not normal. We also demonstrated that the skew generalized-link model performed better than the skew-probit and the generalized t-link GLMMs, when the underlying data is both skewed and heavy tailed.

An important finding is that when the model degrees of freedom *ν* is small and very large values are assumed (fitting probit and skew-probit models), the estimates of fixed effects are biased, whereas when *ν* is large but small values are assumed, the estimates of fixed effects are not biased. Moreover, asymptotic inference using information based standard errors proved highest ability accuracy in detecting spurious skewness in large samples (*n* ≥ 500) and information criteria on average selected the correct model fit for all tested sample sizes (*n* = 100, 500, 1000). These findings extend results in [24] on t-link GLMM to SGT-link GLMM, asserting that information criteria are reliable for selecting the best model for a particular dataset.

However, the simulation experiments revealed that the EM algorithm has a high computational cost. For instance, in a model with *q* = 2 random effects, *n* = 100 clusters and *n*_*i*_ = 6 observations per cluster, the mean running time for the SGT-link model fit was 4.76 minutes which is almost 135 times the time required by the probit model fit (2.12 seconds). Our implementation relies on the *pmst* function of the R package *sn* [35] to compute the cumulative probabilities of skew t distributions. This function uses the one dimensional routine *integral* of R on the multivariate normal cumulative distribution function. The use of the EM algorithm for large *q* values (*e.g*. *q* = 10, 15) requires the prior development of a faster routine for the computation of cumulative probabilities of skew t distributions. This will make the EM algorithm scalable for large *q* + *n*_*i*_. On multicore plateformes, parallel computing can also substantially speed computations up. The expressions provided for computing moments of truncated multivariate skew t distributions is limited to work for models with *ν* > 2. The use of formulae given in [38] will extend our EM algorithm to very small degrees of freedom (1 < *ν* ≤ 2).

Binary data related to very rare events often require special treatment and are generally analysed using zero inflated models [58]. The development of a skew generalized t-link model with zero inflation can significantly improve the exploitation of such data. In addition to binary data, GLMMs handle other data types like count, proportional and ordinal outcomes. From the good performance demonstrated in this work and in previous related ones [9, 24], we believe that the simultaneous introduction of flexible links and random effects distributions in GLMM would benefit knowledge extraction from observed data in applied research fields where advances rely on modeling capacity.

## Supporting information

S1 Appendix Proof of Lemma 1. This supporting information gives a proof of *Lemma 1*.
(PDF)

S2 Appendix Proof of Lemma 2. This supporting information gives a proof of *Lemma 2*.
(PDF)

S3 Appendix Proof of Proposition 1. This supporting information gives a proof of *Proposition 1*.
(PDF)

S4 Appendix Proof of Corollary 1. This supporting information gives a proof of *Corollary 1*.
(PDF)

S5 Appendix S5 Proof and limiting case of *Proposition 2*. This supporting information gives a proof of *Proposition 2*. The first two moments of truncated multivariate skew normal distributions (limiting case as *ν* → ∞) are also given (required for fitting skew-probit link models).
(PDF)

S6 Appendix Proof of Proposition 3. This supporting information gives a proof of *Proposition 3*.
(PDF)

S7 Appendix Proof of Proposition 4. This supporting information gives a proof of *Proposition 4*.
(PDF)

S8 Appendix Proof of Proposition 5. This supporting information gives a proof of *Proposition 5*.
(PDF)
