# Supplementary material for: Inference in skew generalized t-link models for clustered binary outcome via a parameter-expanded EM algorithm
Source: PLoS One. 2021 Apr 6;16(4):e0249604. doi: 10.1371/journal.pone.0249604 (PMC8028747; doi:10.1371/journal.pone.0249604)
Supplement: S1 Appendix — This supporting information gives a proof of Lemma 1. (PDF) [file pone.0249604.s001.pdf]

S1 Appendix for the manuscript "Inference in skew generalized t-link models for clustered binary outcome via a parameter-expanded EM algorithm"

Chénangnon F. Tovissodé [1*], Aliou Diop[2], Romain Glèlè Kakaï[1]

1 Laboratoire de Biomathématiques et d'Estimations Forestières, Faculté des Sciences Agronomiques, Université d'Abomey-Calavi, Abomey-Calavi, Bénin

2 Laboratoire d'Etudes et Recherches en Statistiques et Développement, Université Gaston Berger de Saint-Louis, Saint-Louis, Sénégal

* chenangnon@gmail.com

Note: Equation numbers refer to corresponding equations in the main text.

S1 Appendix: proof of *Lemma 1*

The condition *i*) of *Lemma 1* is an obvious requirement for the identifiability of a SSMN distribution. Note that condition *ii*) simply means that multiplication of any element of $\boldsymbol{\nu}$ by a positive scalar a is not equivalent to a multiplication of U by a. The condition *ii*) follows by the representation Eq (2) which is invariant only under simultaneous multiplication of U and $\bar{\boldsymbol{\Omega}}$ by any positive scalar a and multiplication of $\boldsymbol{\delta}$ by $\sqrt{a}$. Indeed, if *ii*) does not hold, then on denoting $\boldsymbol{\nu}_a$ the parameter $\boldsymbol{\nu}$ with ν_k replaced by $a\nu_k$, a SSMN vector $\mathbf{X}$ with parameters $\boldsymbol{\mu}$, $\sqrt{a}\boldsymbol{\delta}$, $a\bar{\boldsymbol{\Omega}}$ and $\boldsymbol{\nu}_a$ has the representation Eq (2) namely $\mathbf{X} = \boldsymbol{\mu} + (aU)^{-1/2}(\sqrt{a}\boldsymbol{\delta} z_0 + \sqrt{a}\mathbf{Z}) = \boldsymbol{\mu} + U^{-1/2}(\boldsymbol{\delta} z_0 + \mathbf{Z})$ which is independent of the choice of a hence the SSMN distribution is not determined by a unique set of parameters $\boldsymbol{\mu}$, $\boldsymbol{\delta}$, $\boldsymbol{\Omega}$ and $\boldsymbol{\nu}$. This proves the *only if* part of the lemma. The *if* part follows by rewriting Eq (2) as $\mathbf{Y} = \boldsymbol{\mu} + U^{-1/2} z_0 \boldsymbol{\delta} + U^{-1/2}\mathbf{Z}$ and observing that no simultaneous change in $\boldsymbol{\mu}$, $\boldsymbol{\delta}$ and/or $\boldsymbol{\Omega}$ can leave $\mathbf{Y}$ invariant.
