# Supplementary material for: Inference in skew generalized t-link models for clustered binary outcome via a parameter-expanded EM algorithm
Source: PLoS One. 2021 Apr 6;16(4):e0249604. doi: 10.1371/journal.pone.0249604 (PMC8028747; doi:10.1371/journal.pone.0249604)
Supplement: S2 Appendix — This supporting information gives a proof of Lemma 2. (PDF) [file pone.0249604.s002.pdf]

S2 Appendix for the manuscript "Inference in skew generalized t-link models for clustered binary outcome via a parameter-expanded EM algorithm"

Chénangnon F. Tovissodé [1*], Aliou Diop[2], Romain Glèlè Kakaï[1]

1 Laboratoire de Biomathématiques et d'Estimations Forestières, Faculté des Sciences Agronomiques, Université d'Abomey-Calavi, Abomey-Calavi, Bénin

2 Laboratoire d'Etudes et Recherches en Statistiques et Développement, Université Gaston Berger de Saint-Louis, Saint-Louis, Sénégal

* chenangnon@gmail.com

Note: Equation numbers refer to corresponding equations in the main text.

S2 Appendix: proof of *Lemma 2*

The pdf of a $\mathcal{ST}_p(\boldsymbol{\mu}, \boldsymbol{\Omega}^*, \boldsymbol{\lambda}, \nu)$ is obtained from Eq (11) by setting $\nu_0 = \nu$:

$$St_p(\mathbf{y}, \boldsymbol{\mu}, \boldsymbol{\Omega}^*, \boldsymbol{\lambda}, \nu) = \frac{2\,\Gamma\left(\frac{p+\nu}{2}\right) |\boldsymbol{\Omega}^*|^{-1/2} \nu^{\nu/2}}{\Gamma\left(\frac{\nu}{2}\right) \pi^{p/2} \left(\nu + \mathbf{y}_0^{*\top} \mathbf{y}_0^*\right)^{(p+\nu)/2}}\; T\left(\alpha^* \left(\frac{p+\nu}{\nu + \mathbf{y}_0^{*\top} \mathbf{y}_0^*}\right)^{1/2} \mid p+\nu\right)$$

with $\mathbf{y}_0^* = \boldsymbol{\Omega}^{*-1/2}\,(\mathbf{y} - \boldsymbol{\mu})$, $\alpha^* = \lambda^\top \mathbf{y}_0^*$.

We then notice that $\mathbf{y}_0^* = \left(\frac{\nu_0}{\nu}\right)^{-1/2} \boldsymbol{\Omega}^{-1/2}\,(\mathbf{y} - \boldsymbol{\mu}) = \sqrt{\frac{\nu}{\nu_0}} \mathbf{y}_0$ with $\mathbf{y}_0 = \boldsymbol{\Omega}^{-1/2}\,(\mathbf{y} - \boldsymbol{\mu})$. It subsequently follows that: (**a**) $\left(\nu + \mathbf{y}_0^{*\top} \mathbf{y}_0^*\right) = \left(\nu + \frac{\nu}{\nu_0} \mathbf{y}_0^\top \mathbf{y}_0\right) = \frac{\nu}{\nu_0}\left(\nu_0 + \mathbf{y}_0^\top \mathbf{y}_0\right)$; (**b**) $\alpha^* = \sqrt{\frac{\nu}{\nu_0}} \alpha$ with $\alpha = \lambda^\top \mathbf{y}_0$; (**c**) $\alpha^* \left(\frac{p+\nu}{\nu + \mathbf{y}_0^{*\top} \mathbf{y}_0^*}\right)^{1/2} = \sqrt{\frac{\nu}{\nu_0}} \alpha \left(\frac{p+\nu}{\frac{\nu}{\nu_0}\left(\nu_0 + \mathbf{y}_0^\top \mathbf{y}_0\right)}\right)^{1/2} = \alpha \left(\frac{p+\nu}{\nu_0 + \mathbf{y}_0^\top \mathbf{y}_0}\right)^{1/2}$. In addition, (**d**) $|\boldsymbol{\Omega}^*|^{-1/2} = |\frac{\nu_0}{\nu} \boldsymbol{\Omega}|^{-1/2} = \left[\left(\frac{\nu_0}{\nu}\right)^p |\boldsymbol{\Omega}|\right]^{-1/2} = \left(\frac{\nu}{\nu_0}\right)^{p/2} |\boldsymbol{\Omega}|^{-1/2}$. Replacing (**a**), (**b**), (**c**) and

(**d**) in $St_p(\mathbf{y}, \boldsymbol{\mu}, \boldsymbol{\Omega}^*, \boldsymbol{\lambda}, \nu)$ yields

$$\begin{aligned}
St_p(\mathbf{y}, \boldsymbol{\mu}, \boldsymbol{\Omega}^*, \boldsymbol{\lambda}, \nu) &= \frac{2\ \Gamma\left(\frac{p+\nu}{2}\right)\left(\frac{\nu}{\nu_0}\right)^{p/2} |\boldsymbol{\Omega}|^{-1/2} \nu^{\nu/2}}{\Gamma\left(\frac{\nu}{2}\right) \pi^{p/2} \left(\frac{\nu}{\nu_0}\right)^{(p+\nu)/2} \left(\nu_0 + \mathbf{y}_0^\top \mathbf{y}_0\right)^{(p+\nu)/2}} \\
&\quad \times T\left(\alpha\left(\frac{p+\nu}{\nu_0 + \mathbf{y}_0^\top \mathbf{y}_0}\right)^{1/2} \mid p+\nu\right) \\
&= \frac{2\ \Gamma\left(\frac{p+\nu}{2}\right) |\boldsymbol{\Omega}|^{-1/2} \nu_0^{\nu/2}}{\Gamma\left(\frac{\nu}{2}\right) \pi^{p/2} \left(\nu_0 + \mathbf{y}_0^\top \mathbf{y}_0\right)^{(p+\nu)/2}} \\
&\quad \times T\left(\alpha\left(\frac{p+\nu}{\nu_0 + \mathbf{y}_0^\top \mathbf{y}_0}\right)^{1/2} \mid p+\nu\right) \\
&= SGt_p\left(\mathbf{y} | \boldsymbol{\mu}, \boldsymbol{\Omega}, \boldsymbol{\lambda}, \boldsymbol{\nu}\right)
\end{aligned}$$

where the last line follows by Eq (10) and proves point *i*. Point *ii* of the lemma follows from point *i*. Indeed, since $\mathbf{X}$ and $\mathbf{Y} \sim \mathcal{ST}_p\left(\boldsymbol{\mu}, \boldsymbol{\Omega}^*, \boldsymbol{\lambda}, \nu\right)$ have the same sample space ($\mathbb{R}^p$), equality of pdfs implies equality of cdfs:
$SGT_p\left(\mathbf{x} | \boldsymbol{\mu}, \boldsymbol{\Omega}, \boldsymbol{\lambda}, \boldsymbol{\nu}\right) = \int_{\mathbb{A}} St_p(\mathbf{y}, \boldsymbol{\mu}, \boldsymbol{\Omega}^*, \boldsymbol{\lambda}, \nu) d\mathbf{y}$ giving
$SGT_p\left(\mathbf{x} | \boldsymbol{\mu}, \boldsymbol{\Omega}, \boldsymbol{\lambda}, \boldsymbol{\nu}\right) = ST_p(\mathbf{x}, \boldsymbol{\mu}, \boldsymbol{\Omega}^*, \boldsymbol{\lambda}, \nu)$ where the integration region is
$\mathbb{A} = \{\mathbf{y} \in \mathbb{R}^p |\ y_1 \leq x_1, y_2 \leq x_2, \cdots, y_p \leq x_p\}$ with x_k $(k = 1, 2, \cdots, p)$ the components of the vector $\mathbf{x}$.
