# Supplementary material for: Inference in skew generalized t-link models for clustered binary outcome via a parameter-expanded EM algorithm
Source: PLoS One. 2021 Apr 6;16(4):e0249604. doi: 10.1371/journal.pone.0249604 (PMC8028747; doi:10.1371/journal.pone.0249604)
Supplement: S3 Appendix — This supporting information gives a proof of Proposition 1. (PDF) [file pone.0249604.s003.pdf]

S3 Appendix for the manuscript "Inference in skew generalized t-link models for clustered binary outcome via a parameter-expanded EM algorithm"

Chénangnon F. Tovissodé [1*], Aliou Diop[2], Romain Glèlè Kakaï[1]

1 Laboratoire de Biomathématiques et d'Estimations Forestières, Faculté des Sciences Agronomiques, Université d'Abomey-Calavi, Abomey-Calavi, Bénin

2 Laboratoire d'Etudes et Recherches en Statistiques et Développement, Université Gaston Berger de Saint-Louis, Saint-Louis, Sénégal

* chenangnon@gmail.com

Note: Equation numbers refer to corresponding equations in the main text unless a source reference is specified.

S3 Appendix: proof of *Proposition 1*

To prove *Proposition 1*, we make use of the following result from corollary 1 in [1] (page 307).

Lemma 3

Let $\mathbf{Z} \sim \mathcal{ST}_p(\boldsymbol{\mu}, \boldsymbol{\Omega}, \boldsymbol{\lambda}, \nu)$ and $U \sim \mathcal{G}amma(\nu/2, \nu/2)$. Then for any real $r > -\nu$:

$$\begin{aligned}
\mathrm{E}\{U^r|\mathbf{Z}=\mathbf{z}\} &= \frac{f_0(\mathbf{z})}{f(\mathbf{z})} \frac{2^{r+1}\Gamma(\frac{p+\nu+2r}{2})}{\Gamma(\frac{p+\nu}{2})(\nu+\mathbf{z}_0^\top\mathbf{z}_0)^r} \\
&\quad \times T\left(\alpha\left(\frac{p+\nu+2r}{\nu+\mathbf{z}_0^\top\mathbf{z}_0}\right)^{1/2} |p+\nu+2r\right) \\
\mathrm{E}\{U^r\zeta_1(U^{1/2}\alpha)|\mathbf{Z}=\mathbf{z}\} &= \frac{f_0(\mathbf{z})}{f(\mathbf{z})} \frac{2^{(2r+1)/2}\ \Gamma(\frac{p+\nu+2r}{2})}{\pi^{1/2}\ \Gamma(\frac{p+\nu}{2})} \\
&\quad \times \frac{(\nu+\mathbf{z}_0^\top\mathbf{z}_0)^{(p+\nu)/2}}{(\nu+\mathbf{z}_0^\top\mathbf{z}_0+\alpha^2)^{(p+\nu+2r)/2}}
\end{aligned}$$

where $\mathbf{z}_0 = \boldsymbol{\Omega}^{-1/2}(\mathbf{z} - \boldsymbol{\mu})$, $\alpha = \boldsymbol{\lambda}^\top \mathbf{z}_0$, $f(\mathbf{z}) = SGt_p(\mathbf{z}|\boldsymbol{\mu}, \boldsymbol{\Omega}, \boldsymbol{\lambda}, \boldsymbol{\nu})$ is the pdf of $\mathbf{Z}$ given in Eq (10) and $f_0(\mathbf{z}) = Gt_p(\mathbf{z}|\boldsymbol{\mu}, \boldsymbol{\Omega}, \boldsymbol{\nu})$ is the pdf Eq in (11) with $\boldsymbol{\nu} = (\nu, \nu)^\top$.

Proof of Proposition 1

We first prove the proposition for the special case $\nu_0 = \nu$. By subsequently using the law of iterated expectations [2] (page 295, Eq 2) and *Lemma 3*, we have:

$$\begin{aligned}
\mathrm{E}\{U^{r/2} g(\mathbf{Z})\} &= \mathrm{E}\{\mathrm{E}\{U^{r/2}|\mathbf{Z} = \mathbf{z}\} g(\mathbf{Z})\} \\
&= \alpha_{st}^{-1} \int_{\mathbb{A}} \frac{f_0(\mathbf{z})\; 2^{\frac{r}{2}+1}\; \Gamma(\frac{p+\nu+r}{2})}{f(\mathbf{z})\Gamma(\frac{p+\nu}{2})(\nu + \mathbf{z}_0^\top \mathbf{z}_0)^{r/2}} T\left(\alpha \left(\frac{p+\nu+r}{\nu + \mathbf{z}_0^\top \mathbf{z}_0}\right)^{1/2} | p + \nu + r\right) \\
&\quad \times g(\mathbf{Z}) f(\mathbf{z}) d\mathbf{z}
\end{aligned}$$

The density $f(\mathbf{z})$ vanishes from the expression and we introduce $f_0(\mathbf{z}) = Gt_p(\mathbf{z}|\boldsymbol{\mu}, \boldsymbol{\Omega}, \boldsymbol{\nu})$ from Eq (11) (with $\boldsymbol{\nu} = (\nu, \nu)^\top$) for more simplifications through

$$\begin{aligned}
\frac{f_0(\mathbf{z})\; 2^{\frac{r}{2}+1}\; \Gamma(\frac{p+\nu+r}{2})}{\Gamma(\frac{p+\nu}{2})(\nu + \mathbf{z}_0^\top \mathbf{z}_0)^{r/2}} &= \frac{\Gamma(\frac{p+\nu}{2})|\boldsymbol{\Omega}|^{-1/2}\nu^{\nu/2}}{\Gamma(\nu/2)\pi^{p/2}\left(\nu + \mathbf{z}_0^\top \mathbf{z}_0\right)^{(p+\nu)/2}} \frac{2^{\frac{r}{2}+1}\; \Gamma(\frac{p+\nu+r}{2})}{\Gamma(\frac{p+\nu}{2})(\nu + \mathbf{z}_0^\top \mathbf{z}_0)^{r/2}} \\
&= \frac{2^{\frac{r}{2}+1}\; \Gamma(\frac{p+\nu+r}{2})|\boldsymbol{\Omega}|^{-1/2}\nu^{\nu/2}}{\Gamma(\nu/2)\pi^{p/2}(\nu + \mathbf{z}_0^\top \mathbf{z}_0)^{(p+\nu+r)/2}} \\
&= 2\; \frac{2^{r/2}}{\Gamma(\nu/2)} \frac{\Gamma(\frac{p+\nu+r}{2})|\boldsymbol{\Omega}|^{-1/2}\nu^{\nu/2}}{\pi^{p/2}(\nu + \mathbf{z}_0^\top \mathbf{z}_0)^{(p+\nu+r)/2}} \\
&= 2\; \frac{2^{r/2}\; \Gamma(\frac{\nu+r}{2})}{\Gamma(\nu/2)\; \nu^{r/2}} \frac{\Gamma(\frac{p+\nu+r}{2})|\boldsymbol{\Omega}|^{-1/2}\nu^{(\nu+r)/2}}{\Gamma(\frac{\nu+r}{2})\pi^{p/2}(\nu + \mathbf{z}_0^\top \mathbf{z}_0)^{(p+\nu+r)/2}}
\end{aligned}$$

where we have introduced on the last line $\nu^{r/2}$ in the numerator and $\Gamma(\frac{\nu+r}{2})$ in the denominator of the far right hand quotient in order to obtain the general form of $Gt_p(\mathbf{z}|\boldsymbol{\mu}, \boldsymbol{\Omega}, \boldsymbol{\nu})$ in Eq (11). After the introdution of $Gt_p(\mathbf{z}|\boldsymbol{\mu}, \boldsymbol{\Omega}, \boldsymbol{\nu}^*)$ with $\boldsymbol{\nu}^* = (\nu + r, \nu)^\top$, we set $\boldsymbol{\Omega}^* = \frac{\nu}{\nu+r}\boldsymbol{\Omega}$ and obtain from subsequently using Eq (10), point

i of *Lamma 1*, normalizing over $\mathbb{A}$, and using Eq (13)

$$\begin{aligned}
\mathrm{E}\{U^{r/2} g(\mathbf{Z})\} &= \alpha_{st}^{-1} \frac{2^{r/2}\Gamma(\frac{\nu+r}{2})}{\Gamma(\nu/2)\nu^{r/2}} \int_{\mathbb{A}} 2Gt_p(\mathbf{z}|\boldsymbol{\mu}, \boldsymbol{\Omega}, \boldsymbol{\nu}^*) \\
&\quad \times T\left(\alpha\left(\frac{p+\nu+r}{\nu+\mathbf{z}_0^\top \mathbf{z}_0}\right)^{1/2} |p+\nu+r\right) g(\mathbf{Z}) d\mathbf{z} \\
&= \frac{2^{r/2}\ \Gamma(\frac{\nu+r}{2})}{\nu^{r/2}\ \Gamma(\nu/2)}\ \alpha_{st}^{-1} \int_{\mathbb{A}} SGt_p(\mathbf{z}|\boldsymbol{\mu}, \boldsymbol{\Omega}, \boldsymbol{\lambda}, \boldsymbol{\nu}^*)\ g(\mathbf{z})\ d\mathbf{z} \\
&= C_r(\nu)\ \alpha_{st}^{-1} \int_{\mathbb{A}} St_p(\mathbf{z}|\boldsymbol{\mu}, \boldsymbol{\Omega}^*, \boldsymbol{\lambda}, \nu+r)\ g(\mathbf{z})\ d\mathbf{z} \\
&= C_r(\nu)\ \alpha_{st}^{-1}\ \alpha_{u,r} \int_{\mathbb{A}} \alpha_{u,r}^{-1} St_p(\mathbf{z}|\boldsymbol{\mu}, \boldsymbol{\Omega}^*, \boldsymbol{\lambda}, \nu+r)\ g(\mathbf{z})\ d\mathbf{z} \\
&= C_r(\nu)\ \alpha_{st}^{-1}\ \alpha_{u,r} \int_{\mathbb{A}} TSt_p(\mathbf{z}|\boldsymbol{\mu}, \boldsymbol{\Omega}^*, \boldsymbol{\lambda}, \nu+r, \mathbb{A})\ g(\mathbf{z})\ d\mathbf{z}
\end{aligned}$$

which proves Eq (14). Again, using the law of iterated expectations and *Lemma 3*, we have

$$\begin{aligned}
\mathrm{E}\{U^{r/2}\zeta_1(U^{1/2}\alpha)\ g(\mathbf{Z})\} &= \mathrm{E}\{\mathrm{E}\{U^{r/2}\zeta_1(U^{1/2}\alpha)|\mathbf{Z}=\mathbf{z}\}\ g(\mathbf{Z})\} \\
&= \alpha_{st}^{-1} \int_{\mathbb{A}} \frac{f_0(\mathbf{z}) 2^{(r+1)/2}\Gamma(\frac{p+\nu+r}{2})}{f(\mathbf{z})\pi^{1/2}\Gamma(\frac{p+\nu}{2})} \frac{(\nu+\mathbf{z}_0^\top \mathbf{z}_0)^{(p+\nu)/2}}{(\nu+\mathbf{z}_0^\top \mathbf{z}_0+\alpha^2)^{(p+\nu+r)/2}} \\
&\quad \times g(\mathbf{Z}) f(\mathbf{z}) d\mathbf{z}
\end{aligned}$$

After removal of $f(\mathbf{z})$, we introduce $f_0(\mathbf{z}) = Gt_p(\mathbf{z}|\boldsymbol{\mu}, \boldsymbol{\Omega}, \boldsymbol{\nu})$ and simplify through:

$$\begin{aligned}
& \frac{f_0(\mathbf{z})\ 2^{(r+1)/2}\Gamma(\frac{p+\nu+r}{2})}{\pi^{1/2}\Gamma(\frac{p+\nu}{2})} \frac{(\nu+\mathbf{z}_0^\top \mathbf{z}_0)^{(p+\nu)/2}}{(\nu+\mathbf{z}_0^\top \mathbf{z}_0+\alpha^2)^{(p+\nu+r)/2}} \\
=& \frac{\Gamma(\frac{p+\nu}{2})|\boldsymbol{\Omega}|^{-1/2}\nu^{\nu/2}}{\Gamma(\nu/2)\pi^{p/2}\left(\nu+\mathbf{z}_0^\top \mathbf{z}_0\right)^{(p+\nu)/2}} \frac{2^{(r+1)/2}\ \Gamma(\frac{p+\nu+r}{2})}{\pi^{1/2}\ \Gamma(\frac{p+\nu}{2})} \frac{(\nu+\mathbf{z}_0^\top \mathbf{z}_0)^{(p+\nu)/2}}{(\nu+\mathbf{z}_0^\top \mathbf{z}_0+\alpha^2)^{(p+\nu+r)/2}} \\
=& \frac{2^{(r+1)/2}}{\pi^{1/2}\ \Gamma(\nu/2)} \frac{\Gamma(\frac{p+\nu+r}{2})|\boldsymbol{\Omega}|^{-1/2}\nu^{\nu/2}}{\pi^{p/2}(\nu+\mathbf{z}_0^\top \mathbf{z}_0+\alpha^2)^{(p+\nu+r)/2}} \\
=& \left(\frac{2}{\pi}\right)^{1/2} \frac{2^{r/2}\Gamma(\frac{\nu+r}{2})}{\nu^{r/2}\Gamma(\nu/2)} \frac{\Gamma(\frac{p+\nu+r}{2})|\boldsymbol{\Omega}|^{-1/2}\nu^{(\nu+r)/2}}{\Gamma(\frac{\nu+r}{2})\pi^{p/2}(\nu+\mathbf{z}_0^\top \mathbf{z}_0+\alpha^2)^{(p+\nu+r)/2}} \\
=& c\ C_r(\nu)\ \frac{\Gamma(\frac{p+\nu+r}{2})|\boldsymbol{\Omega}|^{-1/2}\nu^{(\nu+r)/2}}{\Gamma(\frac{\nu+r}{2})\pi^{p/2}(\nu+\mathbf{z}_0^\top \mathbf{z}_0+\alpha^2)^{(p+\nu+r)/2}}
\end{aligned}$$

Next, on introducing $\alpha = \boldsymbol{\lambda}^\top \mathbf{z}_0 = \boldsymbol{\lambda}^\top \boldsymbol{\Omega}^{-1/2}(\mathbf{z}-\boldsymbol{\mu})$, we have

$$\begin{aligned}
\mathbf{z}_0^\top \mathbf{z}_0 + \alpha^2 &= (\mathbf{z}-\boldsymbol{\mu})^\top \boldsymbol{\Omega}^{-1}(\mathbf{z}-\boldsymbol{\mu}) + (\mathbf{z}-\boldsymbol{\mu})^\top \boldsymbol{\Omega}^{-1/2}\boldsymbol{\lambda}\boldsymbol{\lambda}^\top \boldsymbol{\Omega}^{-1/2}(\mathbf{z}-\boldsymbol{\mu}) \\
&= (\mathbf{z}-\boldsymbol{\mu})^\top \left[\boldsymbol{\Omega}^{-1} + \boldsymbol{\Omega}^{-1/2}\boldsymbol{\lambda}\boldsymbol{\lambda}^\top \boldsymbol{\Omega}^{-1/2}\right](\mathbf{z}-\boldsymbol{\mu})
\end{aligned}$$

We then notice that, thanks to the symmetry of $\mathbf{\Omega}$:

$$\begin{aligned}
\left[\mathbf{\Omega}^{-1} + \mathbf{\Omega}^{-1/2}\boldsymbol{\lambda}\boldsymbol{\lambda}^\top\mathbf{\Omega}^{-1/2}\right]^{-1} &= \left[\mathbf{\Omega}^{-1} + \left(\mathbf{\Omega}^{-1/2}\boldsymbol{\lambda}\right)\left(\mathbf{\Omega}^{-1/2}\boldsymbol{\lambda}\right)^\top\right]^{-1} \\
&= \mathbf{\Omega} - \frac{\mathbf{\Omega}\left(\mathbf{\Omega}^{-1/2}\boldsymbol{\lambda}\right)\left(\mathbf{\Omega}^{-1/2}\boldsymbol{\lambda}\right)^\top\mathbf{\Omega}}{1 + \left(\mathbf{\Omega}^{-1/2}\boldsymbol{\lambda}\right)^\top\mathbf{\Omega}\left(\mathbf{\Omega}^{-1/2}\boldsymbol{\lambda}\right)} \\
&= \mathbf{\Omega} - \frac{\left(\mathbf{\Omega}\mathbf{\Omega}^{-1/2}\boldsymbol{\lambda}\right)\left(\boldsymbol{\lambda}^\top\mathbf{\Omega}^{-1/2}\mathbf{\Omega}\right)}{1 + \boldsymbol{\lambda}^\top\mathbf{\Omega}^{-1/2}\mathbf{\Omega}\mathbf{\Omega}^{-1/2}\boldsymbol{\lambda}} \\
&= \mathbf{\Omega} - \frac{\mathbf{\Omega}^{1/2}\boldsymbol{\lambda}\boldsymbol{\lambda}^\top\mathbf{\Omega}^{1/2}}{1 + \boldsymbol{\lambda}^\top\boldsymbol{\lambda}} \\
&= \mathbf{\Omega} - \frac{\mathbf{\Omega}^{1/2}\boldsymbol{\lambda}}{\sqrt{1 + \boldsymbol{\lambda}^\top\boldsymbol{\lambda}}}\frac{\boldsymbol{\lambda}^\top\mathbf{\Omega}^{1/2}}{\sqrt{1 + \boldsymbol{\lambda}^\top\boldsymbol{\lambda}}} \\
&= \mathbf{\Omega} - \boldsymbol{\delta}\boldsymbol{\delta}^\top = \bar{\mathbf{\Omega}}
\end{aligned}$$

where the second line follows by the Sherman-Morrison identity [3] (page 121, Eq 3.1) and the last line follows from the definitions $\boldsymbol{\delta} = \left(1 + \boldsymbol{\lambda}^\top\boldsymbol{\lambda}\right)^{-1/2}\boldsymbol{\lambda}$ and $\bar{\mathbf{\Omega}} = \mathbf{\Omega} - \boldsymbol{\delta}\boldsymbol{\delta}^\top$ in Eq (2). It follows that $\mathbf{\Omega}^{-1} + \mathbf{\Omega}^{-1/2}\boldsymbol{\lambda}\boldsymbol{\lambda}^\top\mathbf{\Omega}^{-1/2} = \bar{\mathbf{\Omega}}^{-1}$ so that $\mathbf{z}_0^\top\mathbf{z}_0 + \alpha^2 = (\mathbf{z} - \boldsymbol{\mu})^\top\bar{\mathbf{\Omega}}^{-1}(\mathbf{z} - \boldsymbol{\mu}) = \bar{\mathbf{z}}_0^\top\bar{\mathbf{z}}_0$ on setting $\bar{\mathbf{z}}_0 = \bar{\mathbf{\Omega}}^{-1/2}(\mathbf{z} - \boldsymbol{\mu})$. Plus, $\mathbf{\Omega} = \bar{\mathbf{\Omega}} + \boldsymbol{\delta}\boldsymbol{\delta}^\top$ and therefore, by the Sherman-Morrison formula for the determinant [3] (page 123, Eq 3.6), $|\mathbf{\Omega}| = |\bar{\mathbf{\Omega}}|\left(1 + \boldsymbol{\delta}^\top\bar{\mathbf{\Omega}}^{-1}\boldsymbol{\delta}\right)$. Returning to the simplification with $\mathbf{z}_0^\top\mathbf{z}_0 + \alpha^2 = \bar{\mathbf{z}}_0^\top\bar{\mathbf{z}}_0$ and $|\mathbf{\Omega}|^{-1/2} = \left(1 + \boldsymbol{\delta}^\top\bar{\mathbf{\Omega}}^{-1}\boldsymbol{\delta}\right)^{-1/2}|\bar{\mathbf{\Omega}}|^{-1/2} = M|\bar{\mathbf{\Omega}}|^{-1/2}$ we get

$$\begin{aligned}
&\frac{f_0(\mathbf{z})\ 2^{(r+1)/2}\Gamma(\frac{p+\nu+r}{2})}{\pi^{1/2}\Gamma(\frac{p+\nu}{2})}\frac{\left(\nu + \mathbf{z}_0^\top\mathbf{z}_0\right)^{(p+\nu)/2}}{\left(\nu + \mathbf{z}_0^\top\mathbf{z}_0 + \alpha^2\right)^{(p+\nu+r)/2}} \\
= &\ c\ C_r(\nu)\left(1 + \boldsymbol{\delta}^\top\bar{\mathbf{\Omega}}^{-1}\boldsymbol{\delta}\right)^{-1/2}\frac{\Gamma(\frac{p+\nu+r}{2})|\bar{\mathbf{\Omega}}|^{-1/2}\nu^{(\nu+r)/2}}{\Gamma(\frac{\nu+r}{2})\pi^{p/2}\left(\nu + \bar{\mathbf{z}}_0^\top\bar{\mathbf{z}}_0\right)^{(p+\nu+r)/2}} \\
= &\ c\ C_r(\nu)M\ Gt_p\left(\mathbf{z}|\boldsymbol{\mu}, \bar{\mathbf{\Omega}}, \boldsymbol{\nu}\right)
\end{aligned}$$

where again $\boldsymbol{\nu} = (\nu + r, \nu)^\top$. We therefore have on setting $\bar{\mathbf{\Omega}}^* = \frac{\nu}{\nu+r}\bar{\mathbf{\Omega}}$ and subsequently using point i of *Lemma 2*, normalizing $t_p\left(\cdot|\boldsymbol{\mu}, \bar{\mathbf{\Omega}}^*, \nu + r\right)$ over $\mathbb{A}$ and using Eq (13)

$$\begin{aligned}
\mathrm{E}\{U^{r/2}\zeta_1(U^{1/2}\alpha)\ g(\mathbf{Z})\} &= c\ C_r(\nu)\ M\ \alpha_{st}^{-1}\int_{\mathbb{A}} Gt_p\left(\mathbf{z}|\boldsymbol{\mu}, \bar{\mathbf{\Omega}}, \boldsymbol{\nu}\right)g(\mathbf{z})d\mathbf{z} \\
&= cMC_r(\nu)\ \alpha_{st}^{-1}\int_{\mathbb{A}} t_p\left(\mathbf{z}|\boldsymbol{\mu}, \bar{\mathbf{\Omega}}^*, \nu + r\right)g(\mathbf{z})d\mathbf{z} \\
&= cMC_r(\nu)\ \alpha_{st}^{-1}\alpha_{\tau,r}\int_{\mathbb{A}} \alpha_{\tau,r}^{-1}\ t_p\left(\mathbf{z}|\boldsymbol{\mu}, \bar{\mathbf{\Omega}}^*, \nu + r\right)g(\mathbf{z})d\mathbf{z} \\
&= cMC_r(\nu)\ \alpha_{st}^{-1}\ \alpha_{\tau,r}\int_{\mathbb{A}} Tt_p\left(\mathbf{z}|\boldsymbol{\mu}, \bar{\mathbf{\Omega}}^*, \nu + r, \mathbb{A}\right)g(\mathbf{z})d\mathbf{z}
\end{aligned}$$

which proves Eq (15). For both Eq (14) and Eq (15), the condition $r > -\nu$ ensures $\frac{\nu}{\nu+r} > 0$, a requirement for $\mathbf{\Omega}^*$ and $\bar{\mathbf{\Omega}}^*$ to be positive definite.

The results for the general case where $\nu_0 \in (0, \infty)$ is obtained by replacing $\boldsymbol{\Omega}$ by $\frac{\nu_0}{\nu}\boldsymbol{\Omega}$ and U by $\frac{\nu}{\nu_0}U$ in the results for the special case $\nu_0 = \nu$.

References

1. Lachos VH, Ghosh P, Arellano-Valle RB. Likelihood based inference for skew-normal independent linear mixed models. Statistica Sinica. 2010;20:303–322.

2. Kaehler J. Laws of iterated expectations for higher order central moments. Statistical Papers. 1990;31(1):295–299.

3. Kéri G. The Sherman-Morrison formula for the determinant and its application for optimizing quadratic functions on condition sets given by extreme generators. In: Giannessi F, Pardalos P, T R, editors. Optimization Theory. Boston: Springer; 2001. p. 119–138.
