# Supplementary material for: Inference in skew generalized t-link models for clustered binary outcome via a parameter-expanded EM algorithm
Source: PLoS One. 2021 Apr 6;16(4):e0249604. doi: 10.1371/journal.pone.0249604 (PMC8028747; doi:10.1371/journal.pone.0249604)
Supplement: S4 Appendix — This supporting information gives a proof of Corollary 1. (PDF) [file pone.0249604.s004.pdf]

S4 Appendix for the manuscript "Inference in skew generalized t-link models for clustered binary outcome via a parameter-expanded EM algorithm"

Chénangnon F. Tovissodé [1*], Aliou Diop[2], Romain Glèlè Kakaï[1]

1 Laboratoire de Biomathématiques et d'Estimations Forestières, Faculté des Sciences Agronomiques, Université d'Abomey-Calavi, Abomey-Calavi, Bénin

2 Laboratoire d'Etudes et Recherches en Statistiques et Développement, Université Gaston Berger de Saint-Louis, Saint-Louis, Sénégal

* chenangnon@gmail.com

Note: Equation numbers refer to corresponding equations in the main text unless a source reference is specified.

S4 Appendix: proof of *Corollary 1*

To prove *Corollary 1*, we make use of the following lemma adapted from [2] (pages 4-5).

Lemma 4

Let $\mathbf{X} \sim \mathcal{TST}_p\left(\boldsymbol{\mu}, \boldsymbol{\Omega}, \boldsymbol{\lambda}, \nu, \mathbb{A}\right)$ with $\mathbb{A} = \mathbb{A}_1 \times \mathbb{A}_2 \times \cdots \times \mathbb{A}_p$, $\mathbb{A}_k \in \{(-\infty, a_k], \, (a_k, \infty)\}$. Let also set $\mathbf{A} = diag(A_1, \cdots, A_p)$ with elements $A_k = 1$ if $\mathbb{A}_k = (-\infty, a_k]$ and $A_k = -1$ if $\mathbb{A}_k = (a_k, \infty)$. Set $\mathbf{X}^* = \mathbf{A}\mathbf{X}$ and define $\mathbf{Y} \sim \mathcal{TST}_p\left(\mathbf{A}\boldsymbol{\mu}, \mathbf{A}\boldsymbol{\Omega}\mathbf{A}, \mathbf{A}\boldsymbol{\lambda}, \nu, \mathbf{A}\mathbf{a}\right)$.

Then, the truncation region of $\mathbf{X}^*$ is the right truncated hyperplane $\mathbb{A}^* = \mathbb{A}_1^* \times \mathbb{A}_2^* \times \cdots \times \mathbb{A}_p^*$ where $\mathbb{A}_k^* = (-\infty, A_k a_k] = (-\infty, a_k]$ if $A_k = 1$ and $\mathbb{A}_k^* = (-\infty, A_k a_k) = (-\infty, -a_k)$ if $A_k = -1$. Moreover $\mathbf{X}^* \overset{d}{=} \mathbf{Y}$ (where $\overset{d}{=}$ means "equal in distribution") and accordingly:

i. $\int_{\mathbb{A}} St_p\left(\mathbf{X} | \boldsymbol{\mu}, \boldsymbol{\Omega}, \boldsymbol{\lambda}, \nu\right) d\mathbf{X} = ST_p\left(\mathbf{A}\mathbf{a} | \mathbf{A}\boldsymbol{\mu}, \mathbf{A}\boldsymbol{\Omega}\mathbf{A}, \mathbf{A}\boldsymbol{\lambda}, \nu\right)$,

ii. $\mathrm{E}\{\mathbf{X}\} = \mathbf{A}\mathrm{E}\{\mathbf{Y}\}$, and

iii. $\mathrm{E}\{\mathbf{X}\mathbf{X}^\top\} = \mathbf{A}\mathrm{E}\{\mathbf{Y}\mathbf{Y}^{*\top}\}\mathbf{A}$.

Proof of Lemma 4

We notice and shall use through the proof the fact that the matrix $\mathbf{A}$ satisfies $\mathbf{A} = \mathbf{A}^\top = \mathbf{A}^{-1}$ and $\mathbf{A}^2 = \mathbf{I}_p$ since $\mathbf{A}$ is diagonal with elements equal to 1 or -1.

Truncation region of $\mathbf{X}^*$

The truncation region of $\mathbf{X}^*$ can be determined component wise because the linear transformation $\mathbf{X}^* = \mathbf{A}\mathbf{X}$ works component wise since $\mathbf{A}$ is diagonal. For any of the components of $\mathbf{X}^*$, we have one of the following two situations. *Case 1* with $\mathbb{A}_k = (-\infty, a_k]$: here $A_k = 1$ so that $\mathbf{X}^*$ and $\mathbf{X}$ have the same k^{th} marginal $X_k^* = X_k$, hence $\mathbb{A}_k^* = (-\infty, A_k a_k]$. *Case 2* with $\mathbb{A}_k = (a_k, \infty)$: here $A_k = -1$ so that $\mathbf{X}^*$ and $\mathbf{X}$ have opposite k^{th} marginals $X_k^* = -X_k$, hence the truncation interval of X_k^* is $\mathbb{A}_k^* = (-\infty, A_k a_k)$.

Equality in distribution of $\mathbf{X}^*$ *and* $\mathbf{Y}$

Let us consider a multivariate skew t vector $\mathbf{X}_0 \sim \mathcal{ST}_p(\boldsymbol{\mu}, \boldsymbol{\Omega}, \boldsymbol{\lambda}, \nu)$ which stands as the non-truncated version of $\mathbf{X}$. We also consider the random vector $\mathbf{X}_0^* = \mathbf{A}\mathbf{X}_0$, the non-truncated version of $\mathbf{X}_0$. Recall from Eq (2) the stochastic representation of $\mathbf{X}_0$:

$$\boldsymbol{X}_0 = \boldsymbol{\mu} + U^{-1/2}(\boldsymbol{\delta} z_0 + \mathbf{Z}), \quad z_0 \sim \mathcal{HN}(0,1), \quad \mathbf{Z} \sim \mathcal{N}_p(0, \bar{\boldsymbol{\Omega}})$$

where U is a gamma variable with both scale and shape parameters equal to $\nu/2$, z_0 is the standard half normal distribution; $\boldsymbol{\delta} = \left(1 + \boldsymbol{\lambda}^\top\boldsymbol{\lambda}\right)^{-1/2} \boldsymbol{\Omega}^{1/2}\boldsymbol{\lambda}$, $\bar{\boldsymbol{\Omega}} = \boldsymbol{\Omega} - \boldsymbol{\delta}\boldsymbol{\delta}^\top$; and z_0, $\mathbf{Z}$ and U are independent. Then, $\mathbf{X}_0^*$ has the representation

$$\boldsymbol{X}_0^* \quad = \quad \boldsymbol{\mu}^* + U^{-1/2}(\boldsymbol{\delta}^* z_0 + \mathbf{Z}^*), \quad z_0 \sim \mathcal{HN}(0,1), \quad \mathbf{Z}^* \sim \mathcal{N}_p(0, \bar{\boldsymbol{\Omega}}^*)$$

where we have set $\boldsymbol{\mu}^* = \mathbf{A}\boldsymbol{\mu}$, $\boldsymbol{\delta}^* = \mathbf{A}\boldsymbol{\delta}$ and $\bar{\boldsymbol{\Omega}}^* = \mathbf{A}\bar{\boldsymbol{\Omega}}\mathbf{A}$. $\boldsymbol{X}_0^*$ thus has a SGT distribution with location $\boldsymbol{\mu}^*$, scale $\boldsymbol{\Omega}^*$ and shape $\boldsymbol{\lambda}^*$ such that $\boldsymbol{\delta}^* = \left(1 + \boldsymbol{\lambda}^{*\top}\boldsymbol{\lambda}^*\right)^{-1/2} \boldsymbol{\Omega}^{*1/2}\boldsymbol{\lambda}^*$ and $\bar{\boldsymbol{\Omega}}^* = \boldsymbol{\Omega}^* - \boldsymbol{\delta}^*\boldsymbol{\delta}^{*\top}$. Applying Eq (8) with $\bar{\boldsymbol{\Omega}}^*$ and $\boldsymbol{\delta}^*$ gives $\boldsymbol{\Omega}^* = \bar{\boldsymbol{\Omega}}^* + \boldsymbol{\delta}^*\boldsymbol{\delta}^{*\top}$ which develops as $\boldsymbol{\Omega}^* = \mathbf{A}\bar{\boldsymbol{\Omega}}\mathbf{A} + \mathbf{A}\boldsymbol{\delta}\boldsymbol{\delta}^\top\mathbf{A} = \mathbf{A}\left(\bar{\boldsymbol{\Omega}} + \boldsymbol{\delta}\boldsymbol{\delta}^\top\right)\mathbf{A}$ hence $\boldsymbol{\Omega}^* = \mathbf{A}\boldsymbol{\Omega}\mathbf{A}$. The result in Eq (8) also gives $\boldsymbol{\lambda}^* = \left(1 - \boldsymbol{\delta}^{*\top}\boldsymbol{\Omega}^{*-1}\boldsymbol{\delta}\right)^{-1/2} \boldsymbol{\Omega}^{*-1/2}\boldsymbol{\delta}^*$. We then develop and reduce $\boldsymbol{\delta}^{*\top}\boldsymbol{\Omega}^{*-1}\boldsymbol{\delta}^*$ as $\boldsymbol{\delta}^{*\top}\boldsymbol{\Omega}^{*-1}\boldsymbol{\delta}^* = \boldsymbol{\delta}^\top\mathbf{A}\mathbf{A}\boldsymbol{\Omega}^{-1}\mathbf{A}\mathbf{A}\boldsymbol{\delta} = \boldsymbol{\delta}^\top\boldsymbol{\Omega}^{-1}\boldsymbol{\delta}$. We furthermore have $\boldsymbol{\Omega}^{*-1/2} = \left(\mathbf{A}\boldsymbol{\Omega}\mathbf{A}\right)^{-1/2}$.

In order to simplify the expression of $\boldsymbol{\Omega}^{*-1/2}$, note that $\mathbf{A}\boldsymbol{\Omega}\mathbf{A} = \mathbf{A}\boldsymbol{\Omega}^{1/2}\boldsymbol{\Omega}^{1/2}\mathbf{A}$ which on using $\mathbf{A}^2 = \mathbf{I}_p$ becomes $\mathbf{A}\boldsymbol{\Omega}\mathbf{A} = \mathbf{A}\boldsymbol{\Omega}^{1/2}\mathbf{A}\mathbf{A}\boldsymbol{\Omega}^{1/2}\mathbf{A} = \left(\mathbf{A}\boldsymbol{\Omega}^{1/2}\mathbf{A}\right)^2$. This yields

$(\mathbf{A\Omega A})^{-1/2} = \left(\mathbf{A\Omega}^{1/2}\mathbf{A}\right)^{-1}$ which on using twice the inverse of the Cayley product rule [3] (page 93, Eq 3.132), simplifies as

$$\begin{aligned}\left(\mathbf{A\Omega}^{1/2}\mathbf{A}\right)^{-1} &= \left[\mathbf{A}(\mathbf{\Omega}^{1/2}\mathbf{A})\right]^{-1}\\ &= (\mathbf{\Omega}^{1/2}\mathbf{A})^{-1}\mathbf{A}^{-1}\\ &= \mathbf{A}^{-1}\mathbf{\Omega}^{-1/2}\mathbf{A}^{-1}\\ &= \mathbf{A\Omega}^{-1/2}\mathbf{A}.\end{aligned}$$

Hence $\mathbf{\Omega}^{*-1/2} = \mathbf{A\Omega}^{-1/2}\mathbf{A}$. Combining the last equality with $\boldsymbol{\delta}^{*\top}\mathbf{\Omega}^{*-1}\boldsymbol{\delta}^{*} = \boldsymbol{\delta}^{\top}\mathbf{\Omega}^{-1}\boldsymbol{\delta}$ in the expression $\boldsymbol{\lambda}^{*} = (1-\boldsymbol{\delta}^{*\top}\mathbf{\Omega}^{*-1}\boldsymbol{\delta})^{-1/2}\ \mathbf{\Omega}^{*-1/2}\boldsymbol{\delta}^{*}$ results in the shape

$$\begin{aligned}\boldsymbol{\lambda}^{*} &= (1-\boldsymbol{\delta}^{*\top}\mathbf{\Omega}^{*-1}\boldsymbol{\delta})^{-1/2}\ \mathbf{\Omega}^{*-1/2}\boldsymbol{\delta}^{*}\\ &= \left(1-\boldsymbol{\delta}^{\top}\mathbf{\Omega}^{-1}\boldsymbol{\delta}\right)^{-1/2}\mathbf{A\Omega}^{-1/2}\mathbf{AA}\boldsymbol{\delta}\\ &= \left(1-\boldsymbol{\delta}^{\top}\mathbf{\Omega}^{-1}\boldsymbol{\delta}\right)^{-1/2}\mathbf{A\Omega}^{-1/2}\boldsymbol{\delta}\\ &= \mathbf{A}\boldsymbol{\lambda}.\end{aligned}$$

Overall, $\mathbf{X}_0^{*} \sim \mathcal{ST}_p\left(\mathbf{A}\boldsymbol{\mu}, \mathbf{A\Omega A}, \mathbf{A}\boldsymbol{\lambda}, \nu\right)$. Therefore, since $\mathbf{X}^{*}$ is the truncated version of $\mathbf{X}_0^{*}$ with truncation region $\mathbb{A}^{*}$, it comes that $\mathbf{X}^{*} \sim \mathcal{TST}_p\left(\mathbf{A}\boldsymbol{\mu}, \mathbf{A\Omega A}, \mathbf{A}\boldsymbol{\lambda}, \nu, \mathbb{A}^{*}\right)$.

Next, recall that $\mathbf{Y} \sim \mathcal{TST}_p\left(\mathbf{A}\boldsymbol{\mu}, \mathbf{A\Omega A}, \mathbf{A}\boldsymbol{\lambda}, \nu, \mathbf{Aa}\right)$ with truncation region the right truncated hyperplane $\mathbb{A}_{\mathbf{Y}} = (-\infty, A_1 a_1] \times (-\infty, A_2 a_2] \times \cdots \times (-\infty, A_p a_p]$. We notice that $\mathbf{X}^{*}$ and $\mathbf{Y}$ have the same density function over $\mathbb{A}^{*}$ (sample space of $\mathbf{X}^{*}$) which satisfies $\mathbb{A}^{*} \subset \mathbb{A}_{\mathbf{Y}}$ and differs from $\mathbb{A}_{\mathbf{Y}}$ only on the boundary of $\mathbb{A}_{\mathbf{Y}}$ since for components of $\mathbf{X}^{*}$ with $A_k = -1$, the truncation interval is the open $(-\infty, A_k a_k)$ which does not include $A_k a_k$ whereas $\mathbb{A}_{\mathbf{Y}}$ includes all points $A_k a_k$. Also notice that $p-$variate ST random vectors are special cases of $p-$variate skew elliptical vectors which possess moment generating functions defined on $\mathbb{R}^p$ [4] (page 107). This implies that truncated $p-$variate ST random vectors also possess moment generating functions on $\mathbb{R}^p$. Then, since both $\mathbf{X}^{*}$ and $\mathbf{Y}$ are continuous random vectors, we have equality of their moment generating functions as follows, for $\mathbf{t} \in \mathbb{R}^p$:

$$\begin{aligned}\mathrm{E}\{e^{\mathbf{t}^{\top}\mathbf{X}^{*}}\} &= \int_{\mathbb{A}^{*}} e^{\mathbf{t}^{\top}\mathbf{x}}\, TSt_p\left(\mathbf{x}|\mathbf{A}\boldsymbol{\mu}, \mathbf{A\Omega A}, \mathbf{A}\boldsymbol{\lambda}, \nu, \mathbb{A}^{*}\right) d\mathbf{x}\\ \mathrm{E}\{e^{\mathbf{t}^{\top}\mathbf{X}^{*}}\} &= \int_{\mathbb{A}_{\mathbf{Y}}} e^{\mathbf{t}^{\top}\mathbf{x}}\, TSt_p\left(\mathbf{x}|\mathbf{A}\boldsymbol{\mu}, \mathbf{A\Omega A}, \mathbf{A}\boldsymbol{\lambda}, \nu, \mathbb{A}^{*}\right) d\mathbf{x}\\ &= \int_{\mathbb{A}_{\mathbf{Y}}} e^{\mathbf{t}^{\top}\mathbf{x}}\, TSt_p\left(\mathbf{x}|\mathbf{A}\boldsymbol{\mu}, \mathbf{A\Omega A}, \mathbf{A}\boldsymbol{\lambda}, \nu, \mathbf{Aa}\right) d\mathbf{x}\\ &= \mathrm{E}\{e^{\mathbf{t}^{\top}\mathbf{Y}^{*}}\}\end{aligned}$$

where on the second line we ignore the boundary difference between $\mathbb{A}_{\mathbf{Y}}$ and $\mathbb{A}^*$ and on the third line, we replace the general truncated ST density by the righ truncated density (since $\mathbb{A}_{\mathbf{Y}}$ is a right truncated hyperplane). A a result, since the moment generating function uniquely determines the probability distribution, it follows that $\mathbf{X}^*$ and $\mathbf{Y}$ are equal in distribution.

Points *i*, *ii* and *iii*

Let $\bar{\mathbb{A}}$ the open subset of $\mathbb{A}$ defined as $\bar{\mathbb{A}} = \bar{\mathbb{A}}_1 \times \bar{\mathbb{A}}_2 \times \cdots \times \bar{\mathbb{A}}_p$ where $\bar{\mathbb{A}}_k = (-\infty, a_k)$ if $\mathbb{A}_k = (-\infty, a_k]$ and $\bar{\mathbb{A}}_k = \mathbb{A}_k$ if $\mathbb{A}_k = (a_k, \infty)$. Since the probability that $X_{0k} = a_k$ is zero, *i.e.* $P_{X_{0k}}(X_{0k} = a_k) = 0$ for $k = 1, 2, ..., p$; we have the equality $P_{\mathbf{X}_0}(\mathbf{X}_0 \in \mathbb{A}) = P_{\mathbf{X}_0}(\mathbf{X}_0 \in \bar{\mathbb{A}})$. Then, since $\mathbf{X}_0 \sim \mathcal{ST}_p\left(\boldsymbol{\mu}, \boldsymbol{\Omega}, \boldsymbol{\lambda}, \nu\right)$,

$$\int_{\mathbb{A}} St_p\left(\mathbf{X}|\boldsymbol{\mu}, \boldsymbol{\Omega}, \boldsymbol{\lambda}, \nu\right) d\mathbf{X} = P_{\mathbf{X}_0}(\mathbf{X}_0 \in \mathbb{A}) = P_{\mathbf{X}_0}(\mathbf{X}_0 \in \bar{\mathbb{A}}).$$

Next, $X_{0k} \in \bar{\mathbb{A}}_k \iff X^*_{0k} < A_k a_k$ since $X^*_{0k} = A_k X_{0k}$ and the interval $\bar{\mathbb{A}}_k$ is open at its boundary a_k. We thus have $\mathbf{X}_0 \in \bar{\mathbb{A}} \iff X^*_0 \in A^*$. This equivalence yields $P_{\mathbf{X}_0}(\mathbf{X}_0 \in \bar{\mathbb{A}}) = P_{\mathbf{X}^*_0}(\mathbf{X}^*_0 \in \mathbb{A}^*)$. Then, since $\mathbf{X}^*_0 \sim \mathcal{ST}_p\left(\mathbf{A}\boldsymbol{\mu}, \mathbf{A}\boldsymbol{\Omega}\mathbf{A}, \mathbf{A}\boldsymbol{\lambda}, \nu\right)$ is continuous over $\mathbb{R}^p$, we have $P_{\mathbf{X}^*_0}(\mathbf{X}^*_0 \in \mathbb{A}^*) = P_{\mathbf{X}^*_0}(\mathbf{X}^*_0 \in \mathbb{A}_{\mathbf{Y}}) = ST_p\left(\mathbf{A}\mathbf{a}|\mathbf{A}\boldsymbol{\mu}, \mathbf{A}\boldsymbol{\Omega}\mathbf{A}, \mathbf{A}\boldsymbol{\lambda}, \nu\right)$. We thus end up with

$$\int_{\mathbb{A}} St_p\left(\mathbf{X}|\boldsymbol{\mu}, \boldsymbol{\Omega}, \boldsymbol{\lambda}, \nu\right) d\mathbf{X} = ST_p\left(\mathbf{A}\mathbf{a}|\mathbf{A}\boldsymbol{\mu}, \mathbf{A}\boldsymbol{\Omega}\mathbf{A}, \mathbf{A}\boldsymbol{\lambda}, \nu\right)$$

which proves point *i*. Furthermore, from $\mathbf{X}^* = \mathbf{A}\mathbf{X}$, the first two moments of $\mathbf{X}^*$ are related to those of $\mathbf{X}$ via $\mathrm{E}\{\mathbf{X}^*\} = \mathbf{A}\mathrm{E}\{\mathbf{X}\}$ and $\mathrm{E}\{\mathbf{X}^*\mathbf{X}^{*\top}\} = \mathbf{A}\mathrm{E}\{\mathbf{X}\mathbf{X}^\top\}\mathbf{A}$ (since $\mathbf{A} = \mathbf{A}^\top$). Hence the identity $\mathbf{A}^{-1} = \mathbf{A}$ implies that $\mathrm{E}\{\mathbf{X}\} = \mathbf{A}\mathrm{E}\{\mathbf{X}^*\}$ and $\mathrm{E}\{\mathbf{X}\mathbf{X}^\top\} = \mathbf{A}\mathrm{E}\{\mathbf{X}^*\mathbf{X}^{*\top}\}\mathbf{A}$. Finally, the fact that $\mathbf{X}^*$ and $\mathbf{Y}$ have the same moment generating function, implies that we can replace moments of $\mathbf{X}^*$ by those of $\mathbf{Y}$.

Proof of Corollary 1

We first prove the corollary for the special case $\nu_0 = \nu$. Define for any vector $\mathbf{X}$: $\mathbf{X}^{(0)} = 1$, $\mathbf{X}^{(1)} = \mathbf{X}$ and $\mathbf{X}^{(2)} = \mathbf{X}\mathbf{X}^\top$ and recall the definitions of targeted expectations $\overline{u_r\mathbf{z}_s} = \mathrm{E}\{U^{r/2}\mathbf{Z}^{(s)}\}$ and $\overline{\tau_r\mathbf{z}_s} = \mathrm{E}\{U^{r/2}\zeta_1(U^{1/2}\alpha)\mathbf{Z}^{(s)}\}$ for $s \in \{0, 1, 2\}$.

By *Proposition 1*, $\overline{u_r\mathbf{z}_s} = C_r(\nu)\ \alpha_{st}^{-1}\ \alpha_{u,r}\ \mathrm{E}\{\mathbf{Z}^{(s)}_{u,r}\}$ with $\mathbf{Z}_{u,r} \sim \mathcal{TST}_p\left(\boldsymbol{\mu}, \frac{\nu}{\nu+r}\boldsymbol{\Omega}, \boldsymbol{\lambda}, \nu + r, \mathbb{A}\right)$. Then, Eq (16) follows from taking $s = 0$ (so $\mathbf{Z}^{(0)}_{u,r} = 1$) and using point *i* of *Lemma 4* on $\mathbf{Z}^{(s)}_{u,r}$ to obtain $\alpha_{u,r}$. Then, applications of point *ii* and point *iii* of *Lamma 4* to $\mathbf{Z}^{(s)}_{u,r}$ with $s = 1$ and $s = 2$ give respectively

$\mathrm{E}\{\mathbf{Z}^{(1)}_{u,r}\} = \mathbf{A}\mathrm{E}\{\mathbf{X}^{(1)}_{u,r}\}$ and $\mathrm{E}\{\mathbf{Z}^{(2)}_{u,r}\} = \mathbf{A}\mathrm{E}\{\mathbf{X}^{(2)}_{u,r}\}\mathbf{A}$ with $\mathbf{X}_{u,r} \sim \mathcal{TST}_p\left(\mathbf{A}\boldsymbol{\mu}, \frac{\nu}{\nu+r}\mathbf{A}\boldsymbol{\Omega}\mathbf{A}, \mathbf{A}\boldsymbol{\lambda}, \nu+r, \mathbf{Aa}\right)$; and prove Eq (17-18). By *Proposition 1* again, $\overline{\tau_r \mathbf{z}_s} = cMC_r(\nu)\ \alpha_{st}^{-1}\ \alpha_{\tau,r}\ \mathrm{E}\{\mathbf{Z}^{(s)}_{\tau,r}\}$, with $\mathbf{Z}_{\tau,r} \sim \mathcal{TT}_p\left(\boldsymbol{\mu}, \frac{\nu}{\nu+r}\bar{\boldsymbol{\Omega}}, \nu+r, \mathbb{A}\right)$. Then, Eq (19-21) follows by the same argument developed above for Eq (16-18).

The results for the general case where $\nu_0 \in (0, \infty)$ is obtained by replacing $\boldsymbol{\Omega}$ by $\frac{\nu_0}{\nu}\boldsymbol{\Omega}$ and U by $\frac{\nu}{\nu_0}U$ in the results for the special case $\nu_0 = \nu$.

References

1. Lachos VH, Ghosh P, Arellano-Valle RB. Likelihood based inference for skew-normal independent linear mixed models. Statistica Sinica. 2010;20:303–322.

2. Prates MO, Costa DR, Lachos VH. Generalized linear mixed models for correlated binary data with t-link. Statistics and Computing. 2014;24(6):1111–1123.

3. Gentle JE. Matrix Algebra Theory, Computations, And Applications In Statistics. Springer Science+Business Media; 2007.

4. Branco MD, Dey DK. A general class of multivariate skew-elliptical distributions. Journal of Multivariate Analysis. 2001;79(1):99–113.
