# Supplementary material for: Inference in skew generalized t-link models for clustered binary outcome via a parameter-expanded EM algorithm
Source: PLoS One. 2021 Apr 6;16(4):e0249604. doi: 10.1371/journal.pone.0249604 (PMC8028747; doi:10.1371/journal.pone.0249604)
Supplement: S5 Appendix — This supporting information gives a proof of Proposition 2. The first two moments of truncated multivariate skew normal distributions (limiting case as ν → ∞) are also given (required for fitting skew-probit link models). (PDF) [file pone.0249604.s005.pdf]

S5 Appendix for the manuscript "Inference in skew generalized t-link models for clustered binary outcome via a parameter-expanded EM algorithm"

Chénangnon F. Tovissodé [1*], Aliou Diop[2], Romain Glèlè Kakaï[1]

1 Laboratoire de Biomathématiques et d'Estimations Forestières, Faculté des Sciences Agronomiques, Université d'Abomey-Calavi, Abomey-Calavi, Bénin

2 Laboratoire d'Etudes et Recherches en Statistiques et Développement, Université Gaston Berger de Saint-Louis, Saint-Louis, Sénégal

* chenangnon@gmail.com

Note: Equation numbers refer to corresponding equations in the main text unless a source reference is specified.

S5 Appendix: proof and limiting case of *Proposition 2*

Proof of *Proposition 2*

Applying Theorem 1 in [1] to the right truncated skew t vector $\mathbf{Z} \sim \mathcal{TST}_p(\mathbf{0}, \mathbf{R}, \boldsymbol{\lambda}, \nu, \mathbf{a})$ yields for the p–vector of all ones $\mathbf{s} = (1, \cdots, 1)$:

$$\mathrm{E}\left\{\mathbf{Z}^{(\mathbf{s})}\right\} = \mathrm{E}\left\{\mathbf{X}^{(\bar{\mathbf{s}})}\right\}$$

where $\mathbf{Z}^{(\mathbf{s})}$ stands for $(Z_1^{s_1}, \cdots, Z_p^{s_p})$, $\mathbf{X}$ is the right truncated multivariate t vector $\mathbf{X} \sim \mathcal{TT}_{p+1}(\mathbf{0}, \mathbf{R}^*, \nu, \bar{\mathbf{a}})$, $\mathbf{R}^* = \begin{pmatrix} \sigma_0^2 & -\sigma_0 \boldsymbol{\delta}^\top \\ -\sigma_0 \boldsymbol{\delta} & \mathbf{R} \end{pmatrix}$, $\sigma_0 = \sqrt{1 + \boldsymbol{\lambda}^\top \boldsymbol{\lambda}}$, $\boldsymbol{\delta} = \sigma_0^{-1} \mathbf{R}^{1/2} \boldsymbol{\lambda}$ (by Eq (13) in [1]); $\bar{\mathbf{a}} = (0, \mathbf{a})^\top$ and $\bar{\mathbf{s}} = (0, \mathbf{s}^\top)$ (by Eq (18) in [1]). The negative sign of

$-\sigma_0\boldsymbol{\delta}$ in the expression of the covariance matrix $\mathbf{R}^*$ results from considering a right truncation at zero for the first component (latent) of $\mathbf{X}$ instead of a left truncation in [1] (see Eq (2) therein). Note that we follow the notation in [1] so that, while $\mathbf{X}$ has $p+1$ elements, $\bar{s}_1 = 0$ implies that $\mathrm{E}\left\{\mathbf{X}^{(\bar{\mathbf{s}})}\right\}$ has only p elements (the first row of $\mathbf{X}$ is considered inactive and ignored). Since the active components of $\mathbf{X}$ are all standardized (their covariance matrix is $\mathbf{R}$), we can use Theorem 3 in [2] to obtain the first two moments of $\mathbf{X}^{(\bar{\mathbf{s}})}$:

$$\begin{aligned}\mathrm{E}\left\{\mathbf{X}^{(\bar{\mathbf{s}})}\right\} &= -\frac{\nu\alpha_{st}^{-1}}{\nu-2}\widetilde{\mathbf{R}}\tilde{q}(\bar{\mathbf{a}}) \\ \mathrm{E}\left\{\mathbf{X}^{(\bar{\mathbf{s}})}\mathbf{X}^{(\bar{\mathbf{s}})\ \top}\right\} &= \frac{\nu\alpha_{st}^{-1}}{\nu-2}\left[\alpha_{st}^{*}\mathbf{R} + \widetilde{\mathbf{R}}\left(\widetilde{\mathbf{H}}+\widetilde{\mathbf{D}}\right)\widetilde{\mathbf{R}}^{\top}\right]\end{aligned}$$

where $\widetilde{\mathbf{R}} = \begin{pmatrix} -\boldsymbol{\delta} & \mathbf{R}\end{pmatrix}$, $\tilde{q}(\bar{\mathbf{a}}) = (C_0^*, q^*(\mathbf{a})^\top)^\top$, $\widetilde{\mathbf{H}} = \begin{pmatrix} 0 & \mathbf{H}_0^{*\top} \\ \mathbf{H}_0^* & \mathbf{H}^*\end{pmatrix}$, $\widetilde{\mathbf{D}} = \begin{pmatrix} D_0^* & \mathbf{0}^* \\ \mathbf{0} & \mathbf{D}^*\end{pmatrix}$, α_{st}^*, C_0^*, $q^*(\mathbf{a})$, $\mathbf{H}^*$, D_0^* and $\mathbf{D}^*$ are as defined in *Proposition 2*. A direct matrix operation then yields the results. □

Limiting case of *Proposition 2*

When $\nu \to \infty$, the right truncated multivariate SGT family has the truncated multivariate skew normal (SN) family as a limiting case. We define here the right truncated multivariate SN family and give formulas for computing its first two moments.

Because an SGT vector can be represented as a scale mixture of skew normal (SN) vectors via the representation in Eq (2), we start with the moments of right truncated multivariate SN distributions as a preliminary result to prove *Proposition 2*. The $p-$variate SN distribution with location $\boldsymbol{\mu}$, scale $\boldsymbol{\Omega}$, and shape $\boldsymbol{\lambda}$, denoted $\mathcal{SN}_p(\boldsymbol{\mu}, \boldsymbol{\Omega}, \boldsymbol{\lambda})$ has pdf [3] (page 305, Eq 2.1):

$$Sn_p(\mathbf{y}|\boldsymbol{\mu}, \boldsymbol{\Omega}, \boldsymbol{\lambda}) = 2\ \Phi(\boldsymbol{\lambda}^\top \mathbf{y}_0)\ \phi_p(\mathbf{y}|\boldsymbol{\mu}, \boldsymbol{\Omega}) \text{ with } \mathbf{y}_0 = \boldsymbol{\Omega}^{-1/2}(\mathbf{y}-\boldsymbol{\mu}) \qquad (A.1)$$

where $\phi_p(\cdot|\boldsymbol{\mu}, \boldsymbol{\Omega})$ is the pdf of the $p-$variate normal distribution with mean $\boldsymbol{\mu}$ and covariance matrix $\boldsymbol{\Omega}$.

Let $\mathcal{TSN}_p(\boldsymbol{\mu}, \boldsymbol{\Omega}, \boldsymbol{\lambda}, \mathbf{a})$ represent a p-dimensional SN distribution restricted to a right

truncated hyperplane $\mathbb{A} = \{\mathbf{x} = (x_1, \cdots, x_p)^\top \mid x_1 \leq a_1, \cdots, x_p \leq a_p\}$ with $\mathbf{a} = (a_1, \cdots, a_p)^\top$. The pdf of $\mathbf{X} \sim \mathcal{TSN}_p(\boldsymbol{\mu}, \boldsymbol{\Omega}, \boldsymbol{\lambda}, \mathbf{a})$ is given by:

$$TSn_p(\mathbf{y}|\boldsymbol{\mu}, \boldsymbol{\Omega}, \boldsymbol{\lambda}, \mathbf{a}) = \alpha_{sn}^{-1} \; \Phi(\boldsymbol{\lambda}^\top \mathbf{y}_0) \; \phi_p(\mathbf{y}|\boldsymbol{\mu}, \boldsymbol{\Omega}) \; \mathbf{I}_{\mathbb{A}}(\mathbf{y}) \qquad (A.2)$$

where $\alpha_{sn} = \frac{1}{2} SN_p(\mathbf{a}|\boldsymbol{\mu}, \boldsymbol{\Omega}, \boldsymbol{\lambda})$ with $SN_p(\cdot|\boldsymbol{\mu}, \boldsymbol{\Omega}, \boldsymbol{\lambda})$ the cdf of the $p-$variate skew normal distribution. If $\boldsymbol{\mu} = \mathbf{0}$ and $\boldsymbol{\Omega}$ is a correlation matrix ($\boldsymbol{\Omega} = \mathbf{R}$), then $\mathbf{X} \sim \mathcal{TSN}_p(\mathbf{0}, \mathbf{R}, \boldsymbol{\lambda}, \mathbf{a})$. In this case, the first two moments of $\mathbf{X}$ can be evaluated using the following proposition.

Proposition 6 Let $\mathbf{X} \sim \mathcal{TSN}_p(\mathbf{0}, \mathbf{R}, \boldsymbol{\lambda}, \mathbf{a})$ with $\mathbf{R}$ a correlation matrix and set $\sigma_0 = \sqrt{1 + \boldsymbol{\lambda}^\top \boldsymbol{\lambda}}$ and $\boldsymbol{\delta} = \sigma_0^{-1} \mathbf{R}^{1/2} \boldsymbol{\lambda}$, $\mathbf{R}^* = \begin{pmatrix} \sigma_0^2 & -\sigma_0 \boldsymbol{\delta}^\top \\ -\sigma_0 \boldsymbol{\delta} & \mathbf{R} \end{pmatrix}$. Then, the first two moments of $\mathbf{X}$ are:

$$\begin{aligned} \mathrm{E}\{\mathbf{X}\} &= \alpha_{sn}^{-1} \; [C_0(\mathbf{a})\boldsymbol{\delta} - \mathbf{R} \; q(\mathbf{a})], \quad \text{and} & (A.3) \\ \mathrm{E}\{\mathbf{X}\mathbf{X}^\top\} &= \mathbf{R} + \alpha_{sn}^{-1} \; \left[\mathbf{R}(\mathbf{H} + \mathbf{D})\mathbf{R} - \mathbf{R}\mathbf{H}_0 \boldsymbol{\delta}^\top - \boldsymbol{\delta}\mathbf{H}_0^\top \mathbf{R} + D_0 \boldsymbol{\delta}\boldsymbol{\delta}^\top\right] & (A.4) \end{aligned}$$

where $C_0(\mathbf{a}) = \frac{1}{\sqrt{2\pi}} \Phi_p\left(\mathbf{a}|\mathbf{0}, \mathbf{R} - \boldsymbol{\delta}\boldsymbol{\delta}^\top\right)$ with $\Phi_p(\cdot|\boldsymbol{\mu}, \boldsymbol{\Omega})$ the cdf of the $p-$variate normal distribution with mean $\boldsymbol{\mu}$ and variance-covariance matrix $\boldsymbol{\Omega}$; $\alpha_{sn} = \Phi_{p+1}\left(\bar{\mathbf{a}}|\mathbf{0}, \mathbf{R}^*\right)$; $q(\mathbf{a}) = (q_1(a_1), \cdots, q_p(a_p))^\top$ with elements $q_i(a_i) = \phi\left(a_i\right) \Phi_p\left(\bar{\mathbf{a}}_2^{(i)}|a_i \bar{\mathbf{R}}_{12}^{(i)}, \bar{\mathbf{R}}_{22.1}^{(i)}\right)$; $\mathbf{H}$ is the $p \times p$ matrix with diagonal elements $\mathbf{H}_{ii} = 0$ and off diagonal elements defined as $\mathbf{H}_{ij} = \phi_2\left(\mathbf{a}_1^{(ij)}|\mathbf{0}, \mathbf{R}_{11}^{(ij)}\right) \Phi_{p-1}\left(\bar{\mathbf{a}}_2^{(ij)}|\bar{\boldsymbol{\mu}}_{2.1}^{(ij)}, \bar{\mathbf{R}}_{22.1}^{(ij)}\right)$; $\mathbf{H}_0$ is the $p-$vector with elements defined as: $\mathbf{H}_{0i} = \phi_2\left(a_1^{(0i)}|\mathbf{0}, \mathbf{R}_{11}^{(0i)}\right) \Phi_{p-1}\left(\bar{\mathbf{a}}_2^{(0i)}|\bar{\boldsymbol{\mu}}_{2.1}^{(0i)}, \bar{\mathbf{R}}_{22.1}^{(0i)}\right)$; $D_0 = \boldsymbol{\delta}^\top \mathbf{H}_0$; $\mathbf{D}$ is the $p \times p$ diagonal matrix with diagonal elements $\mathbf{D}_{ii} = \delta_i \mathbf{H}_{0i} - a_i q_i(a_i) - \mathbf{R}_i \mathbf{H}^i$; and $\bar{\mathbf{a}} = (0, \mathbf{a})^\top$, $\mathbf{R}_{11}^{(0i)} = \begin{pmatrix} 1 & -\delta_i \\ -\delta_i & 1 \end{pmatrix}$, $\mathbf{R}_{11}^{(ij)} = \begin{pmatrix} 1 & \rho_{ij} \\ \rho_{ij} & 1 \end{pmatrix}$, $\bar{\mathbf{R}} = \begin{pmatrix} 1 & -\boldsymbol{\delta}^\top \\ -\boldsymbol{\delta} & \mathbf{R} \end{pmatrix}$, with δ_i the ith element of $\boldsymbol{\delta}$, ρ_{ij} the $(ij)^{th}$ element of $\mathbf{R}$; $\mathbf{H}^i$ the ith column of $\mathbf{H}$; $\bar{\mathbf{a}}_2^{(i)}$ the vector $\bar{\mathbf{a}}$ with its $(i+1)^{th}$ element ($i.e.$ a_i) deleted; $\bar{\mathbf{R}}_{12}^{(i)}$ the $(i+1)^{th}$ column of $\bar{\mathbf{R}}$ with its $(i+1)^{th}$ element ($i.e.$ 1) deleted; $\bar{\mathbf{R}}_{22.1}^{(i)} = \bar{\mathbf{R}}_{22}^{(i)} - \bar{\mathbf{R}}_{12}^{(i)} \bar{\mathbf{R}}_{12}^{(i)T}$, $\bar{\mathbf{R}}_{22}^{(i)}$ being $\bar{\mathbf{R}}$ with its $(i+1)^{th}$ row and column deleted; $\mathbf{a}_1^{(ij)} = (a_i, a_j)^\top$; $\bar{\mathbf{a}}_2^{(ij)}$ the vector $\bar{\mathbf{a}}$ with its $(i+1)^{th}$ and $(j+1)^{th}$ elements ($i.e.$ a_i and a_j) deleted; $\bar{\mathbf{R}}_{22.1}^{(ij)} = \bar{\mathbf{R}}_{22}^{(ij)} - \bar{\mathbf{R}}_{12}^{(ij)} \left[\mathbf{R}_{11}^{(ij)}\right]^{-1} \bar{\mathbf{R}}_{12}^{(ij)T}$, $\bar{\mathbf{R}}_{22}^{(ij)}$ being $\bar{\mathbf{R}}$ with its $(i+1)^{th}$ and $(j+1)^{th}$ rows and columns deleted; $\bar{\mathbf{R}}_{12}^{(ij)}$ being

the matrix $\bar{\mathbf{R}}$ with its $(i+1)^{th}$ and $(j+1)^{th}$ columns deleted, and only its $(i+1)^{th}$ and $(j+1)^{th}$ rows kept; $\bar{\boldsymbol{\mu}}_{2.1}^{(ij)} = \bar{\mathbf{R}}_{12}^{(ij)} \left[\mathbf{R}_{11}^{(ij)}\right]^{-1} \mathbf{a}_1^{(ij)}$; $\mathbf{a}_1^{(0i)} = (0, a_i)^\top$; $\bar{\mathbf{a}}_2^{(0i)}$ the vector $\mathbf{a}$ with its ith element (*i.e.* a_i) deleted; $\bar{\mathbf{R}}_{22.1}^{(0i)} = \bar{\mathbf{R}}_{22}^{(0i)} - \bar{\mathbf{R}}_{12}^{(0i)} \left[\mathbf{R}_{11}^{(0i)}\right]^{-1} \bar{\mathbf{R}}_{12}^{(0i)T}$, $\bar{\mathbf{R}}_{22}^{(0i)}$ being $\mathbf{R}$ with its ith row and column deleted; $\bar{\mathbf{R}}_{12}^{(0i)}$ being the matrix $\bar{\mathbf{R}}$ with its first and $(i+1)^{th}$ columns deleted, and only its first and $(i+1)^{th}$ rows kept; and $\bar{\boldsymbol{\mu}}_{2.1}^{(0i)} = \bar{\mathbf{R}}_{12}^{(0i)} \left[\mathbf{R}_{11}^{(0i)}\right]^{-1} \mathbf{a}_1^{(0i)}$.

The proof of *Proposition 6* follows the path of the proof for *Proposition 2*, except that the cumulative probabilities of the multivariate t distribution are replaced by the cumulative probabilities of the multivariate normal distribution. The following corollary gives the first two moments of a general right truncated SN vector $\mathbf{X} \sim \mathcal{TSN}_p(\boldsymbol{\mu}, \boldsymbol{\Omega}, \boldsymbol{\lambda}, \mathbf{a})$.

Corollary 3 Let $\mathbf{X} \sim \mathcal{TSN}_p(\boldsymbol{\mu}, \boldsymbol{\Omega}, \boldsymbol{\lambda}, \mathbf{a})$. Then,

$$\mathrm{E}\{\mathbf{X}\} = \boldsymbol{\mu} + \boldsymbol{\Lambda}\mathrm{E}\{\mathbf{Z}\} \qquad (A.5)$$

$$\mathrm{E}\{\mathbf{X}\mathbf{X}^\top\} = \boldsymbol{\mu}\boldsymbol{\mu}^\top + \boldsymbol{\Lambda}\mathrm{E}\{\mathbf{Z}\}\boldsymbol{\mu}^\top + \boldsymbol{\mu}\mathrm{E}\{\mathbf{Z}^\top\}\boldsymbol{\Lambda} + \boldsymbol{\Lambda}\mathrm{E}\{\mathbf{Z}\mathbf{Z}^\top\}\boldsymbol{\Lambda} \qquad (A.6)$$

where $\boldsymbol{\Lambda} = \mathrm{diag}(\omega_1, \cdots, \omega_p)$, ω_i^2 is the ith diagonal element of $\boldsymbol{\Omega}$, $\mathbf{Z} \sim \mathcal{TSN}_p(\mathbf{0}, \mathbf{R}, \boldsymbol{\lambda}^*, \mathbf{a}^*)$, $\mathbf{R}$ is the correlation matrix from $\boldsymbol{\Omega}$, $\boldsymbol{\lambda}^* = \mathbf{R}^{-1/2}\boldsymbol{\Lambda}^{-1}\boldsymbol{\Omega}^{1/2}\boldsymbol{\lambda}$, $\mathbf{a}^* = \boldsymbol{\Lambda}^{-1}(\mathbf{a} - \boldsymbol{\mu})$ and $\mathrm{E}\{\mathbf{Z}\}$ and $\mathrm{E}\{\mathbf{Z}\mathbf{Z}^\top\}$ are available from *Proposition 6.*

References

1. Galarza CE, Matos LA, Lachos VH. Moments of the doubly truncated selection elliptical distributions with emphasis on the unified multivariate skew-t distribution. arXiv preprint arXiv:200714980. 2020;.

2. Ho HJ, Lin TI, Chen HY, Wang WL. Some results on the truncated multivariate t distribution. Journal of Statistical Planning and Inference. 2012;142(1):25–40.

3. Lachos VH, Ghosh P, Arellano-Valle RB. Likelihood based inference for skew-normal independent linear mixed models. Statistica Sinica. 2010;20:303–322.
