# Supplementary material for: Inference in skew generalized t-link models for clustered binary outcome via a parameter-expanded EM algorithm
Source: PLoS One. 2021 Apr 6;16(4):e0249604. doi: 10.1371/journal.pone.0249604 (PMC8028747; doi:10.1371/journal.pone.0249604)
Supplement: S6 Appendix — This supporting information gives a proof of Proposition 3. (PDF) [file pone.0249604.s006.pdf]

S6 Appendix for the manuscript "Inference in skew generalized t-link models for clustered binary outcome via a parameter-expanded EM algorithm"

Chénangnon F. Tovissodé [1*], Aliou Diop[2], Romain Glèlè Kakaï[1]

1 Laboratoire de Biomathématiques et d'Estimations Forestières, Faculté des Sciences Agronomiques, Université d'Abomey-Calavi, Abomey-Calavi, Bénin

2 Laboratoire d'Etudes et Recherches en Statistiques et Développement, Université Gaston Berger de Saint-Louis, Saint-Louis, Sénégal

* chenangnon@gmail.com

Note: Equation numbers refer to corresponding equations in the main text unless a source reference is specified.

S6 Appendix: proof of ***Proposition 3***

We notice from Eq (31) that the latent variable $\mathbf{Z}_i$ has the representation Eq (2) namely:

$$
\begin{aligned}
\mathbf{Z}_i &\overset{d}{=} \boldsymbol{\eta}_i - \nu_0 c\widetilde{U}_1 \delta_\varepsilon \mathbf{J}_{n_i} + \; U_i^{-1/2}(\nu_0 \delta_\varepsilon \mathbf{J}_{n_i} V_i + \mathbf{T}_i) \\
&\overset{d}{=} \left[\mathbf{X}_i\boldsymbol{\beta} + \mathbf{W}_i\left(-c\widetilde{U}_1\boldsymbol{\delta} + U_i^{-1/2}(\boldsymbol{\delta}V_i + \mathbf{S}_i)\right) - \nu_0 c\widetilde{U}_1 \delta_\varepsilon \mathbf{J}_{n_i}\right] \\
&\quad + U_i^{-1/2}(\nu_0 \delta_\varepsilon \mathbf{J}_{n_i} V_i + \mathbf{T}_i) \\
&\overset{d}{=} \left[\mathbf{X}_i\boldsymbol{\beta} - c\widetilde{U}_1\left(\upsilon_0\delta_\varepsilon\mathbf{J}_{n_i} + \mathbf{W}_i\boldsymbol{\delta}\right)\right] + U_i^{-1/2}\left[\left(\upsilon_0\delta_\varepsilon\mathbf{J}_{n_i} + \mathbf{W}_i\boldsymbol{\delta}\right)V_i + \mathbf{W}_i\mathbf{S}_i + \mathbf{T}_i\right] \\
&\overset{d}{=} \left[\mathbf{X}_i\boldsymbol{\beta} - c\widetilde{U}_1\left(\upsilon_0\delta_\varepsilon\mathbf{J}_{n_i} + \mathbf{W}_i\boldsymbol{\delta}\right)\right] + U_i^{-1/2}\left[\left(\upsilon_0\delta_\varepsilon\mathbf{J}_{n_i} + \mathbf{W}_i\boldsymbol{\delta}\right)V_i + \mathbf{T}_i^*\right] \\
&\overset{d}{=} \boldsymbol{\mu}_i + U_i^{-1/2}\left[\Delta_i V_i + \mathbf{T}_i^*\right]
\end{aligned}
$$

where we have subsequently used $\mathbf{T}_i \sim \mathcal{N}_{n_i}(0, \upsilon_0^2 \mathbf{I}_{n_i})$, $\mathbf{S}_i \sim \mathcal{N}_q(\mathbf{0}, \bar{\mathbf{D}})$ and $\mathbf{T}_i^* \overset{d}{=} \mathbf{W}_i\mathbf{S}_i + \mathbf{T}_i$ so that $\mathbf{T}_i^* \sim \mathcal{N}_{n_i}\left(\mathbf{0}, \bar{\boldsymbol{\Omega}}_i\right)$, and $V_i \sim \mathcal{HN}(0,1)$ and $U_i \sim \mathcal{G}amma(\nu/2, \nu/2)$ are independent. Hence we have $\mathbf{Z}_i \sim \mathcal{ST}_{n_i}(\boldsymbol{\mu}_i, \boldsymbol{\Omega}_i, \boldsymbol{\lambda}_i, \nu)$ where the shape $\boldsymbol{\lambda}_i = \boldsymbol{\Omega}_i^{-1/2}\boldsymbol{\Delta}_i\left(1 + \boldsymbol{\Delta}_i^\top \bar{\boldsymbol{\Omega}}_i^{-1}\boldsymbol{\Delta}_i\right)^{1/2}$ is found using Eq (8).

Furthermore, from the relation $Y_{ij} = I_{(0,\infty)}(Z_{ij})$ in Eq (31), the probability for observing $\mathbf{Y}_i = \mathbf{y}_i$ is $f_i(\mathbf{y}_i|\boldsymbol{\theta}) = \int_{\mathbb{A}_i} St_p\left(\mathbf{y}|\boldsymbol{\mu}, \boldsymbol{\Omega}, \boldsymbol{\lambda}, \nu\right) d\mathbf{y}$ where $\mathbb{A}_i = \mathbb{A}_{i1} \times \mathbb{A}_{i2} \times \cdots \times \mathbb{A}_{ip}$ with $\mathbb{A}_{ij} = (-\infty, 0]$ if $\mathbf{y}_{ij} = 0$ and $\mathbb{A}_{ij} = (0, \infty)$ if $\mathbf{y}_{ij} = 1$. Then, by point i of *Lemma 4* (see S4 Appendix), $f_i(\mathbf{y}_i|\boldsymbol{\theta}) = ST_{n_i}\left(\mathbf{0}|\mathbf{A}_i\boldsymbol{\mu}_i, \mathbf{A}_i\boldsymbol{\Omega}_i\mathbf{A}_i, \mathbf{A}_i\boldsymbol{\lambda}_i, \nu\right)$ with $\mathbf{A}_i = diag(A_{i1}, \cdots, A_{in_i})$ where $A_{ij} = 1$ if $\mathbb{A}_{ij} = (-\infty, 0]$ and $A_{ij} = -1$ if $\mathbb{A}_{ij} = (0, \infty)$, *i.e.* $A_{ij} = 1$ if $\mathbf{y}_{ij} = 0$ and $A_{ij} = -1$ if $\mathbf{y}_{ij} = 1$ which is compactly expressed as $A_{ij} = 1 - 2y_{ij}$, thereby proving Eq (32). To obtain $f_{ij}(y_{ij}|\boldsymbol{\theta})$, we marginalize Z_{ij} from $\mathbf{Z}_i$: $Z_{ij} \sim \mathcal{ST}(\boldsymbol{\mu}_{ij}, \omega_{ij}^2, \lambda_{ij}, \nu)$ where $\lambda_{ij} = \Delta_{ij}/\bar{\omega}_{ij}$ is found from the working shape parameter Δ_{ij} using Eq (8). Then, since $Y_{ij} = I_{(0,\infty)}(Z_{ij})$, it follows that the probability for observing $Y_{ij} = y_{ij}$ is $f_{ij}(y_{ij}|\boldsymbol{\theta}) = \int_{\mathbb{A}_{ij}} St(y|\boldsymbol{\mu}_{ij}, \omega_{ij}^2, \lambda_{ij}, \nu) dy$. Applying now point i of *Lemma 4* results in $f_{ij}(y_{ij}|\boldsymbol{\theta}) = ST\left(0|A_{ij}\mu_{ij}, \omega_{ij}^2, A_{ij}\lambda_{ij}, \nu\right)$, *i.e.* Eq (33).
