# Supplementary material for: Inference in skew generalized t-link models for clustered binary outcome via a parameter-expanded EM algorithm
Source: PLoS One. 2021 Apr 6;16(4):e0249604. doi: 10.1371/journal.pone.0249604 (PMC8028747; doi:10.1371/journal.pone.0249604)
Supplement: S7 Appendix — This supporting information gives a proof of Proposition 4. (PDF) [file pone.0249604.s007.pdf]

S7 Appendix for the manuscript "Inference in skew generalized t-link models for clustered binary outcome via a parameter-expanded EM algorithm"

Chénangnon F. Tovissodé [1*], Aliou Diop[2], Romain Glèlè Kakaï[1]

1 Laboratoire de Biomathématiques et d'Estimations Forestières, Faculté des Sciences Agronomiques, Université d'Abomey-Calavi, Abomey-Calavi, Bénin

2 Laboratoire d'Etudes et Recherches en Statistiques et Développement, Université Gaston Berger de Saint-Louis, Saint-Louis, Sénégal

* chenangnon@gmail.com

Note: Equation numbers refer to corresponding equations in the main text unless a source reference is specified.

S7 Appendix: proof of *Proposition 4*

For simplicity, we have omitted the "hat" used in the proposition to indicate that all quantities must be evaluated by substituting $\widehat{\boldsymbol{\theta}}$ for $\boldsymbol{\theta}$. The proof consists in first deriving from Eq (31) the conditional distributions $(\mathbf{b}_i|V_i = v_i, U_i = u_i, \mathbf{Z}_i = \mathbf{z}_i)$, $(V_i|U_i = u_i, \mathbf{Z}_i = \mathbf{z}_i)$ and $(\mathbf{Z}_i|\mathbf{y}_i)$, and repeatedly using the law of iterated expectations. From *Proposition 3*, the latent vector $\mathbf{Z}_i$ has given $V_i = v_i$ and $U_i = u_i$, a normal distribution with mean $\boldsymbol{\mu}_i + (v_i u_i^{-1/2} - c\widetilde{U}_1)\boldsymbol{\Delta}_i$ and variance-covariance matrix $u_i^{-1}\bar{\boldsymbol{\Omega}}_i$ (a). Moreover, from Eq (31), given $V_i = v_i$ and $U_i = u_i$, the random effects vector $\mathbf{b}_i$ has a normal distribution with mean $(v_i u_i^{-1/2} - c\widetilde{U}_1)\boldsymbol{\delta}$ and variance-covariance matrix $u_i^{-1}\bar{\mathbf{D}}$ (b). In addition, Eq (31) indicates that conditional on $V_i = v_i$ and $U_i = u_i$, $\mathbf{Z}_i$ can be written as a linear combination of $\mathbf{b}_i$: $\mathbf{Z}_i = \mathbf{X}_i\boldsymbol{\beta} + \mathbf{W}_i\mathbf{b}_i + (v_i u_i^{-1/2} - c\widetilde{U}_1)\delta_\epsilon\mathbf{J}_{n_i} + \bar{\mathbf{Z}}_i$ where $\bar{\mathbf{Z}}_i \sim \mathcal{N}_{n_i}\left(\mathbf{0}, u_i^{-1}\bar{\boldsymbol{\Omega}}_i\right)$, hence the covariance of $\mathbf{Z}_i$ and $\mathbf{b}_i$ given $V_i = v_i$, and $U_i = u_i$ is $u_i^{-1}\mathbf{W}_i\bar{\mathbf{D}}$ (c). By combining the above (a), (b) and (c) statements, the joint distribution of $\left(\mathbf{b}_i^\top, \mathbf{Z}_i^\top\right)^\top$ given $V_i = v_i$ and $U_i = u_i$ turns to be normal with conditional

mean $\begin{pmatrix} -c\widetilde{U}_1\boldsymbol{\delta} + v_i u_i^{-1/2}\boldsymbol{\delta} \\ \boldsymbol{\mu}_i + v_i u_i^{-1/2}\boldsymbol{\Delta}_i \end{pmatrix}$ and covariance matrix $u_i^{-1}\begin{pmatrix} \bar{\mathbf{D}} & \bar{\mathbf{D}}\mathbf{W}_i^\top \\ \mathbf{W}_i\bar{\mathbf{D}} & \bar{\boldsymbol{\Omega}}_i \end{pmatrix}$. Hence, by Eq (5) and Eq (6) in [1] (page 34), $(\mathbf{b}_i|V_i = v_i, U_i = u_i, \mathbf{Z}_i = \mathbf{z}_i)$, is normal with mean $\mathbf{m}_{b_i} = (v_i u_i^{-1/2} - c\widetilde{U}_1)\boldsymbol{\delta} + \bar{\mathbf{D}}\mathbf{W}_i^\top\bar{\boldsymbol{\Omega}}_i^{-1}[\mathbf{z}_i - (\boldsymbol{\mu}_i + v_i u_i^{-1/2}\boldsymbol{\Delta}_i)]$ and variance-covariance matrix $u_i^{-1}\boldsymbol{\Lambda}_i$ where $\boldsymbol{\Lambda}_i = \bar{\mathbf{D}} - \bar{\mathbf{D}}\mathbf{W}_i^\top\bar{\boldsymbol{\Omega}}_i^{-1}\mathbf{W}_i\bar{\mathbf{D}}$. On using $\boldsymbol{\mu}_i = \mathbf{X}_i\boldsymbol{\beta} - c\widetilde{U}_1\boldsymbol{\Delta}_i$ with $\mathbf{r}_i = \bar{\mathbf{D}}\mathbf{W}_i^\top\bar{\boldsymbol{\Omega}}_i^{-1}$ and $\mathbf{s}_i = \boldsymbol{\delta} - \mathbf{r}_i\boldsymbol{\Delta}_i$, we have $\mathbf{m}_{b_i} = \mathbf{r}_i(\mathbf{z}_i - \mathbf{X}_i\boldsymbol{\beta}) + \left(v_i u_i^{-1/2} - c\widetilde{U}_1\right)\mathbf{s}_i$ and $\boldsymbol{\Lambda}_i = (\mathbf{I}_q - \mathbf{r}_i\mathbf{W}_i)\,\bar{\mathbf{D}}$. It then comes by the law of iterated expectations that:

$$\begin{aligned}
\mathrm{E}\{U_i\mathbf{b}_i|\mathbf{y}_i\} &= \mathrm{E}\{U_i\mathrm{E}\{\mathbf{b}_i|V_i = v_i, U_i = u_i, \mathbf{Z}_i = \mathbf{z}_i\}|\mathbf{Y}_i = \mathbf{y}_i\} = \mathrm{E}\{U_i\ \mathbf{m}_{b_i}|\mathbf{y}_i\} \\
&= \mathbf{r}_i\left[\mathrm{E}\{U_i\mathbf{Z}_i|\mathbf{y}_i\} - \mathrm{E}\{U_i|\mathbf{y}_i\}\mathbf{X}_i\boldsymbol{\beta}\right] \\
&\quad + \left[\mathrm{E}\{V_iU_i^{1/2}|\mathbf{y}_i\} - c\widetilde{U}_1\mathrm{E}\{U_i|\mathbf{y}_i\}\right]\mathbf{s}_i, \text{and} \\
\mathrm{E}\{V_iU_i^{1/2}\mathbf{b}_i|\mathbf{y}_i\} &= \mathrm{E}\{V_iU_i^{1/2}\ \mathbf{m}_{b_i}|\mathbf{y}_i\} \\
&= \mathbf{r}_i\left[\mathrm{E}\{V_iU_i^{1/2}\mathbf{Z}_i|\mathbf{y}_i\} - \mathrm{E}\{V_iU_i^{1/2}|\mathbf{y}_i\}\mathbf{X}_i\boldsymbol{\beta}\right] \\
&\quad + \left[\mathrm{E}\{V_i^2|\mathbf{y}_i\} - c\widetilde{U}_1\mathrm{E}\{V_iU_i^{1/2}|\mathbf{y}_i\}\right]\mathbf{s}_i
\end{aligned}$$

which yield Eq (40–42). We likely have:

$$\begin{aligned}
\mathrm{E}\{U_i\mathbf{b}_i\mathbf{b}_i^\top|\mathbf{y}_i\} &= \mathrm{E}\{U_i\left[\mathbf{m}_{b_i}\mathbf{m}_{b_i}^\top + U_i^{-1}\boldsymbol{\Lambda}_i\right]|\mathbf{y}_i\} \\
&= \mathbf{r}_i\left[\mathrm{E}\{U_i\mathbf{Z}_i\mathbf{Z}_i^\top|\mathbf{y}_i\} + \mathbf{X}_i\boldsymbol{\beta}(\mathrm{E}\{U_i|\mathbf{y}_i\}\boldsymbol{\beta}^\top\mathbf{X}_i^\top - \mathrm{E}\{U_i\mathbf{Z}_i|\mathbf{y}_i^\top\})\right. \\
&\qquad \left. - \mathrm{E}\{U_i\mathbf{Z}_i|\mathbf{y}_i\}\mathbf{X}_i^\top\boldsymbol{\beta}^\top\right]\mathbf{r}_i^\top \\
&\quad + \mathbf{r}_i\left[\mathrm{E}\{V_iU_i^{1/2}\mathbf{Z}_i|\mathbf{y}_i\} - \mathrm{E}\{V_iU_i^{1/2}|\mathbf{y}_i\}\mathbf{X}_i\boldsymbol{\beta}\right. \\
&\qquad \left. - c\widetilde{U}_1\left(\mathrm{E}\{U_i\mathbf{Z}_i|\mathbf{y}_i\} - \mathrm{E}\{U_i|\mathbf{y}_i\}\mathbf{X}_i\boldsymbol{\beta}\right)\right]\mathbf{s}_i^\top \\
&\quad + \mathbf{s}_i\left[\mathrm{E}\{V_iU_i^{1/2}\mathbf{Z}_i|\mathbf{y}_i\} - \mathrm{E}\{V_iU_i^{1/2}|\mathbf{y}_i\}\mathbf{X}_i\boldsymbol{\beta}\right. \\
&\qquad \left. - c\widetilde{U}_1\left(\mathrm{E}\{U_i\mathbf{Z}_i|\mathbf{y}_i\} - \mathrm{E}\{U_i|\mathbf{y}_i\}\mathbf{X}_i\boldsymbol{\beta}\right)\right]^\top\mathbf{r}_i^\top \\
&\quad + \left[\mathrm{E}\{V_i^2|\mathbf{y}_i\} - 2c\widetilde{U}_1\mathrm{E}\{V_iU_i^{1/2}|\mathbf{y}_i\} + c^2\widetilde{U}_1^2\mathrm{E}\{U_i|\mathbf{y}_i\}\right]\mathbf{s}_i\mathbf{s}_i^\top + \boldsymbol{\Lambda}_i
\end{aligned}$$

which yields Eq (43). Next, from *Proposition 3*, the marginal distribution of $\mathbf{Z}_i$ is $\mathcal{ST}_{n_i}(\boldsymbol{\mu}_i, \boldsymbol{\Omega}_i, \boldsymbol{\lambda}_i, \nu)$. The joint pdf of V_i, U_i and $\mathbf{Z}_i$ is thus by Bayes's rule $f_{V_i,U_i,\mathbf{Z}_i}(\mathbf{z}_i, u_i, v_i) = f_{\mathbf{Z}_i|U_i,V_i}(\mathbf{z}_i|u_i, v_i) \times f_{U_i}(u_i) \times f_{V_i}(v_i)$ because U_i and V_i are independent. Using the representation Eq (2) for $\mathbf{Z}_i \sim \mathcal{ST}_{n_i}(\boldsymbol{\mu}_i, \boldsymbol{\Omega}_i, \boldsymbol{\lambda}_i, \nu)$, $\mathbf{Z}_i$ appears to be normal with pdf $\phi_{n_i}\left(\mathbf{z}_i|\boldsymbol{\mu}_i + v_i u_i^{-1/2}\boldsymbol{\Delta}_i, u_i^{-1}\ \bar{\boldsymbol{\Omega}}_i\right)$ given $V_i = v_i$ and $U_i = v_i$. The joint pdf of V_i, U_i and $\mathbf{Z}_i$ is thus $f_{V_i,U_i,\mathbf{Z}_i}(v_i, u_i, \mathbf{z}_i) =$ $\phi_{n_i}\left(\mathbf{z}_i|\boldsymbol{\mu}_i + v_i u_i^{-1/2}\boldsymbol{\Delta}_i, u_i^{-1}\ \bar{\boldsymbol{\Omega}}_i\right) \times f_G(u_i|\nu/2, \nu/2) \times \phi(v_i) \times I_{(0,\infty)}(v_i)$. The

conditional pdf $h_{v_i}(v_i) = f_{V_i|U_i,\mathbf{Z}_i,\mathbf{Y}_i}(v_i|u_i,\mathbf{z}_i)$ is then obtained through:

$$\begin{aligned}
h_{v_i}(v_i) &\propto \phi_{n_i}\left(\mathbf{z}_i|\boldsymbol{\mu}_i + v_i u_i^{-1/2}\boldsymbol{\Delta}_i, u_i^{-1}\bar{\boldsymbol{\Omega}}_i\right) \times \phi(v_i) \times I_{(0,\infty)}(v_i) \\
&\propto \exp\left(-\frac{u_i}{2}\left[\mathbf{z}_i - \boldsymbol{\mu}_i - v_i u_i^{-1/2}\boldsymbol{\Delta}_i\right]^\top \bar{\boldsymbol{\Omega}}_i^{-1}\left[\mathbf{z}_i - \boldsymbol{\mu}_i - v_i u_i^{-1/2}\boldsymbol{\Delta}_i\right]\right) \\
&\quad \times \exp\left(-\frac{v_i^2}{2}\right) \times I_{(0,\infty)}(v_i) \\
&\propto \exp\left(-\frac{u_i}{2}\left[-2v_i u_i^{-1/2}\boldsymbol{\Delta}_i^\top \bar{\boldsymbol{\Omega}}_i^{-1}(\mathbf{z}_i - \boldsymbol{\mu}_i) + v_i^2 u_i^{-1}\boldsymbol{\Delta}_i^\top \bar{\boldsymbol{\Omega}}_i^{-1}\boldsymbol{\Delta}_i\right] - \frac{v_i^2}{2}\right) \\
&\quad \times I_{(0,\infty)}(v_i) \\
&\propto \exp\left(-\frac{1}{2}\left[-2v_i u_i^{1/2}\boldsymbol{\Delta}_i^\top \bar{\boldsymbol{\Omega}}_i^{-1}(\mathbf{z}_i - \boldsymbol{\mu}_i) + v_i^2 \boldsymbol{\Delta}_i^\top \bar{\boldsymbol{\Omega}}_i^{-1}\boldsymbol{\Delta}_i + v_i^2\right]\right) \times I_{(0,\infty)}(v_i) \\
&\propto \exp\left(-\frac{1+\boldsymbol{\Delta}_i^\top \bar{\boldsymbol{\Omega}}_i^{-1}\boldsymbol{\Delta}_i}{2}\left[-2v_i u_i^{1/2}\left(1+\boldsymbol{\Delta}_i^\top \bar{\boldsymbol{\Omega}}_i^{-1}\boldsymbol{\Delta}_i\right)^{-1}\boldsymbol{\Delta}_i^\top \bar{\boldsymbol{\Omega}}_i^{-1}(\mathbf{z}_i - \boldsymbol{\mu}_i)\right.\right. \\
&\quad \left.\left. + v_i^2\right]\right) \times I_{(0,\infty)}(v_i)
\end{aligned}$$

which with $M_i = \left(1+\boldsymbol{\Delta}_i^\top \bar{\boldsymbol{\Omega}}_i^{-1}\boldsymbol{\Delta}_i\right)^{-1/2}$ and $\alpha_i = M_i \boldsymbol{\Delta}_i^\top \bar{\boldsymbol{\Omega}}_i^{-1}(\mathbf{z}_i - \boldsymbol{\mu}_i) = \boldsymbol{\lambda}_i^\top \boldsymbol{\Omega}_i^{-1/2}(\mathbf{z}_i - \boldsymbol{\mu}_i)$ reads:

$$\begin{aligned}
h_{v_i}(v_i) &\propto \exp\left(-\frac{1}{2M_i^2}\left(v_i^2 - 2v_i u_i^{1/2}\alpha_i M_i\right)\right) \times I_{(0,\infty)}(v_i) \\
&\propto \exp\left(-\frac{1}{2M_i^2}\left(v_i - u_i^{1/2}\alpha_i M_i\right)^2\right) \times I_{(0,\infty)}(v_i) \\
&\propto \phi\left(v_i|u_i^{1/2}\alpha_i M_i, M_i^2\right) \times I_{(0,\infty)}(v_i)
\end{aligned}$$

where the last line follows by recognizing the unnormalized pdf of a normal distribution with mean $u_i^{1/2}\alpha_i M_i$ and variance M_i^2. The normalizing constant is thus $1-\Phi\left(\frac{0-u_i^{1/2}\alpha_i M_i}{M_i}\right) = 1-\Phi(-u_i^{1/2}\alpha_i)$ which reduces to $\Phi(u_i^{1/2}\alpha_i)$ since $\Phi(-x) = 1-\Phi(x)$. Hence $h_{v_i}(v_i) = \frac{\phi\left(v_i|u_i^{1/2}\alpha_i M_i, M_i^2\right)}{\Phi\left(u_i^{1/2}\alpha_i\right)} \times I_{(0,\infty)}(v_i)$. From *Corollary 1* in [2] on moments of truncated normal variables (page 134), we readily get $\mathrm{E}\{V_i|\mathbf{y}_i\} = M_i\left[u_i^{1/2}\alpha_i + \zeta_1(u_i^{1/2}\alpha_i)\right]$ and $\mathrm{E}\{V_i^2|\mathbf{y}_i\} = M_i^2\left[1 + u_i\alpha_i^2 + u_i^{1/2}\alpha_i\ \zeta_1(u_i^{1/2}\alpha_i)\right]$ conditional on $U_i = u_i$, with $\zeta_1(x) = \phi(x)/\Phi(x)$. Then, by the law of iterated expectations, it follows that:

$$\begin{aligned}
\mathrm{E}\{V_i U_i^{1/2}|\mathbf{y}_i\} &= M_i\left[\mathrm{E}\{U_i\alpha_i|\mathbf{y}_i\} + \mathrm{E}\{U_i^{1/2}\zeta_1(U_i^{1/2}\alpha_i)|\mathbf{y}_i\}\right] \\
\mathrm{E}\{V_i^2|\mathbf{y}_i\} &= M_i^2\left[1 + \mathrm{E}\{U_i\alpha_i^2|\mathbf{y}_i\} + \mathrm{E}\{U_i^{1/2}\alpha_i\ \zeta_1(U_i^{1/2}\alpha_i)|\mathbf{y}_i\}\right]
\end{aligned}$$

which on setting $\tau_i = \mathrm{E}\{U_i^{1/2}\zeta_1(U_i^{1/2}\alpha_i)|\mathbf{y}_i\}$, $\tau\alpha_i = \mathrm{E}\{U_i^{1/2}\alpha_i\zeta_1(U_i^{1/2}\alpha_i)|\mathbf{y}_i\}$, $u_2\alpha_i = \mathrm{E}\{U_i\alpha_i|\mathbf{y}_i\}$, $u_2\alpha\mathbf{z}_i = \mathrm{E}\{U_i\alpha_i\mathbf{Z}_i|\mathbf{y}_i\}$, and $u_2\alpha_{2i} = \mathrm{E}\{U_i\alpha_i^2|\mathbf{y}_i\}$ lead to Eq (44–46).

In order to derive the conditional pdf $h_{\mathbf{Z}_i}(\mathbf{z}_i) = f_{\mathbf{z}_i|\mathbf{Y}_i}(\mathbf{z}_i)$, recall that $h_{\mathbf{Z}_i}(\mathbf{z}_i) \propto f_{\mathbf{Z}_i,\mathbf{Y}_i}(\mathbf{z}_i, \mathbf{y}_i)$ and that $f_{\mathbf{Z}_i,\mathbf{Y}_i}(\mathbf{z}_i, \mathbf{y}_i) = f_{\mathbf{Z}_i}(\mathbf{z}_i) \times f_{\mathbf{Y}_i|\mathbf{Z}_i}(\mathbf{y}_i)$ by Bayes's rule. Note from Eq (31) that $f_{\mathbf{Y}_i|\mathbf{Z}_i}(\mathbf{y}_i) = I_{\mathbb{A}_i}(\mathbf{z}_i)$ and from *Proposition 3* that $f_{\mathbf{Z}_i}(\mathbf{z}_i) = St_{n_i}(\mathbf{z}_i|\boldsymbol{\mu}_i, \boldsymbol{\Omega}_i, \boldsymbol{\lambda}_i, \nu)$. We thus have $h_{\mathbf{Z}_i}(\mathbf{z}_i) \propto I_{\mathbb{A}_i}(\mathbf{z}_i) \times St_{n_i}(\mathbf{z}_i|\boldsymbol{\mu}_i, \boldsymbol{\Omega}_i, \boldsymbol{\lambda}_i, \nu)$, which in light of Eq (13) normalizes as $h_{\mathbf{Z}_i}(\mathbf{z}_i) = TSt_{n_i}(\mathbf{z}_i|\boldsymbol{\mu}_i, \boldsymbol{\Omega}_i, \boldsymbol{\lambda}_i, \nu, \mathbb{A}_i)$ hence $\mathbf{Z}_i|\mathbf{Y}_i = \mathbf{y}_i, \boldsymbol{\theta} = \widehat{\boldsymbol{\theta}} \sim \mathcal{TST}_{n_i}\left(\widehat{\boldsymbol{\mu}}_i, \widehat{\boldsymbol{\Omega}}_i, \widehat{\boldsymbol{\lambda}}_i, \widehat{\nu}, \mathbb{A}_i\right)$.

References

1. Anderson TW. An introduction to multivariate statistical analysis. Wiley New York; 1962.

2. Horrace WC. Moments of the truncated normal distribution. Journal of Productivity Analysis. 2015;43(2):133–138.
