# Supplementary material for: Inference in skew generalized t-link models for clustered binary outcome via a parameter-expanded EM algorithm
Source: PLoS One. 2021 Apr 6;16(4):e0249604. doi: 10.1371/journal.pone.0249604 (PMC8028747; doi:10.1371/journal.pone.0249604)
Supplement: S8 Appendix — This supporting information gives a proof of Proposition 5. (PDF) [file pone.0249604.s008.pdf]

S8 Appendix for the manuscript "Inference in skew generalized t-link models for clustered binary outcome via a parameter-expanded EM algorithm"

Chénangnon F. Tovissodé [1*], Aliou Diop[2], Romain Glèlè Kakaï[1]

1 Laboratoire de Biomathématiques et d'Estimations Forestières, Faculté des Sciences Agronomiques, Université d'Abomey-Calavi, Abomey-Calavi, Bénin

2 Laboratoire d'Etudes et Recherches en Statistiques et Développement, Université Gaston Berger de Saint-Louis, Saint-Louis, Sénégal

* chenangnon@gmail.com

Note: Equation numbers refer to corresponding equations in the main text unless a source reference is specified.

S8 Appendix: proof of *Proposition 5*

The following scores of $Q(\cdot|\widehat{\boldsymbol{\theta}}^{(k)})$ are straightforwardly derived from Eq (39):

$$\begin{aligned}
\frac{\partial Q(\boldsymbol{\theta}|\widehat{\boldsymbol{\theta}}^{(k)})}{\partial \boldsymbol{\beta}} &= -\sum_{i=1}^{n} \widehat{u_2}_i^{(k)} \mathbf{X}_i^\top \mathbf{X}_i \boldsymbol{\beta} + \sum_{i=1}^{n} \mathbf{X}_i^\top \left(\widehat{\mathbf{S}_1}_i^{(k)} - \delta_\varepsilon \widehat{S_2}_i^{(k)} \mathbf{J}_{n_i} \right), \\
\frac{\partial Q(\boldsymbol{\theta}|\widehat{\boldsymbol{\theta}}^{(k)})}{\partial \delta_\varepsilon} &= -\sum_{i=1}^{n} \widehat{S_2}_i^{(k)} \mathbf{J}_{n_i}^\top \mathbf{X}_i \boldsymbol{\beta} - \delta_\varepsilon \sum_{i=1}^{n} n_i \widehat{S_3}_i^{(k)} + \sum_{i=1}^{n} \mathbf{J}_{n_i}^\top \left(\widehat{\mathbf{S}_4}_i^{(k)} - c\widetilde{U}_1 \widehat{\mathbf{S}_1}_i^{(k)} \right).
\end{aligned}$$

Expressions (47–48) then follow by setting the last two derivatives to zero and jointly solving for $\boldsymbol{\beta}$ and δ_ε. Likely, on setting $\widehat{\widehat{vu\mathbf{b}}}_i^{(k)} = \widehat{vu\mathbf{b}}_i^{(k)} - c\widetilde{U}_1 \widehat{u_2\mathbf{b}}_i^{(k)}$,

$$\begin{aligned}
\frac{\partial Q(\boldsymbol{\theta}|\widehat{\boldsymbol{\theta}}^{(k)})}{\partial \boldsymbol{\delta}} &= -\bar{\mathbf{D}}^{-1} \boldsymbol{\delta} \sum_{i=}^{n} \widehat{S_3}_i^{(k)} + \bar{\mathbf{D}}^{-1} \sum_{i=1}^{n} \widehat{\widehat{vu\mathbf{b}}}_i^{(k)}, \\
\frac{\partial Q(\boldsymbol{\theta}|\widehat{\boldsymbol{\theta}}^{(k)})}{\partial \bar{\mathbf{D}}} &= \frac{1}{2} \left\{ \bar{\mathbf{D}}^{-1} \left[\sum_{i=1}^{n} \widehat{u_2\mathbf{b}_2}_i^{(k)} - \boldsymbol{\delta} \sum_{i=1}^{n} \widehat{\widehat{vu\mathbf{b}}}_i^{(k)\top} - \sum_{i=1}^{n} \widehat{\widehat{vu\mathbf{b}}}_i^{(k)} \boldsymbol{\delta}^\top \right. \right. \\
&\quad \left. \left. + \sum_{i=1}^{n} \widehat{S_3}_i^{(k)} \boldsymbol{\delta}\boldsymbol{\delta}^\top \right] - n\mathbf{I}_q \right\} \bar{\mathbf{D}}^{-1}.
\end{aligned}$$

Setting the last two derivatives to zero and solving for $\boldsymbol{\delta}$ and $\bar{\mathbf{D}}$ yields expressions Eq (49–50). Since for an identifiable STGLM with a fixed number of degrees of freedom

ν, the expected complete log likelihood Eq (39) belongs to the regular exponential family [1], the candidate point defined by expressions Eq (47–50) corresponds to the unique maximum of Eq (39).

References

1. McLachlan G, Krishnan T. The EM algorithm and extensions. vol. 382. John Wiley & Sons; 2007.
